# Endothelial β-catenin upregulation and Y142 phosphorylation drive diabetic angiogenesis via upregulating *KDR*/*HDAC9*

**DOI:** 10.1186/s12964-024-01566-1

**Published:** 2024-03-15

**Authors:** Zhenfeng Chen, Bingqi Lin, Xiaodan Yao, Jie Weng, Jinlian Liu, Qi He, Ke Song, Chuyu Zhou, Zirui Zuo, Xiaoxia Huang, Zhuanhua Liu, Qiaobing Huang, Qiulin Xu, Xiaohua Guo

**Affiliations:** 1 Guangdong Provincial Key Laboratory of Cardiac Function and Microcirculation, Department of Pathophysiology, School of Basic Medical Sciences, Southern Medical University, Guangzhou, 510515 China; 2 National Experimental Education Demonstration Center for Basic Medical Sciences, Southern Medical University, Guangzhou, 510515 China; 3 Department of Intensive Care Unit, Guangdong Provincial People’s Hospital, Guangdong Academy of Medical Science, Southern Medical University, Guangzhou, 510515 China

**Keywords:** Diabetic angiogenesis, Advanced glycation end products, β-catenin, Phosphorylation, *KDR*, *HDAC9*, VEGFR1 isoform5, Bioymifi

## Abstract

**Background:**

Diabetic angiogenesis is closely associated with disabilities and death caused by diabetic microvascular complications. Advanced glycation end products (AGEs) are abnormally accumulated in diabetic patients and are a key pathogenic factor for diabetic angiogenesis. The present study focuses on understanding the mechanisms underlying diabetic angiogenesis and identifying therapeutic targets based on these mechanisms.

**Methods:**

In this study, AGE-induced angiogenesis serves as a model to investigate the mechanisms underlying diabetic angiogensis. Mouse aortic rings, matrigel plugs, and HUVECs or 293T cells were employed as research objects to explore this pathological process by using transcriptomics, gene promoter reporter assays, virtual screening and so on.

**Results:**

Here, we found that AGEs activated Wnt/β-catenin signaling pathway and enhanced the β-catenin protein level by affecting the expression of β-catenin degradation-related genes, such as FZDs (Frizzled receptors), LRPs (LDL Receptor Related Proteins), and AXIN1. AGEs could also mediate β-catenin Y142 phosphorylation through VEGFR1 isoform5. These dual effects of AGEs elevated the nuclear translocation of β-catenin and sequentially induced the expression of KDR (Kinase Insert Domain Receptor) and HDAC9 (Histone Deacetylase 9) by POU5F1 and NANOG, respectively, thus mediating angiogenesis. Finally, through virtual screening, Bioymifi, an inhibitor that blocks VEGFR1 isoform5-β-catenin complex interaction and alleviates AGE-induced angiogenesis, was identified.

**Conclusion:**

Collectively, this study offers insight into the pathophysiological functions of β-catenin in diabetic angiogenesis.

**Supplementary Information:**

The online version contains supplementary material available at 10.1186/s12964-024-01566-1.

## Background

Diabetes is widely recognized to be a concerning global issue, resulting in a considerable death roll and substantial medical costs. Of note, it is not diabetes itself but rather its associated complications that result in healthcare expenses and patient distress. These complications typically involve the microvascular system, including diabetic retinopathy, diabetic neuropathy, and diabetic nephropathy. In terms of pathophysiological mechanisms, a diabetic hyperglycemic environment in the body cause abnormal cellular signaling events, tissue responses, and production of harmful substances, leading to various diabetic microvascular complications [1]. Advanced glycation end products (AGEs) are the end products of Maillard reactions (non-enzymatic glycosylation reactions) between carbonyl compounds (reducing sugars such as glucose and fructose) and amino compounds (proteins, amino acids such as lysine and arginine), which are abnormally elevated in diabetic patients and play an essential role in mediating diabetic microangiopathy [2–5]. Several drugs have been developed to hinder the effect of AGEs, including aminoguanidine, which inhibits AGEs formation, alagebrium, which breaks AGEs crosslink, and inhibitors of AGEs receptors. Nevertheless, the therapeutic effectiveness of these drugs has been less than satisfactory [6]. Therefore, understanding the subsequent pathways of AGEs and exploring therapeutic targets based on these pathways have emerged as a crucial approach for clinical management of diabetic microvascular complications.

Angiogenesis refers to the process of forming new blood vessels from pre-existing ones and multiple studies have indicated that excessive angiogenesis is related to the development of different diseases such as tumors, atherosclerosis, and rheumatoid arthritis [7–10]. It is also closely related to diabetic microangiopathy, which serves as a pathological basis for several diabetic complications such as diabetic retinopathy and diabetic nephropathy [1]. Previous studies in our group preliminarily indicate that AGEs are associated with the process of angiogenesis [11, 12]. In this study, we also employed AGE-induced angiogenesis as a model of diabetic angiogenesis to deeply investigate the mechanisms underlying.

β-catenin serves as both a structural component and a signal transduction mediator. As a structural component, it binds to E-cadherin/α-catenin to form adhesive junctions and stabilize intercellular structures. Under physiological conditions, β-catenin free in the cytoplasm is phosphorylated by Axin/APC/CK1/GSK3 complex, which mediates its subsequent degradation. In the classical Wnt signaling pathway, extracellular Wnt ligands bind to FZDs and LRP5/6 co-receptors, which activate Dvl protein and inhibit Axin complex activity, thereby causing β-catenin to become stable in the cytoplasm, translocate to the nucleus and bind with nuclear transcriptional factor TCF (T Cell Factor)/LEF1 (Lymphoid Enhancer Binding Factor 1) to initiate the expression of Wnt/β-catenin target gene [13]. Meanwhile, many published studies revealed that β-catenin phosphorylation affects its degradation and its function at adhesion junction and signal transduction [14–17]. Although some reports revealed that β-catenin is involved in angiogenesis, the potential mechanisms are still not fully understood [18, 19]. Furthermore, it is unknown how β-catenin functions in AGE-induced angiogenesis.

Vascular endothelial growth factor receptor 1 (VEGFR1) is a tyrosine kinase-coupled receptor that includes three types of isoforms through alternative initiation of transcription, namely full-length transmembrane receptor (fl-VEGFR1, VEGFR1 isoform1), truncated extracellular soluble isoforms (sVEGFR1, VEGFR1 isoform2-4) and intracellular isoforms (iVEGFR1, VEGFR1 isoform5-7). The fl-VEGFR1 serves as a cell membrane-bound receptor, consisting of an extracellular, ligand-binding domain with seven immunoglobulin-like loops and a split intracellular TK domain [20]. The sVEGFR1 only contains the extracellular segment domain, which functions as an inert decoy to affect angiogenesis [20]. The iVEGFR1 only contains the phosphotransferase domain and the carboxy terminal tail of VEGFR1, which has kinase activity and has been reported to directly phosphorylate and activate SRC Y418 [21]. Additionally, it has been noted that fl-VEGFR1 interacts with β-catenin in HUVECs under VEGF treatment and mediates β-catenin phosphorylation [22]. Therefore, there may exist a possible link between iVEGFR1 and β-catenin, which remains to be investigated.

In this study, we aimed to investigate the function of β-catenin in AGE-mediated diabetic angiogenesis. Our data reveal that AGEs increase β-catenin protein level by activating the Wnt signaling pathway, mediate β-catenin Y142 phosphorylation by upregulating VEGFR1 isoform5, and enhance β-catenin translocation into the nucleus to regulate the target genes expression, which in turn promote angiogenesis. Further, we identified Bioymifi as an inhibitor that blocks VEGFR1 isoform5-β-catenin interaction, reduces β-catenin Y142 phosphorylation, and alleviates AGE-induced angiogenesis. These findings shed light on a potential therapeutic strategy for diabetic angiogenesis.

## Methods

### Reagents

Detailed information on key materials used in this study is shown in Table S1, including antibodies, chemicals, recombinant protein, and recombinant DNA.

### Experimental animals

Wild-type (WT) C57BL/6 mice aged 8 -12 weeks were obtained from the Laboratory Animal Center of Southern Medical University. All experiments with mice were performed under protocols approved by the Animal Care and Use Committee of Southern Medical University and were in accordance with the guidelines for ethical animal treatment.

Matrigel plug assay was carried out essentially based on the report of Chen et al. with slight modifications [23]. In brief, 1 × 10^6^ HUVECs were mixed with 500 µL ice-cold Matrigel (Corning, Cat# 356234) and injected into the abdominal skin. 7 days later, the Matrigel plugs were dissected out, fixed with 4% paraformaldehyde overnight, and embedded into paraffin. HUVECs were stained using Ulex europaeus agglutinin I (UEA- I; 1:200; Fluorescein-conjugated; Vector Labs) which specifically binds to human endothelial cells.

Mouse aortic ring assay was performed according to the procedure reported by Baker et al. [24]. In brief, mouse was euthanized by cervical dislocation and the thoracic cavity was opened to expose the aorta. Then, the aorta was separated and transferred into Opti-MEM. The blood in the aorta was flushed out with Opti-MEM, and the surrounding fat and branching vessels were carefully removed. Subsequently, the aorta was cut into rings of ~ 1.0 mm in length and serum-starved overnight, followed by embedding in collagen type I and processing as study design.

### Cell lines

HUVECs and 293T cells were purchased from ScienCell Research Laboratories (CA, USA, Cat# 8000) and Procell Life Science & Technology (Wuhan, China, Cat# CL-0005), respectively. The HUVECs were cultured in endothelial cell medium (ECM; ScienCell, Cat# 1001) with 5% fetal bovine serum (FBS), 1% endothelial cell growth supplement (ECGS), and 1% penicillin/streptomycin solution (P/S). 293T cells were cultured in DMEM/F12 medium (Gibco) supplemented with 10% FBS (ExCell).

### Preparation of advanced glycation end products (AGEs)

AGEs were prepared according to the method outlined by Schmidt AM et al. with minor adjustments [25]. In brief, bovine serum albumin (BSA) at a concentration of 50 mg/mL and a pH of 7.4 was incubated in PBS with D-glucose with a concentration of 100 mmol/L at 37 °C. Albumin incubated without D-glucose was used as a control. After 8 weeks of incubation, the solutions were extensively concentrated and purified. AGE-specific fluorescence (excitation, 370 nm; emission, 440 nm) was detected using ratio spectrofluorometry [26]. AGEs contained 75.20 U/mg of proteins, while control albumin contained less than 0.9 U/mg of AGEs. Both solutions contained less than 500 U/L of endotoxin, as determined by the Limulus amoebocyte lysate assay (Sigma, St. Louis, Missouri, United States).

### RNA extraction, cDNA library preparation, RNA-Seq sequencing

A total amount of 3 µg RNA from HUVECs with AGEs treatment for 0 h, 2 h, 12 h, 24 h was used for the RNA sample preparations. Sequencing libraries were generated using NEB-Next® Ultra™ RNA Library Prep Kit for Illumina® (NEB, USA). After the libraries were qualified, the different libraries were pooled and sequenced by the Illumina NovaSeq 6000. Differential expression analysis was performed using the R package DESeq2 (1.16.1) [27]. Genes with a *P* value less than 0.05 found by DESeq2 were assigned as differentially expressed genes (DEGs). Gene Ontology (GO) enrichment and Kyoto Encyclopedia of Genes and Genomes (KEGG) pathway analysis of DEGs were applied using the R package clusterProfler. Then, GO and KEGG terms with an adjusted *P* value less than 0.05 were considered significantly enriched. The sequencing data generated from this study were deposited into the Sequence Read Archive (SRA) with the accession number “PRJNA976602”.

### Cell counting kit-8 assay

Cell counting kit-8 assay (DOJINDO, Cat# CK04) was performed to measure cell proliferation activity in accordance with the manufacturer’s protocol. After 48 h of being transfected with siRNA or transduced with adenovirus, the HUVECs were trypsinized, resuspended, and then 1 × 10^4^ cells per well were seeded in a 96-well plate with AGEs or VEGFA for 24 h at 37 ℃. 10 µl CCK-8 solution was then added to each well and incubated for 1–4 h at 37 ℃. The absorbance was detected at 450 nm using a microplate reader (Thermo Fisher Scientific, USA).

### Transwell migration assay

HUVECs were transfected with siRNA or transfected with adenovirus for 48 h, followed by being trypsinized and resuspended into migration medium (ECM + 1% FBS). 1 × 10^5^ HUVECs were placed into the upper chamber of 24-well transwell insert (8 µm pore size, Corning). AGEs or VEGFA were added in the lower chamber and incubated for 24 h. Subsequently, the lower side of the transwell chamber was fixed with 4% paraformaldehyde and stained with 0.2% crystal violet in 10% ethanol for 10 min. Chambers were washed and soaked with PBS three times in order to decolorize, and then the cells on the upper chambers were wiped off. The migrated cells were photographed in five random fields under the microscope and counted with ImageJ software.

### Tube formation assay

The 96-well plate was precoated with 50 µl ice-cold Matrigel (Corning) and placed in an incubator with 37 ℃ for 30 min. After gelatinization, 3 × 10^4^ treated HUVECs were seeded into the surface of the gel and incubated for 12 h. The tubes were imaged under a microscope, and tube length or branching points were measured with ImageJ software.

### Gene promoter reporter assays

Transcriptional activity of Wnt/β-catenin signaling was evaluated through TCF/LEF response element (RE) reporter activity by using pGL4.49-TCF/LEF RE/luc2P Vector (Promega, USA). The promoter regions of the *KDR* and *HDAC9* genes were amplified from HUVECs using PCR assay (Vazyme, China, Cat# P505). The amplified fragment was cloned into a pGL3-basic vector (Promega, USA) and verified via DNA-sequencing. Then, the reporter constructs including the promoter luciferase plasmid and Renilla plasmid were co-transfected into 293T cells. A Dual-Luciferase Reporter Assay Kit (Vazyme, China, Cat# DL101) was used to measure promoter activity based on manufacturer’s protocol. Luminescence was quantified using the SpectraMax M5 Microplate Readers (Molecular Devices, USA). Data were normalized to Renilla luciferase value, which served as internal control for transfection efficiency.

### Chromatin immunoprecipitation (ChIP)-PCR

Binding sites between β-catenin/TCF and *KDR* or *HDAC9* promoters were predicted by the JASPAR database [28]. The ChIP assay was conducted using a ChIP kit (Bersinbio, China, Cat# Bes5001) according to the manufacturer’s instruction. Briefly, a total of 2 × 10^7^ HUVECs were subjected to crosslinking with 1% formaldehyde and lysed to obtain nuclear fractions, which were then sheared with sonication and precipitated with 10 μg antibody against β-catenin or control IgG. The precipitated DNA fragments were subjected to subsequent PCR amplification with the specific primer sets (Table S3 and 7) and analyzed by 3% (w/v) agarose gel electrophoresis.

### Protein–protein docking

The binding surface of VEGFR1 isoform5 and β-catenin was predicted by the Dock Proteins (ZDOCK) protocol [29]. In brief, the I-TASSER server was employed to predict and model the three-dimensional structure of VEGFR1 isoform5 and β-catenin [30], which were then submitted as receptor and ligand proteins, respectively, to ZDCOK. No amino acid was predefined as interface residue or paired interacting residue. The docking model with highest ZDOCK score was chosen for the further investigation.

### Virtual screening of inhibitor targeting VEGFR1 isoform5–β-catenin interaction

The inhibitors targeting VEGFR1 isoform5–β-catenin interaction were identified by virtual screening using AutoDock Vina. We firstly found 2 inhibitor binding pockets (pocket 3: THR3 GLN4 GLN26 SER29 TYR30 LEU31 ASP32 SER33 GLY34 HIS36 ALA305 TYR306 GLY307 GLN309 LYS312 LYS345 VAL346 VAL349; pocket 8: TRP25 LEU31 ASP32 ILE35 LYS345 GLN379 TRP383 ARG386 ASN415 THR418 CYS419) on the binding surface of VEGFR1 isoform5–β-catenin complex by using MOE-Site Finder plug-in. Then, the bindings of β-catenin pocket 3 and 8 with the small molecular compounds library of MedChemExpress (MCE) were analyzed based on molecular docking-based virtual screening via AutoDock Vina software. Virtual screening parameters were prepared by autodock tools (ADT).

### Immunofluorescent staining

HUVECs were seeded on the glass bottom dish (Cellvis, Cat# D35-10-1-N) and cultured to 70%-90% confluence following with 100 μg/mL AGEs treatment. Cells were fixed and permeabilized with 4% formaldehyde and 0.5% Triton X-100, respectively, for 10 min, followed by being blocked with 5% BSA and incubated with a primary antibody against β-catenin overnight at 4 °C and Alexa-Fluor 594-coupled secondary antibody for 1 h at room temperature. Finally, the nucleus was labeled with DAPI (1:50) and images were photographed with the laser confocal scanning microscope (Zeiss LSM780, Germany). The quantification of β-catenin location was performed as described by Feng et al. [31].

### Statistical analysis

Data were analyzed by GraphPad Prism 8.0 software (GraphPad Software, Inc.) and presented as *Mean* ± *SD*. Sample size for each study was determined in accordance with literature documentation of similar well-characterized experiments. A Student’s *t* test (two-sided) was used to determine the statistical difference between two groups. One-way ANOVA or two-way ANOVA was used to compare multiple groups with one or two independent variables, respectively, with Turkey post-hoc test. *P* value less than 0.05 was considered statistically significant.

## Results

### AGEs induce angiogenesis accompanying with pro-angiogenic genes upregulation

**Fig. 1:**
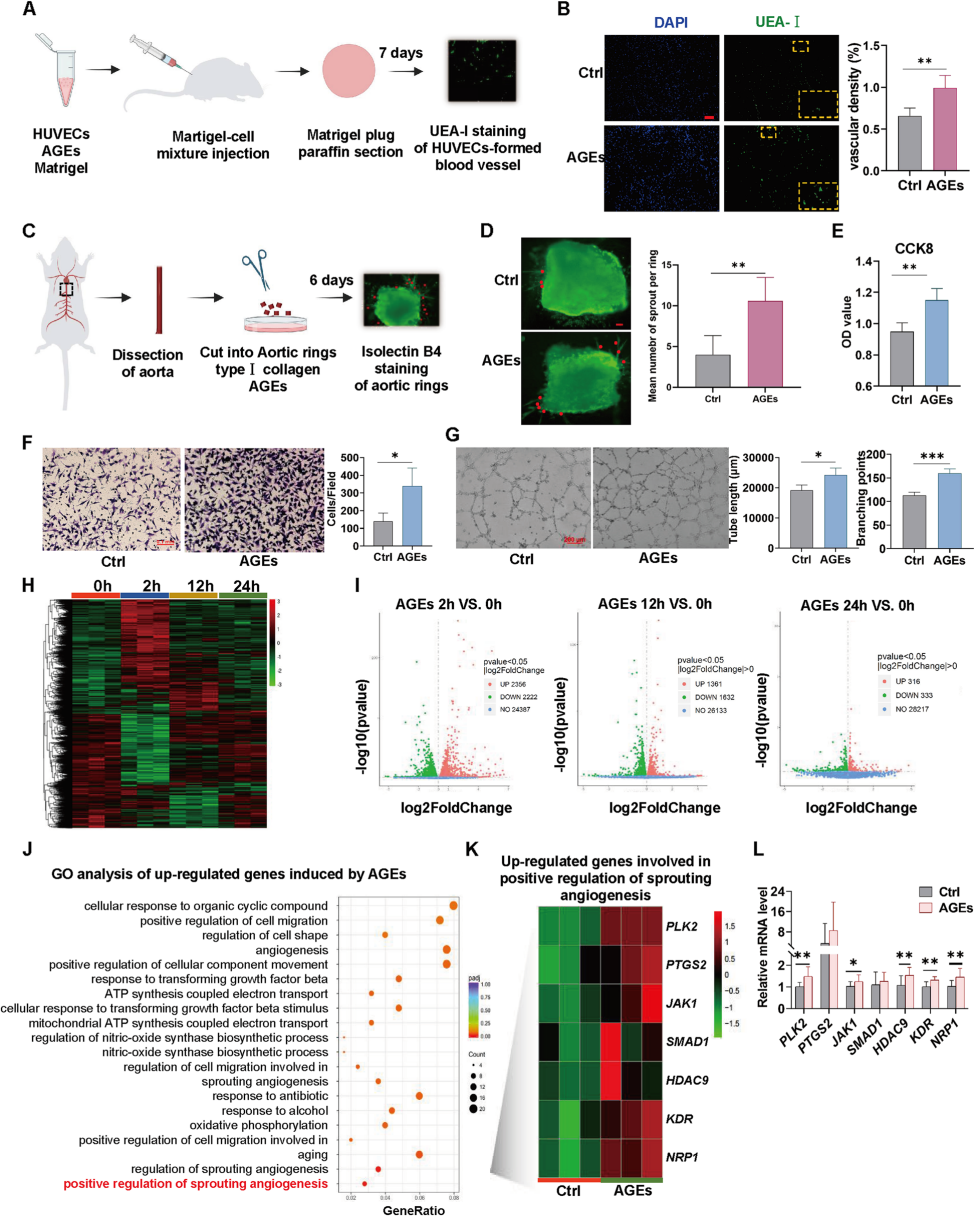
AGEs induce angiogenesis accompanying with pro-angiogenic genes upregulation. **A** Schematic diagram of the experimental design for the Matrigel plug assay. Matrigel containing HUVECs with or without AGEs (100 μg/mL) was injected subcutaneously into mouse. 7 days later, plugs were harvested and fixed with paraffin, sectioned, and stained with fluorescently labeled Ulex europaeus agglutinin I (UEA-I, Green) which binds HUVECs. **B** HUVECs-formed blood vessel in Matrigel plugs were measured by staining with UEA-I. Representative fluorescent images are shown. *n* = 4, scale bar indicates 100 μm. **C** Schematic diagram of the experimental design for the Aortic ring assay. Aorta dissected from mouse was cut into rings and then embedded in type I collagen and treated with or without AGEs (100 μg/mL). 6 days later, the rings were stained with isolectin B4 (IB4; green). **D** The mean number of sprout (indicated by red dot) from aortic ring was counted. *n* = 5, scale bar indicates 100 μm. **E** CCK8 assay was applied to evaluate the proliferation of HUVECs treated with AGEs (100 μg/mL) for 24 h. *n* = 5. **F** Transwell migration assay was conducted to evaluate the migration capacity of HUVEC treated with AGEs (100 μg/mL) for 24 h. *n* = 4, scale bar indicates 100 μm. **G** HUVECs were treated with AGEs (100 μg/mL) for 24 h and then embedded into Matrigel. The tube length and branching points were quantified using Image J. *n* = 4, scale bar indicates 200 μm. **H** Hierarchical clustering displays differential expression profiles of HUVECs treated with AGEs for 0, 2, 12 or 24 h. Columns represent individual samples and rows represent genes. Red and green indicate relatively high and low levels of gene expression, respectively, according to the saturation scale of color. *n* = 3. **I** Volcano plots show differentially expressed genes (DEGs) in the AGE-treated group for three different time points compared with the 0 h group. The blue points represent genes without significant changes, while the red and green points represent upregulated and downregulated genes respectively. *n* = 3. Threshold: DESeq2 *P* value < 0.05 |log2FoldChange|> 0. **J** Biological process terms from GO enrichment analysis of up-regulated genes in AGE-treated group compared with the Ctrl group. **K** Heatmap of upregulated genes enriched in the positive regulation of sprout angiogenesis. **L** qPCR assay was conducted to verify the mRNA expression of *PLK2*, *PTGS2*, *JAK1*, *SMAD*1, *HDAC9*, *KDR*, *NRP1* in Fig. 1K. *n* = 9 to11. Data are shown as *Mean* ± *SD*. *P < 0.05, **P < 0.01, ***P < 0.001

We first performed several experiments to confirm the pro-angiogenic effect on AGE-induced angiogenesis. The results of the Matrigel plug assay showed a significant increase in the density of vascular network formed by UEAI-lectin labeled HUVEC in the plug group with AGEs compared to the plug group without AGEs (Fig. 1A, B). Meanwhile, we obtained arterial rings from mice and stimulated them with AGEs to detect sprout level (Fig. 1C). After AGEs stimulation, the mean number of arterial ring sprout significantly increased compared to the Ctrl group (Fig. 1D). At the cellular level, the results of CCK8, transwell, and tube formation assays demonstrated that AGEs significantly increase the proliferation, migration, and tube formation activity of HUVECs (Fig. 1E-G). These findings suggest that AGEs can promote angiogenesis.

To figure out how AGEs induce angiogenesis, we did RNA-seq studies on HUVECs stimulated by AGEs for 2/12/24 h and screened 4578/2993/649 differentially expressed genes (DEGs), respectively (Fig. 1H, I). Here, a lower criterion for screening DEGs was used: *P* value < 0.05 by DESeq2 software, for the reason that too few DEGs were identified after AGEs stimulation for 24 h using a higher threshold for DEGs screening, leading to no significant results in the subsequent functional enrichment analysis. The Gene Ontology (GO) enrichment results showed that DEGs after AGEs stimulation were enriched in positive regulation of sprouting angiogenesis (Fig. 1J), which contained seven upregulated DEGs, including *PLK2* (Polo Like Kinase 2), *PTGS2* (Prostaglandin-Endoperoxide Synthase 2), *JAK1* (Janus Kinase 1), *SMAD1*, *HDAC9*, *KDR*, and *NRP1* (Neuropilin 1) (Fig. 1K). This suggests that AGEs may promote angiogenesis by upregulating these seven genes. This transcriptomic result was further confirmed by qPCR assay (Fig. 1L). However, after AGEs treatment, the expression of *PTGS2* and *SMAD1* was only slightly increased with no statistical difference (Fig. 1L). Therefore, subsequent studies focus on the remaining 5 DEGs, *PLK2*, *JAK1*, *HDAC9*, *KDR,* and *NRP1*.

### AGEs upregulate β-catenin protein level and activate its transcriptional activity

**Fig. 2:**
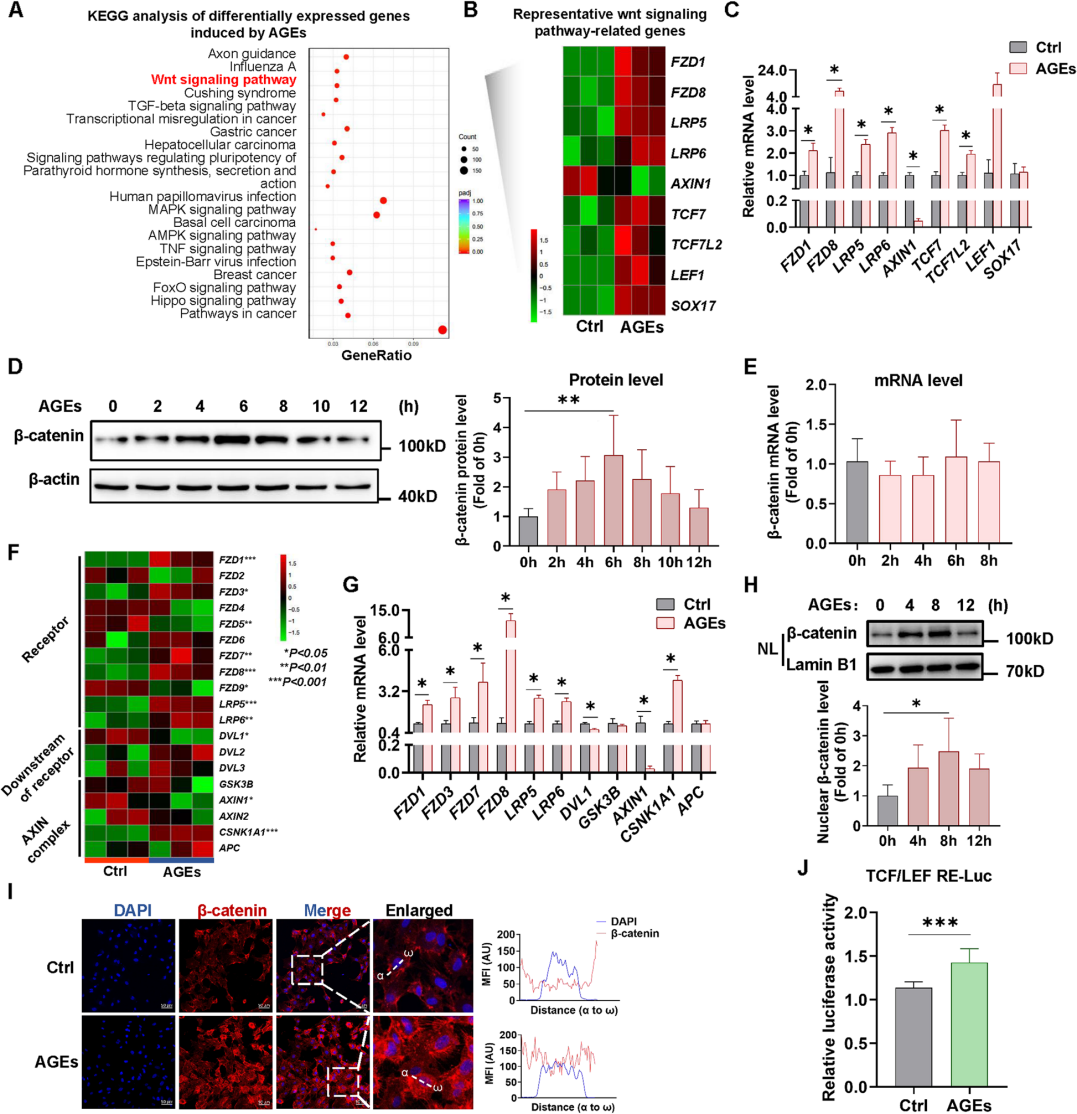
AGEs upregulate β-catenin protein level and activate its transcriptional activity. **A** The enrichment analysis of KEGG pathways of differentially expressed genes (DEGs) induced by AGEs. **B** Heatmap of Representative DEGs enriched in the Wnt signaling pathway. **C** qPCR analysis of representative DEGs enriched in Wnt signaling pathway. *n* = 4. **D** HUVECs were treated by AGEs (100 μg/mL) for different times and β-catenin protein level was measured by western blot. *n* = 5. **E** qPCR analysis of β-catenin mRNA level in HUVECs with AGEs (100 μg/mL) treatment for different times. *n* = 4. **F** Heatmap of Wnt signaling upstream genes that affect β-catenin protein level, including Wnt receptors, downstream genes of them and destruction complex genes. **G** qPCR analysis of Wnt signaling upstream genes mRNA levels in HUVECs with AGEs (100 μg/mL) treatment.* n* = 4. **H** HUVECs were treated by AGEs (100 μg/mL) for different times. Proteins of nuclear fractions were isolated and then β-catenin protein was detected by western blot. Lamin B1 was used as an internal control for nuclear proteins. *n* = 5. NL indicates the nuclear lysate. **I** HUVECs were treated by AGEs (100 μg/mL) for 8 h, and then stained for β-catenin (red). The nuclei were stained with DAPI (blue). The line charts show the mean fluorescence intensity (MFI) of the distance in the images from α to ω in arbitrary units (AU). Scale bar indicates 50 μm. **J** 293T cells were transfected with luciferase constructs containing TCF/LEF response element (RE) and cultured for 24 h followed by AGEs treatment. Dual-Luciferase Reporter Assay was performed to detect the luciferase reporter activities the and luminescence was normalized to Renilla. *n* = 8. Data are shown as *Mean* ± *SD*. *P < 0.05, **P < 0.01, ***P < 0.001

KEGG pathway enrichment result showed that the Wnt signaling pathway was one of the most significantly enriched pathways based on the corrected *P*-value, which was further validated through qPCR experiments (Fig. 2A-C). This indicates activation of the Wnt signaling pathway after stimulation of AGEs. β-catenin is a central molecule in this signaling pathway and we chose β-catenin for further investigation. We observed that the β-catenin protein level was elevated as AGEs stimulation time was prolonged, peaking at 6 h (Fig. 2D); however, there were no corresponding changes in its mRNA level (Fig. 2E). This suggests that the rise in β-catenin protein level may be attributed to a decrease in its degradation. It is known that in the classical Wnt signalling pathway, reduced degradation of β-catenin protein is due to activation of the DVL protein by the FZDs and the LRP5/6 co-receptor, which then inhibits β-catenin phosphorylation mediated by the AXIN/APC/CK1/GSK3 complex and subsequent degradation mediated by ubiquitination modifications [13]. From transcriptomic results, we also found altered expression of genes associated with β-catenin degradation following AGEs stimulation, including the upregulated Wnt receptor genes such as *FZDs* and *LRP5/6*, as well as the downregulated AXIN complex genes such as *DVL1* and *AXIN1* (Fig. 2F, G). Nuclear protein isolation and immunofluorescence assays revealed the increased translocation of β-catenin into the nucleus after AGEs treatment (Fig. 2H, I). The luciferase reporter assay presented a rise in β-catenin-TCF/LEF response element-luciferase activity after AGEs stimulation (Fig. 2J), suggesting that AGEs enhance the transcriptional activity of β-catenin. Taken together, these results indicate that AGEs can initiate the Wnt signaling pathway, upregulate β-catenin protein level and activate its transcriptional activity.

### β-catenin transcriptional activity is involved in AGE-induced angiogenesis via upregulating *HDAC9/KDR*

**Fig. 3:**
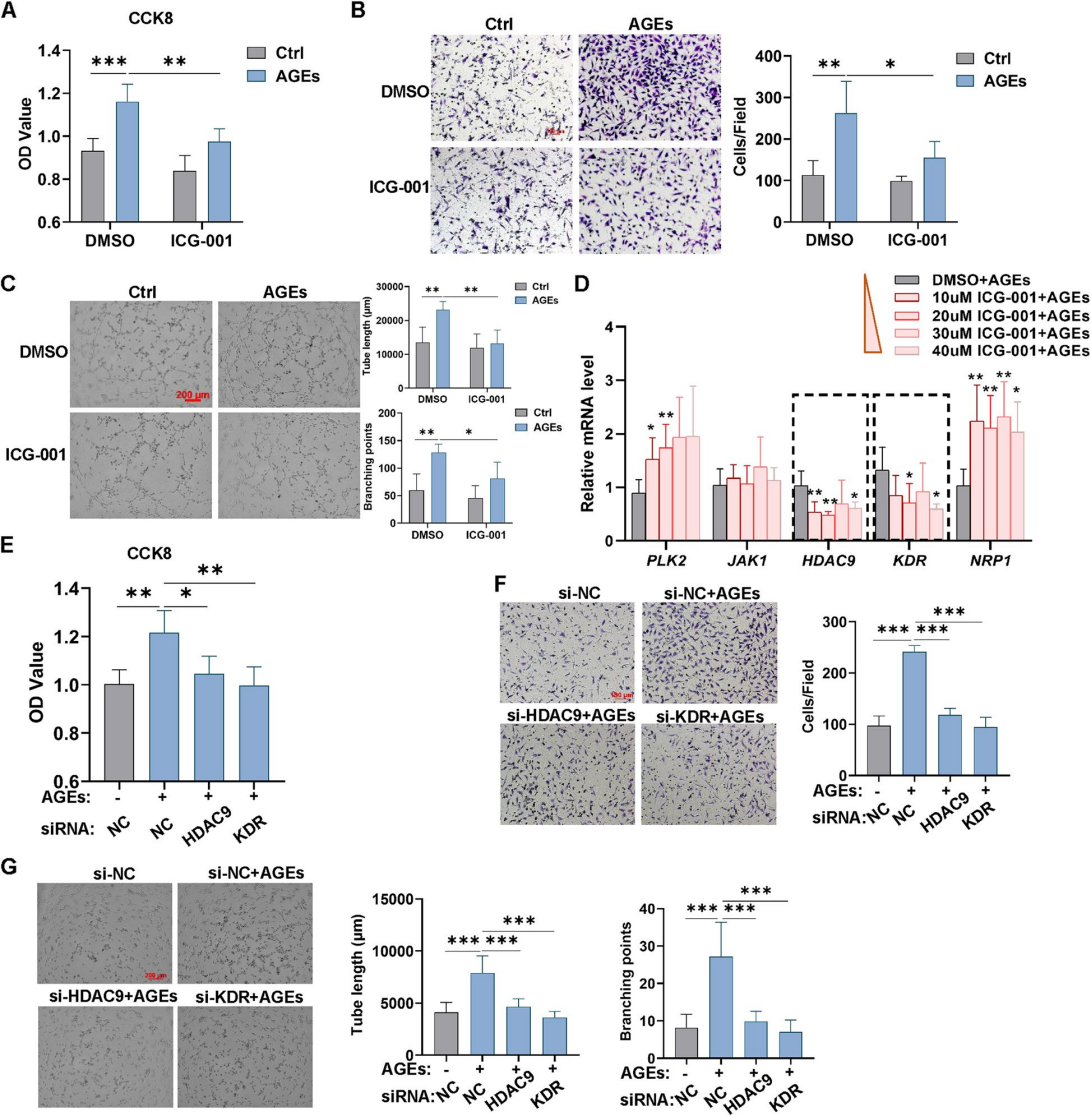
β-catenin transcriptional activity is involved in AGE-induced angiogenesis via upregulating *HDAC9*/*KDR*. **A**-**C** HUVECs were treated by AGEs (100 μg/mL) for 24 h in the presence of 20 μM ICG-001, an inhibitor that antagonizes β-catenin/TCF-mediated transcription, and then the CCK8 assay (**A**), Transwell assay (**B**), and tube formation assay (**C**) were perform to evaluate the proliferation, migrated cell number and tube length, branching points, respectively. *n* = 4 to 5, scale bar indicates 100 or 200 μm. **D** HUVECs were treated with AGEs (100 μg/mL) for 24 h in the presence of ICG-001 with different doses and then the mRNA levels of *PLK2*, *JAK1*, *HDAC9*, *KDR*, *NRP1* were detected by qPCR. *n* = 5 to 7. **E**–**G** Effects of HDAC9 and KDR on AGE-induced angiogenesis. After transfection with negative control (NC) siRNA or with specific siRNA targeting HDAC9 or KDR for 48 h, HUVECs were treated with AGEs (100 μg/ml) for 24 h followed by the CCK8 assay (**E**), Transwell assay (**F**), and tube formation assay (**G**) to evaluate the OD value, migrated cell number and tube length, branching points respectively. *n* = 5 to 6, scale bar indicates 100 or 200 μm. Data are shown as *Mean* ± *SD*. *P < 0.05, **P < 0.01, ***P < 0.001

Next, we found that after downregulating β-catenin expression with the corresponding siRNA, the increased levels of proliferation, migration, and tubulogenesis caused by AGEs could be attenuated (Fig. S1A-C; left panel of Fig. S9A), suggesting that β-catenin takes part in AGE-mediated angiogenesis. As a signal transduction mediator, β-catenin exerts its transcriptional activity by regulating Wnt/β-catenin target genes expression. We next verified whether AGEs mediate angiogenesis through β-catenin transcriptional activity. ICG-001 is a low-molecular-weight inhibitor that selectively antagonizes β-catenin/TCF-initiated transcription. We first inhibited β-catenin transcriptional activity with ICG-001 followed by AGEs exposure and then detected the angiogenic level of HUVECs. The results showed that ICG-001 alleviated AGE-induced increases in endothelial cell proliferation, migration, and tubulogenesis (Fig. 3A-C), indicating that β-catenin transcriptional activity participates in AGE-induced angiogenesis.

As mentioned above, GO enrichment of the transcriptomic analysis results uncovers the positive regulation of sprouting angiogenesis, whose DEGs containing *PLK2*, *JAK1*, *HDAC9*, *KDR*, and *NRP1* were verified to be significantly upregulated in response to AGEs stimulation (Fig. 1J-L). We wondered whether these five genes act as Wnt/β-catenin target genes participated in AGE-induced angiogenesis. We used different concentrations of ICG-001 to inhibit the transcriptional activity of β-catenin upon AGEs treatment. qPCR assay was conducted to detect the mRNA levels of these five genes, and the results showed that only the mRNA levels of *HDAC9* and *KDR* were reduced (Fig. 3D), suggesting that *HDAC9* and *KDR* may be among the target genes regulated by β-catenin under AGEs treatment.

Meanwhile, we found that after downregulating *HDAC9* or *KDR* expression with the corresponding siRNA, the increased levels of proliferation, migration, and tubulogenesis caused by AGEs could be attenuated (Fig. 3E-G; S9B-C). All these evidences imply that *HDAC9* and *KDR* potentially act as a Wnt/β-catenin target gene to participate in AGE-induced angiogenesis and we proceeded with separate validation analyses for these two genes.

### β-catenin increases the expression of *KDR* and *HDAC9* by upregulating NANOG and POU5F1, respectively, under AGEs treatment

**Fig. 4:**
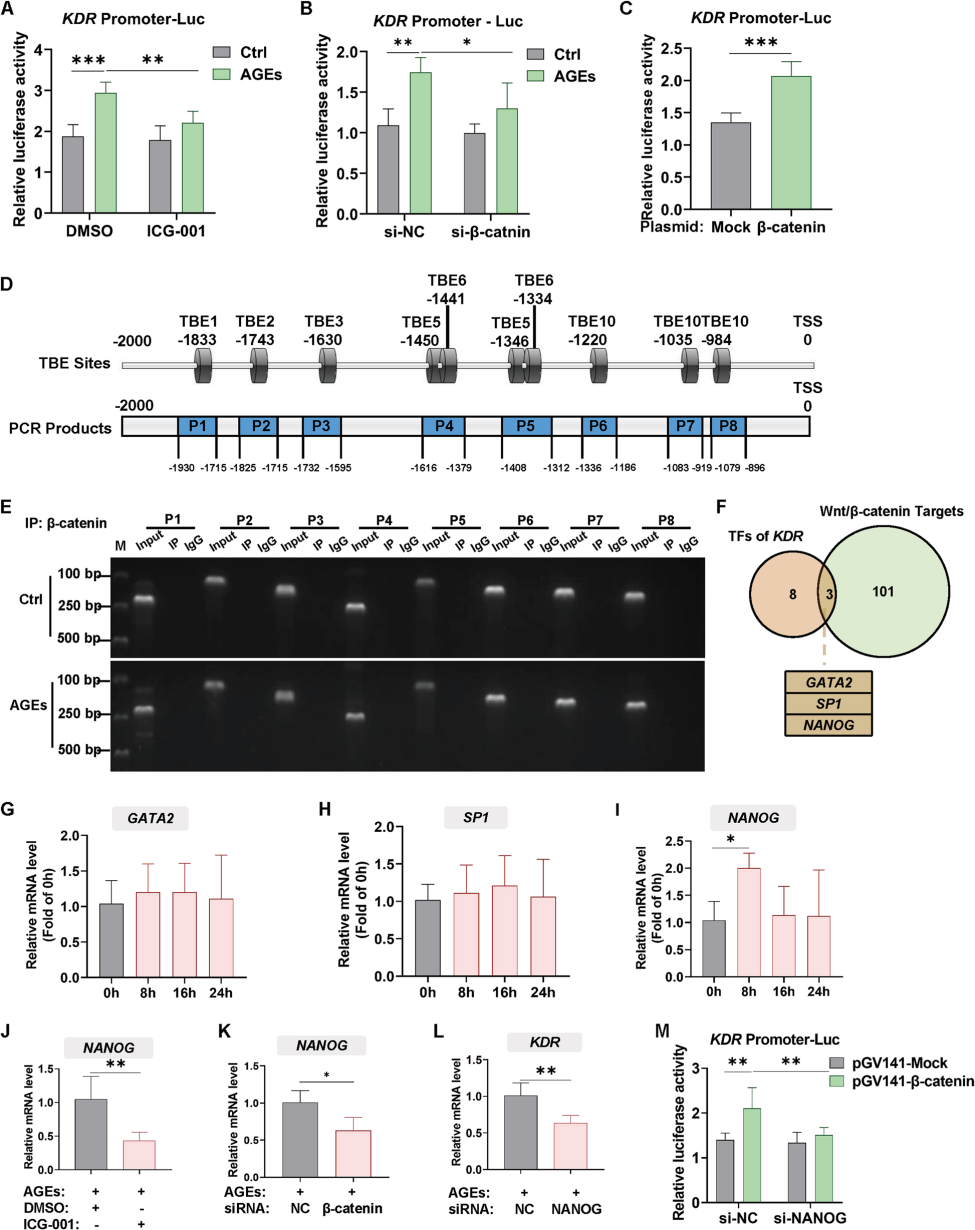
β-catenin increases the expression of *KDR* by upregulating NANOG under AGEs treatment. **A**, **B** 293T cells were transfected with *KDR* promoter luciferase construct followed by AGEs stimulation in the presence or absence of 20 μM ICG-001 for 24 h (**A**) or β-catenin siRNA (**B**). The Dual-Luciferase Reporter Assay was used to detect promoter activity, and luminescence was normalized to Renilla. *n* = 5. **C** 293T cells were co-transfected with *KDR* promoter luciferase construct and β-catenin-overexpression plasmid. After 24 h. The Dual-Luciferase Reporter Assay was performed to detect promoter activity, and luminescence was normalized to Renilla. *n* = 5. **D** Schematic diagram of the predicted binding region of β-catenin to the *KDR* promoter DNA sequence (upper panel) and the PCR amplification product location of 8 primers for the predicted binding element (lower panel). IBS software was used as a drawing tool [32]. **E** The binding of β‐catenin to the *KDR* promoter in response to AGEs treatment was determined in HUVECs by ChIP assay and quantified by PCR. Experiments were repeated three times with similar results and one representative result is shown. **F** Venn diagram showing overlapping genes between transcriptional factors of *KDR* and Wnt/β-catenin target genes. **G**-**I** HUVECs were treated with AGEs (100 μg/ml) for different times and the mRNA levels of GATA2/SP1/NANOG were detected by qPCR. *n* = 5 to 7. **J**, **K** HUVECs were pretreated with ICG-001 (20 μM) (**J**) or β-catenin siRNA (**K**) followed by AGEs (100 μg/ml) stimulation and NONOG mRNA levels were detected by qPCR.* n* = 5. **L** siRNA targeting NANOG was transfected into HUVECs followed by AGEs (100 μg/ml) treatment and *KDR* mRNA levels were detected by qPCR, respectively. *n* = 4. **M** 293T cells were co-transfected with *KDR* promoter luciferase construct, β-catenin-overexpression plasmid and NONOG siRNA. After 48 h, the Dual-Luciferase Reporter Assay was conducted to detect *KDR* promoter activity, and luminescence was normalized to Renilla. *n* = 6. Data are shown as *Mean* ± *SD*. *P < 0.05, **P < 0.01, ***P < 0.001

Firstly, to investigate whether *KDR* is a target gene of β-catenin, we constructed a reporter gene plasmid containing the *KDR* promoter region-luciferase, which was transferred to 293T cells followed by inhibition of β-catenin transcriptional activity via ICG-001 with or without AGEs treatment. The results showed that AGEs significantly upregulated *KDR* promoter reporter luciferase activity, while this effect was significantly reversed after inhibition of β-catenin transcriptional activity or downregulating β-catenin with siRNA (Fig. 4A-B; right panel of Fig. S9A). After overexpression of β-catenin, *KDR* promoter reporter luciferase activity was significantly up-regulated (Fig. 4C). We next used the JASPAR database to predict the binding sites of the β-catenin/TCF and *KDR* promoter regions and identified 10 β-catenin/TCF binding elements (TBEs) where β-catenin/TCF binds with *KDR* promoters (Table S2; upper panel of Fig. 4D). Then ChIP-PCR experiment was performed to verify which region β-catenin/TCF binds to. We designed specific primers whose PCR amplification product contains the TBE sequence and ranges from 70 to 300 bp in length (Table S3; lower panel of Fig. 4D). However, we did not detect appreciable binding of β-catenin to the predicted TBEs, indicating that there was no interaction between β-catenin and the predicted binding element of the *KDR* promoter (Fig. 4E). These results suggest that β-catenin/TCF may not function as a direct transcription factor for *KDR*, but rather regulate *KDR* expression through an indirect mechanism. We sought this indirect mechanism by identifying genes that act as transcription factors for *KDR*, which are also among the target genes of β-catenin. We retrieved 11 reported transcription factors regulating *KDR* expression and 104 target genes of β-catenin from public databases (Tables S4; 5). After intersecting the two datasets, three candidates were screened: *GATA2*, *SP1*, and *NANOG* (Fig. 4F). We first examined the expression levels of these three genes, and the results showed that only *NANOG* expression level increased after AGEs stimulation for 8 h, while *GATA2* and *SP1* levels remained unaltered (Fig. 4G-I). Therefore, we selected *NANOG* for further analysis. Meanwhile, *NANOG* expression was also decreased upon the inhibition of β-catenin transcriptional activity using ICG-001 or downregulating β-catenin with siRNA (Fig. 4J-K; left panel of Fig. S9A). *NANOG* knockdown mediated by siRNA significantly reduced the expression level of *KDR* in the presence of AGEs (Fig. 4L; left panel of Fig. S9D) and the β-catenin-induced *KDR* promoter reporter activity enhancement (Fig. 4M; right panel of Fig. S9D). Collectively, the above results suggest that β-catenin could indirectly upregulate *KDR* under AGEs treatment by enhancing the expression of the transcription factor NANOG.

Next, we focused on another gene, *HDAC9*, to explore if it is a target gene of β-catenin. Similarly, we constructed a reporter gene plasmid containing the *HDAC9* promoter region (- 2000 bp to + 100 bp)-luciferase. The results showed that inhibiting β-catenin transcriptional activity with ICG-001 or downregulating β-catenin with siRNA significantly reversed AGE-upregulated *HDAC9* promoter reporter luciferase activity (Fig. S2A-B; right panel of Fig. S9A), while overexpression of β-catenin significantly enhanced *HDAC9* promoter reporter luciferase activity (Fig. S2C). 11 predicted TBEs of the β-catenin and *HDAC9* promoter regions were obtained from JASPAR database (Table S6; upper panel of Fig. S2D), and specific primers were designed for verification (Table S7; lower panel of Fig. S2D). Similar to the *KDR* promoter region-luciferase activity results, we observed no significant binding of β-catenin to the predicted TBEs with or without AGEs stimulation (Fig. S2E). Using similar approaches, 14 transcription factors that regulate *HDAC9* expression were found and then cross-tabulated with the dataset of 104 β-catenin targeted genes, and finally three genes, *GATA2*, *SP1*, and *POU5F1* were identified (Table S8; Fig. S2F). The expression levels of *GATA2* and *SP1* had been proved to remain unaltered under AGEs treatment (Fig. 4G, H). The expression level of *POU5F1* increased after AGEs stimulation for 8 h (Fig. S2G). Inhibition of β-catenin transcriptional activity with ICG-001 or downregulating β-catenin with siRNA under AGEs treatment resulted in downregulation of POU5F1 expression level (Fig. S2H-I; left panel of Fig. S9A). *POU5F1* knockdown mediated by siRNA significantly decreased the expression level of *HDAC9* in the presence of AGEs (Fig. S2J; left panel of Fig. S9E) and the β-catenin-induced *HDAC9* promoter reporter activity enhancement (Fig. S2K; right panel of Fig. S9E). Taken together, these findings suggest that β-catenin may indirectly regulate *HDAC9* by upregulating the expression of transcription factor POU5F1 under AGEs treatment.

### AGEs mediate β-catenin phosphorylation and Y142 is the key site of phosphorylation

**Fig. 5:**
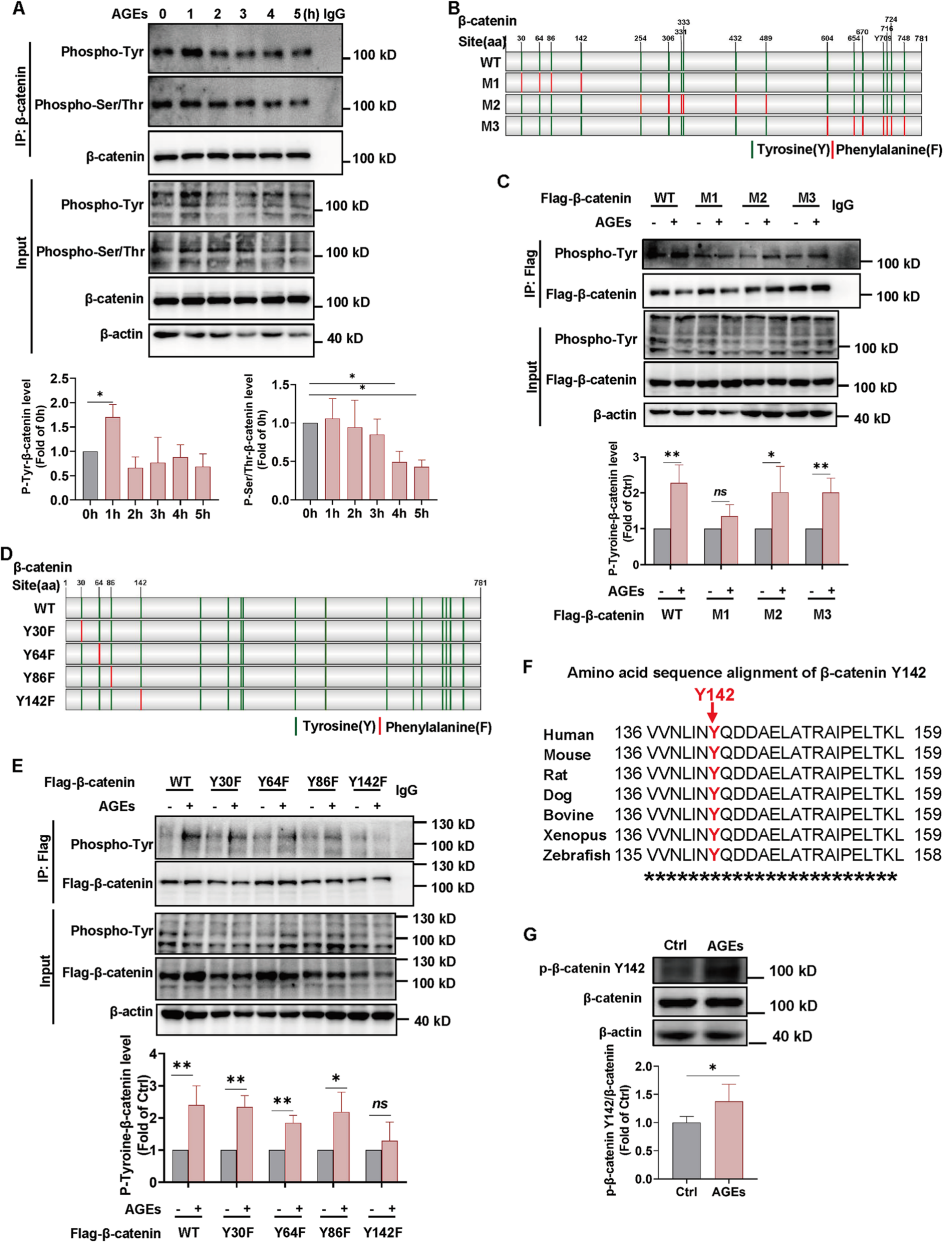
AGEs mediate β-catenin phosphorylation and Y142 is the key site of phosphorylation. **A** HUVECs were treated with AGEs (100 μg/ml) for different times, and then serine/threonine phosphorylation and tyrosine phosphorylation level of β-catenin were detected by western blot. One representative cropped blot of three independent experiments is shown. **B** Schematic diagram of constructing the three types of β-catenin multi-site mutant (Y to F) plasmids. Vertical line in green means tyrosine (Y) while line in red means the site is mutated to phenylalanine (F). Illustrated by IBS software. **C** β-catenin multi-site mutant (Y to F) plasmid was transfected into 293T cells followed by AGEs treatment, and then tyrosine phosphorylation level of β-catenin was detected by western blot. *n* = 4. One representative cropped blot of four independent experiments is shown and the phosphorylation protein level is calculated relative to each group without AGEs treatment, defined as “1”. **D** Schematic diagram of constructing the four types of β-catenin single-site mutants (Y to F) plasmids. Vertical line in green means tyrosine (Y) while line in red means the site is mutated to phenylalanine (F). Illustrated by IBS software. **E** The β-catenin single-site mutant (Y to F) plasmid was transfected into 293T cells followed by AGEs treatment, and then tyrosine phosphorylation level of β-catenin was detected by western blot. *n* = 4. One representative cropped blot of four independent experiments is shown and the phosphorylation protein level is calculated relative to each group without AGEs treatment, defined as “1”. **F** Amino acid sequence alignment of β-catenin Y142 residues among β-catenin homologs from different species. Y142 residue in various species is colored in red. **G** HUVECs were treated with AGEs (100 μg/ml) for 1 h and then phosphorylation of β-catenin Y142 was measured by western blot. *n* = 5. Data are shown as *Mean* ± *SD*. *P < 0.05, **P < 0.01, ***P < 0.001

It is well established that β-catenin phosphorylation plays a key role in its transcriptional activity [33]. We next focused on the β-catenin phosphorylation. We detected an increase in β-catenin tyrosine phosphorylation and a decrease in β-catenin serine/threonine phosphorylation within 5 h of AGEs stimulation (Fig. 5A). It is well documented that the decrease in serine/threonine phosphorylation correlates with the increase in β-catenin protein level, which is consistent with our results above (Fig. 2D). However, tyrosine phosphorylation of β-catenin is relatively less well known by researchers. Thus, we chose to follow up with a study of tyrosine phosphorylation of β-catenin, which contains 17 tyrosine phosphorylation modification sites (Fig. S3). In order to screen the key β-catenin tyrosine phosphorylation sites after AGEs treatment, we first constructed three β-catenin multi-sites tyrosine phospho-deficient mutants (Tyrosine → Phenylalanine, Y → F), namely M1, M2, and M3 (Fig. 5B, S4A). M1 contains 4 mutated sites (Y30F/Y64F/Y86F/Y142F), M2 contains 6 mutated sites (Y254F/Y306F/Y331F/Y333F/Y432F/Y489F), and M3 contains 7 mutated sites (Y604F/Y654F/Y670F/Y709F/ Y716F/Y724F/Y748F). We transfected these three multi-site tyrosine phospho-deficient plasmids into 293T cells respectively and examined β-catenin tyrosine phosphorylation level after AGEs stimulation. The results showed that after AGEs treatment, the β-catenin tyrosine phosphorylation level in the M1 group increased, but the magnitude of this increase was lower compared to the WT, M2 or M3 groups (Fig. 5C), suggesting that the key tyrosine phosphorylation sites of β-catenin following AGEs stimulation may mainly occur within the four mutated sites of M1, including Y30, Y64, Y86, and Y142.

We then constructed single-site phospho-deficient plasmids for these four sites (Fig. 5D, S4B) and transfected them into 293T cells, respectively, followed by AGEs exposure. The results showed that the β-catenin tyrosine phosphorylation level in the Y142F mutant plasmid group increased less than that in the other groups after AGEs stimulation (Fig. 5E). This result suggests that the key tyrosine phosphorylation site of β-catenin may occur mainly at the Y142 site upon stimulation by AGEs. Furthermore, amino acid sequence alignment result showed that the β-catenin Y142 site was highly conserved across multiple species (Fig. 5F). Using an antibody specific for β-catenin Y142 phosphorylation, we found that the AGEs stimulation significantly enhanced β-catenin Y142 phosphorylation level (Fig. 5G). These evidences suggest that Y142 is a key site for phosphorylation of β-catenin upon stimulation by AGEs.

### β-catenin Y142 phosphorylation accounts for AGE-induced β-catenin transcriptional activity and angiogenesis

**Fig. 6:**
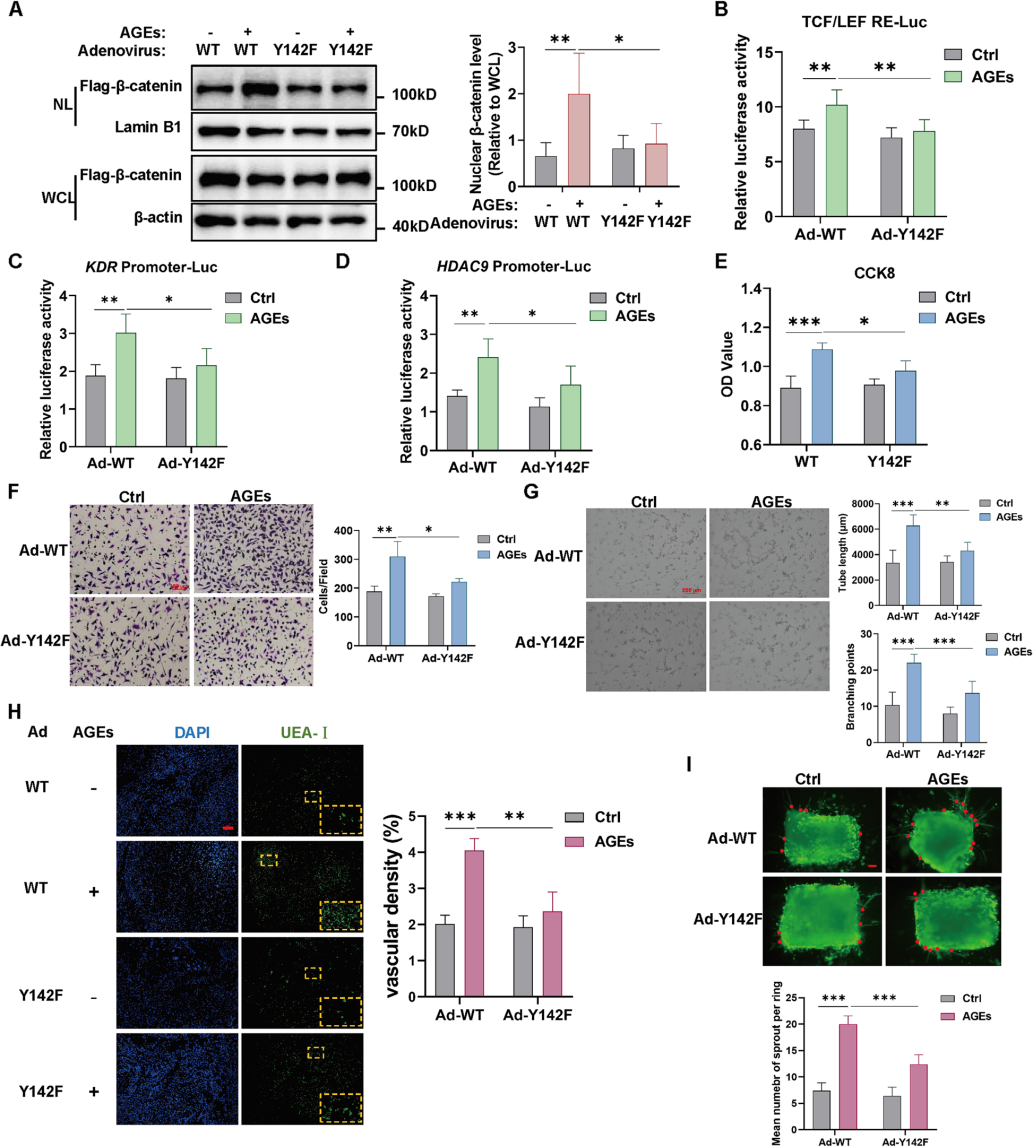
β-catenin Y142 phosphorylation accounts for AGE-induced β-catenin transcriptional activity and angiogenesis. **A** After transfection with β-catenin WT or Y142F-overexpression adenovirus for 48 h, HUVECs were stimulated by AGEs (100 μg/mL) for 8 h. Proteins of the nuclear and whole cell fractions were isolated and then β-catenin protein level was detected by western blot, respectively. *n* = 6. NL indicates nuclear lysate; WCL indicates whole cell lysate. **B**-**D** 293T cells were co-transfected with β-catenin WT or Y142F-overexpression adenovirus and TCF/LEF response element (RE) (**B**), *KDR* promoter (**C**) or *HDAC9* promoter (**D**) luciferase constructs followed by AGEs stimulation (100 μg/ml). The Dual-Luciferase Reporter Assay was conducted to detected luciferase reporter activities, and luminescence was normalized to Renilla. *n* = 5–6. **E**–**G** After transfection with β-catenin WT or Y142F-overexpression adenovirus for 48 h, HUVECs were stimulated with AGEs (100 μg/ml) for 24 h followed by the CCK8 assay (**E**), Transwell assay (**F**), and tube formation assay (**G**) to evaluate the OD value, migrated cells number and tube length, branching points, respectively. *n* = 3 to 6, scale bar indicates 100 or 200 μm. **H** Matrigel containing HUVEC transfected Ad-WT or Ad-Y142F in the presence or absence of AGEs (100 μg/mL) treatment was injected subcutaneously into mouse. A week later, plugs were harvested. Ulex europaeus agglutinin I (UEAI, Red) was used to stain HUVECs and HUVECs-formed blood vessels were measured. *n* = 3, scale bar indicates 100 μm. Ad indicates adenovirus overexpressing the corresponding protein. **I** Aortic rings were transfected with Ad-WT or Ad-Y142F, treated with or without AGEs (100 μg/mL) for 6 days and then stained with isolectin B4 (IB4; green). The mean number of sprout (indicated by red dot) from aortic ring was counted. n = 5, scale bar indicates 100 μm. Data are shown as *Mean* ± *SD*. *P < 0.05, **P < 0.01, ***P < 0.001

Next, we further explored the function of β-catenin Y142 phosphorylation in the process of AGE-induced angiogenesis. After confirming the transfection efficiency of β-catenin Y142 phospho-deficient adenovirus (Fig. S9F), we first verified if β-catenin Y142 phosphorylation affects its nuclear translocation and transcriptional activity. The result showed that the increased level of β-catenin translocation into the nucleus mediated by AGEs was alleviated after transfection with the β-catenin Y142 phospho-deficient adenovirus (Fig. 6A). The luciferase reporter gene result also showed a reduction of AGE-enhanced β-catenin/TCF transcriptional activity, *KDR* and *HDAC9* promoter reporter luciferase activity. (Fig. 6B-D). Meanwhile, the application of β-catenin Y142 phospho-deficient adenovirus resulted in a significantly reduced pro-angiogenic effect of AGEs treatment in HUVECs (Fig. 6E-G), as well as in Matrigel plug and arterial ring (Fig. 6H-I). The above results suggest that blocking β-catenin Y142 phosphorylation attenuates *KDR* and *HDAC9* expression and the pro-angiogenic effect of AGEs.

Additionally, we investigated whether the effects of β-catenin 142 phosphorylation on angiogenesis are of general relevance by using a broader and more established model of VEGF-induced angiogenesis. Cell proliferation, migration, and tube formation assays showed that β-catenin Y142 phospho-deficient adenovirus resulted in significantly reduced levels of proliferation, migration, and tube formation compared to the β-catenin WT adenovirus upon stimulation by VEGF (Fig. S5A-C). These findings imply that β-catenin Y142 phosphorylation participates in VEGF-induced angiogenesis and that its effect on angiogenesis has some general significance to some extent.

### VEGFR1 isoform 5 accounts for β-catenin Y142 phosphorylation

**Fig. 7:**
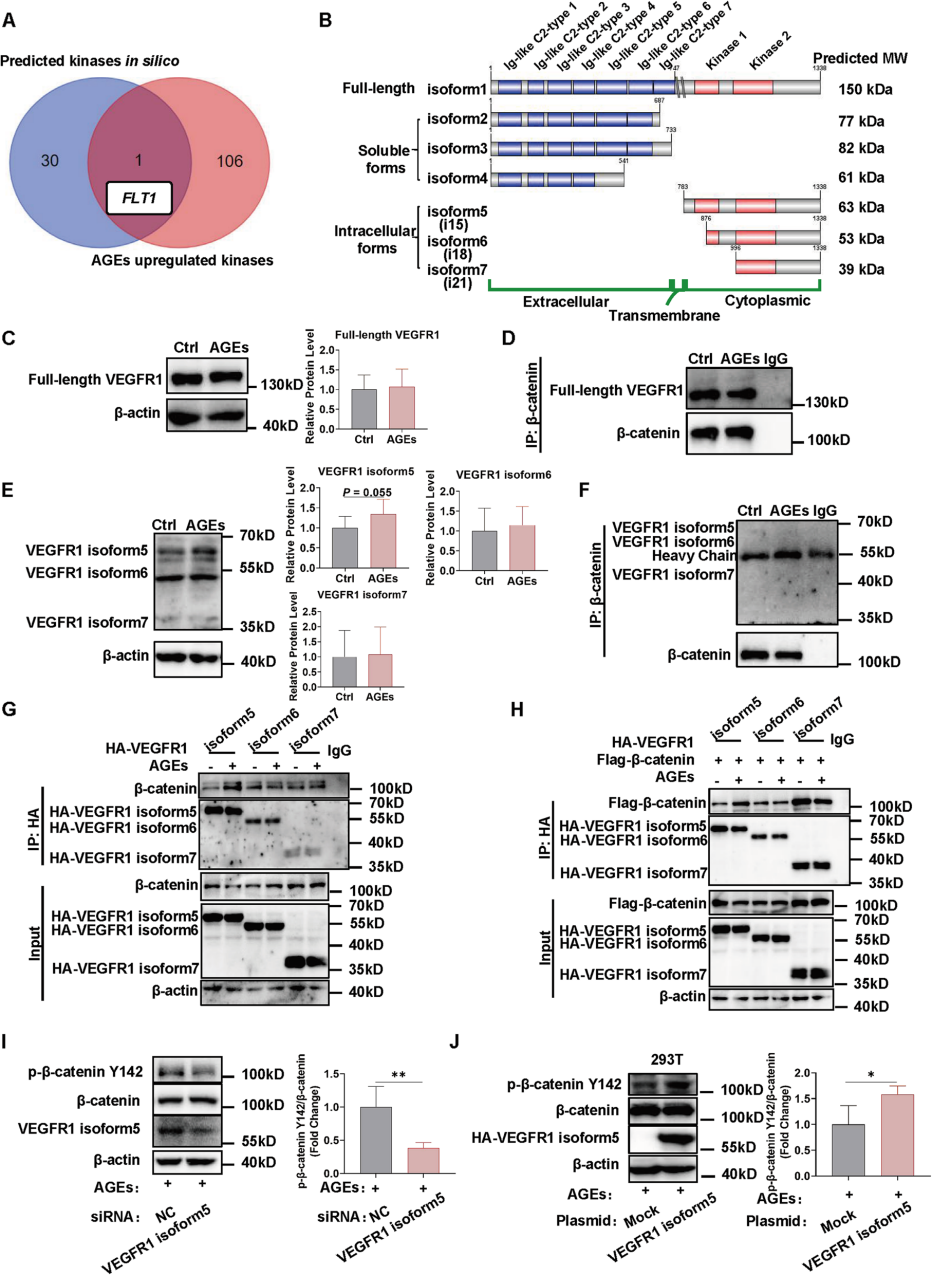
VEGFR1 isoform 5 accounts for β-catenin Y142 phosphorylation. **A** Venn diagram showing overlapping genes between the kinases of β-catenin Y142 predicted in silico from the GPS database [34] and AGE-upregulated kinases from DEGs of transcriptomic. **B** Schematic diagram of the 7 isoforms of VEGFR1. Detailed information was obtained from the Uniprot database. **C** HUVECs were stimulated by AGEs (100 μg/mL) for 1 h, and the full-length VEGFR1 levels were measured by western blot. *n* = 5. **D** HUVECs were treated with AGEs (100 μg/mL) for 1 h and then Co-IP assay was performed with β-catenin antibody to analyze the interaction between β-catenin and the full-length VEGFR1. Experiments were repeated three times with similar results and one representative result is shown. **E** HUVECs were stimulated by AGEs (100 μg/mL) for 1 h, and the VEGFR1 isoform5, isoform6 and isoform7 protein levels were measured by western blot. *n* = 8. **F** HUVECs were stimulated by AGEs (100 μg/mL) for 1 h and then Co-IP assay was performed with β-catenin antibody to analyze the interaction between β-catenin and isoform5/6/7. Experiments were repeated six times with similar results and one representative result is shown. **G** The HA-tagged VEGFR1 isoform5, isoform6 or isoform7 plasmid was transfected into 293T cells followed by AGEs treatment for 1 h, and then Co-IP assay was performed with HA antibody to analyze the interaction between endogenic β-catenin and ectogenic VEGFR1 isoform5/6/7. Experiments were repeated three times with similar results and one representative result is shown. **H** The Flag-tagged β-catenin-overexpressing plasmid was co-transfected with HA-tagged VEGFR1 isoform5, isoform6 or isoform7 plasmid into 293T cells followed by AGEs treatment for 1 h, and then Co-IP assay was performed with HA antibody to analyze the interaction between ectogenic β-catenin and these three isoforms of VEGFR1. Experiments were repeated three times with similar results and one representative result is shown. **I** HUVECs were transfected with siRNA targeting VEGFR1 isoform5 followed by AGEs (100 μg/mL) for 1 h and the phosphorylation level of β-catenin Y142 was quantified by western blot. *n* = 5. **J** 293T cells were transfected with VEGFR1 isoform5-overexpression plasmid followed by AGEs (100 μg/mL) for 1 h and the phosphorylation level of β-catenin Y142 was quantified by western blot. *n* = 5. Data are shown as *Mean* ± *SD*. *P < 0.05, **P < 0.01, ***P < 0.001

Next, we focused on the kinases that regulate β-catenin Y142 tyrosine phosphorylation under AGEs stimulation. We identified one candidate gene, *FLT1* (Fms-Like Tyrosine Kinase 1), by intersecting two datasets obtained from the kinases predicted by the GPS database and the kinases whose expression was upregulated after AGEs stimulation in transcriptomic results, respectively (Fig. 7A; Table S9). The *FLT1* gene encodes VEGFR1, a protein kinase-coupled receptor with seven isoforms, of which the full-length VEGFR1 (VEGFR1 isoform1) consists of seven structural domains in the extracellular segment, one domain in the transmembrane region, and two kinase domains in the intracellular segment (Fig. 7B). We concentrated on the full-length isoform and the three intracellular isoforms, each of which contains kinase structural domains. Protein levels of the full-length and three intracellular isoforms of VEGFR1 were measured by western blot after AGEs exposure. The antibody epitope is located at aa1016-1065 and recognizes both intracellular and full-length types of VEGFR1.The results showed that the protein levels of full-length VEGFR1 and VEGFR1 isoform6 and 7 did not change after AGEs stimulation, while VEGFR1 isoform5 was upregulated (Fig. 7C; E). We also designed two pairs of qPCR primers to detect the expression of full-length isoform and intracellular isoforms of VEGFR1, respectively. The PCR amplified products of primer 1 (P1) reflect the expression of the full-length VEGFR1, while the PCR amplified products of primer 2 (P2) reflect the expression of both the full-length and intracellular isoforms (Fig. S6A; Table S12). The results showed that the level of P1 amplified products did not alter after AGEs stimulation (Fig. S6B), while the level of P2 amplified product was significantly upregulated (Fig. S6C). These results indicated that intracellular VEGFR1 isoform expression was upregulated while full-length VEGFR1 expression was not significantly changed, which was consistent with the western blot results (Fig. 7C; E).

Meanwhile, we used co-immunoprecipitation experiment to detect the interaction between the full-length isoform or intracellular VEGFR1 isoforms and β-catenin. We first detected the interaction between full-length VEGFR1 and β-catenin. The results showed that there was no increase in their interaction level following AGEs treatment (Fig. 7D), indicating that AGEs may not upregulate β-catenin Y142 phosphorylation level via full-length VEGFR1. We then detected the interaction between intracellular VEGFR1 isoforms and β-catenin, but failed to detect the signal of endogenic intracellular VEGFR1 isoforms, most likely due to the signal interference from the Co-IP antibody heavy chain (Fig. 7F). Therefore, we then constructed three plasmids overexpressing ectogenic intracellular VEGFR1 isoforms (isoform5, 6, 7)-HA fusion proteins and transferred them into 293T cells, respectively, to detect the interaction between endogenic β-catenin and ectogenic VEGFR1 intracellular isoforms. The results showed that after AGEs treatment, the interaction between β-catenin and VEGFR1 isoform5 was enhanced, while there was no significant change between β-catenin and VEGFR1 isoform6 or 7 (Fig. 7G). Meanwhile, after co-transfection of ectogenic Flag-tagged β-catenin and HA-tagged VEGFR1 intracellular isoforms plasmids, similar to the above results, AGEs stimulation increased the interaction level between ectogenic β-catenin and ectogenic VEGFR1 isoform5 but not isoform6 or 7 (Fig. 7H). These results suggest that the upstream kinase for β-catenin Y142 phosphorylation may be VEGFR1 isoform5 under AGEs treatment. Further validation showed that after downregulating the expression of VEGFR1 isoform5 in HUVECs with siRNA targeting VEGFR1 isoform5 without affecting the expression of other isoforms (Fig. S9G), the phosphorylation level of β-catenin Y142 decreased under AGEs exposure (Fig. 7I), while after overexpressing VEGFR1-isoform5 protein, the β-catenin Y142 phosphorylation level increased (Fig. 7J), leading us to infer that VEGFR1 isoform5 mediates β-catenin Y142 phosphorylation.

### Bioymifi acts as an inhibitor of VEGFR1 isoform5–β-catenin interaction and blocks AGE-induced angiogenesis

**Fig. 8:**
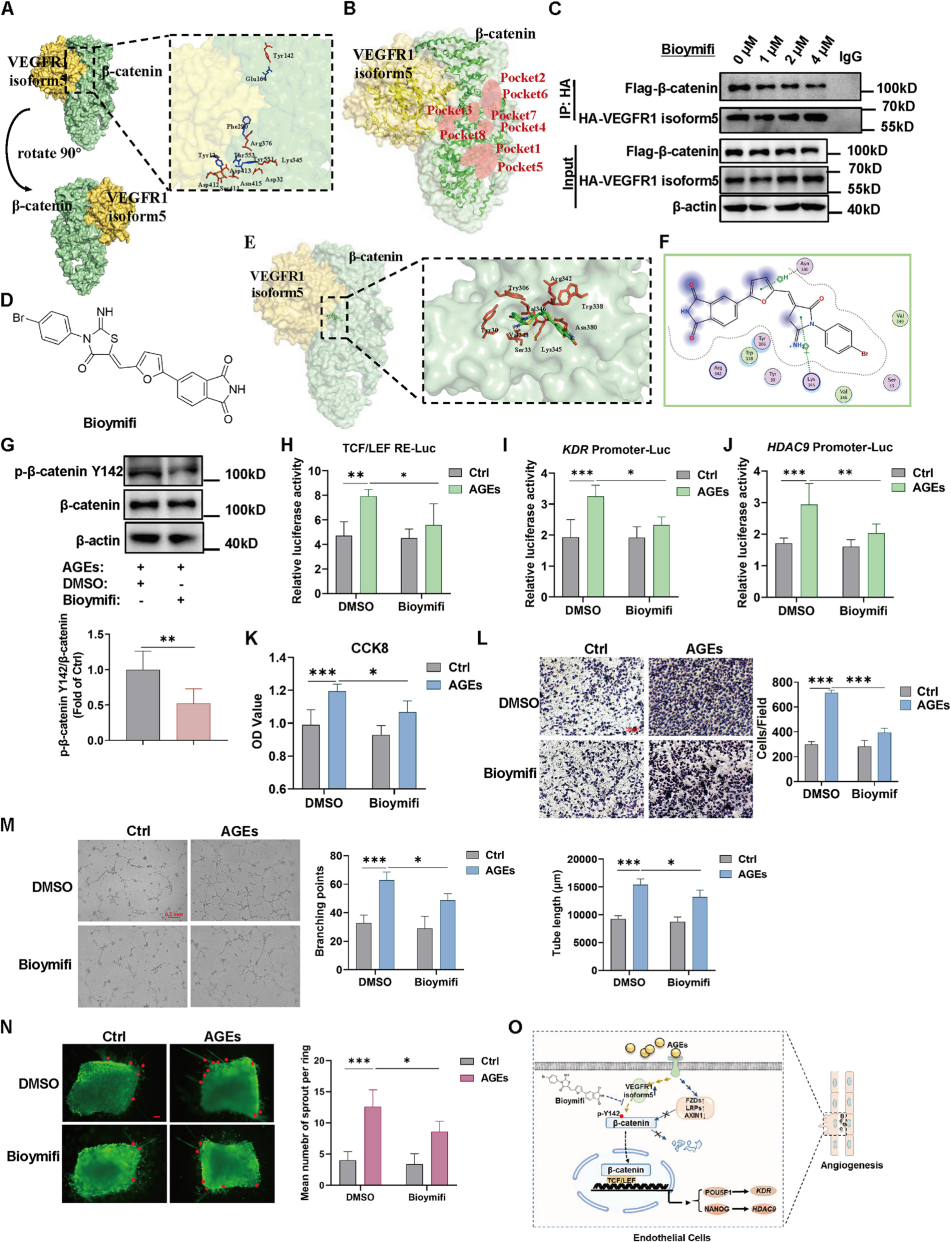
Bioymifi acts as an inhibitor of VEGFR1–β-catenin interaction and blocks AGE-induced angiogenesis. **A** The bound conformation of the VEGFR1 isoform5 and β-catenin was predicted by the ZDOCK algorithm. VEGFR1 isoform5 is displayed in yellow, and β-catenin is displayed in green. **B** Inhibitor binding pockets on the β-catenin protein mode were predicted using MOE-Site Finder plug-ins. **C** 293T cells were co-transfected with the Flag-tagged β-catenin-overexpression plasmid and HA-tagged VEGFR1 isoform5-overexpression plasmid followed by Bioymifi treatment with different concentrations in the presence of AGEs (100 μg/mL) and then Co-IP assay was performed with HA tag antibody to analyze the interaction between β-catenin and VEGFR1 isoform5. Experiments were repeated three times with similar results and one representative result is shown. **D** The molecular structure of Bioymifi. **E** Computational model of the interaction between Bioymifi and the VEGFR1 isoform5-β-catenin complex generated by AutoDock Vina and the key residues for the interaction between Bioymifi and β-catenin. **F** A 2D docking model showing the interactions between Bioymifi and β-catenin using MOE software. **G** HUVECs were pretreated with 2 μM Bioymifi for 2 h and then stimulated with AGEs (100 μg/ml) for 1 h and then β-catenin Y142 phosphorylation was measured. *n* = 6. **H**-**J** 293T cells were transfected with TCF/LEF response element (RE) (**H**), *KDR* promoter (**I**) or *HDAC9* promoter (**J**) luciferase constructs followed by AGEs (100 μg/ml) stimulation in the presence or absence of 2 μM Bioymifi. The Dual-Luciferase Reporter Assay was conducted to detected luciferase reporter activities, and luminescence was normalized to Renilla. *n* = 5. (**K**-**M**) HUVECs were treated with AGEs (100 μg/mL) for 24 h in the presence of 2 μM Bioymifi, and then the CCK8 assay (**K**), Transwell assay (**L**), and tube formation assay (**M**) were carried out to evaluate the OD value, migrated cell number and the tube length, branching points, respectively. *n* = 4 to 5, scale bar indicates 100 or 200 μm. **N** Aortic rings were treated with or without AGEs (100 μg/mL) for 6 days in the presence of 2 μM Bioymifi and then stained with isolectin B4 (IB4; green). The mean number of sprout (indicated by red dot) from aortic ring was counted. *n* = 5, scale bar indicates 100 μm. **O** Schematic diagram illustrating the function of β-catenin in AGEs-induced diabetic angiogenesis. Data are shown as *Mean* ± *SD*. *P < 0.05, **P < 0.01, ***P < 0.001

The above results implied that targeting β-catenin Y142 phosphorylation may be a potential therapeutic target for angiogenesis in diabetic microvascular complications. We then sought to identify compounds that target the VEGFR1 isoform5-β-catenin interaction and thereby inhibit β-catenin Y142 phosphorylation. ZDOCK, a computational protein–protein docking algorithm, was used to predict the bound conformation of the VEGFR1 isoform5 and β-catenin, whose structures were constructed by I-TASSER server using homologous modeling. Following ZDOCK analysis, the top-ranked bound conformation for the VEGFR1 isoform5–β-catenin complex was selected for subsequent study, and 13 core amino acids (VEGFR1 isoform5: GLU164 PHE290 TYR551 THR553 TYR12; β-catenin: TYR142 ARG376 LYS345 ASP32 ASN415 SER411 ASP412 ASP413) on the binding surface of the mode were identified (Fig. 8A). By analyzing the VEGFR1 isoform5–β-catenin binding mode with MOE-Site Finder software, two binding pockets of β-catenin (pocket 3; 8) that covered five core amino acids of the VEGFR1 isoform5–β-catenin complex were obtained on the binding surface of the complex (Fig. 8B). Next, we performed molecular docking-based virtual screening (VS) between β-catenin (pocket 3; 8) and the small molecular compounds library of MedChemExpress (MCE), which consists of over 100,000 small molecules. Considering the binding affinity, we chose the five top-ranked small molecules as candidates (Table S10), which were further screened by detecting the inhibitory effect on the VEGFR1 isoform5–β-catenin interaction using Co-IP assay. Results showed that Bioymifi exposure decreased the VEGFR1 isoform5–β-catenin interaction with increasing concentrations of Bioymifi (Fig. 8C), while the other 4 compounds did not show such effect (Fig. S7A-D). Finally, Bioymifi, which selectively and efficiently targeted β-catenin, was identified for further research (Fig. 8D-F). We found that Bioymifi decreased β-catenin Y142 phosphorylation level in HUVECs (Fig. 8G). The luciferase reporter gene result also showed a reduction of AGE-enhanced β-catenin/TCF transcriptional activity, *KDR* and *HDAC9* promoter reporter luciferase activity in the presence of Bioymifi (Fig. 8H-J). In addition, Bioymifi treatment alleviated AGE-induced increases in endothelial cell proliferation, migration, tube formation and arterial ring sprout number (Fig. 8K-N). This result suggests that Bioymifi acts as an inhibitor of VEGFR1-β-catenin interaction, inhibits β-catenin Y142 phosphorylation, *KDR* and *HDAC9* expression, and blocks AGE-induced angiogenesis.

Furthermore, considering that Bioymifi is known as a TRAIL receptor DR5 agonist [35], we next examined whether Bioymifi inhibits AGE-induced angiogenesis through binding DR5. Results showed that in the setting of AGEs treatment, DR5 knockdown with siRNA did not affect AGE-mediated proliferation, migration, and tube formation activity of HUVECs (Fig. S8A-D), suggesting that DR5 does not participate in AGE-induced angiogenesis and that Bioymifi involves in AGE-induced angiogenesis not by acting as a DR5 agonist but by inhibiting the VEGFR1-β-catenin interaction.

## Discussion

AGEs, which are the non-enzymatically glycosylated protein derivatives generated during prolonged hyperglycemic exposure, have been proven to be associated with multiple diabetic vascular complications [36]. This study concentrated on the role of β-catenin in AGE-mediated diabetic angiogenesis. Our results demonstrate that, upon AGEs stimulation, β-catenin protein level increases due to the activation of the Wnt signaling pathway. Concurrently, the expression of the upstream kinase VEGFR1 isoform5 is upregulated, which mediates the phosphorylation of the Y142 site on β-catenin. Under these conditions, β-catenin translocates into the nucleus, where it enhances its transcriptional activity and indirectly induces the expression of *KDR* and *HDAC9* through the promotion of the POU5F1/NANOG transcription factors. Furthermore, Bioymifi, a compound obtained by virtual screening, targets VEGFR1 isoform5-β-catenin interaction to inhibit Y142 phosphorylation and alleviates AGE-mediated angiogenesis (Fig. 8O). This finding provides a promising therapeutic approach for diabetic angiogenesis.

The Wnt signaling pathway has been reported to involve in cell proliferation, migration, survival, differentiation, and polarity formation [37–40]. In particular, β-catenin is a crucial component of the Wnt signaling pathway. Many studies have demonstrated that Wnt signaling pathway regulates the expression of genes associated with angiogenesis [19, 41–43]. Studies in transgenic mice indicated that many members of the Wnt signaling pathway are essential for the development of vascular morphology [37, 44]. Endothelial-specific knockout of β-catenin affects embryonic vascular development, showing signs of abnormal vascular architecture and diffuse haemorrhage, with hyperbranching in vessels such as umbilical veins and arteries [45]. Activation of β-catenin is involved in many intra-tumor angiogenesis processes and promotes tumor metastasis [46–48]. This study discovered that AGEs trigger the activation of the Wnt signaling pathway, resulting in altered levels of upstream signaling molecules within the Wnt signaling pathway at the transcript level, as well as an increase in β-catenin protein level. Accordingly, this research further explored the function of β-catenin in diabetic angiogenesis induced by AGEs.

β-catenin translocation into the nucleus serves as a basis for its transcriptional activity. In the nucleus, β-catenin binds with TCF/LEF and initiates the expression of Wnt/β-catenin target genes such as *MYC*, *CCND1*, and *AXIN2*, thereby mediating a range of biological effects [38, 49, 50]. In the present study, we discovered that β-catenin translocation into the nucleus and its transcriptional activity were enhanced upon stimulation with AGEs. In parallel, five angiogenesis-related genes were upregulated after AGEs stimulation, including *PLK2*, *JAK1*, *HDAC9*, *KDR*, and *NRP1*. *NRP1* has been reported as a target gene for β-catenin [51], while others have not been reported. However, we found that only *KDR* and *HDAC9* expression was down-regulated in response to β-catenin transcriptional activity. Thus, we speculated that *KDR* and *HDAC9* may be β-catenin target genes and are regulated by β-catenin. *KDR* encodes vascular endothelial growth factor receptor 2 (VEGFR2) protein, which is widely believed to be one of the most important regulators of angiogenesis [52–55]. HADC9 is a member of the histone deacetylases (HDACs) family and has been demonstrated to target the antiangiogenic microRNA-17–92 cluster in endothelial cells to facilitate angiogenesis [56]. However, further validation with ChIP assay showed negative results, indicating that both may not be the target genes of β-catenin. We found an indirect mechanism through public databases and siRNA interference experiments, suggesting that β-catenin may indirectly upregulate *KDR* and *HDAC9*, by upregulating the expression of the transcription factors, NANOG and POU5F1, respectively. Similarly, Martowicz et al. reported an indirect mechanism that β-catenin regulates the expression of *KDR* not directly but through another transcription factor, SOX17 [19].

It is widely believed that the phosphorylation of β-catenin plays an important role in its function [13]. Numerous signaling pathways regulate the degradation, cellular localization and function of β-catenin by changing its phosphorylation. For example, phosphorylation of S33/S37/T41/S45 in β-catenin acts as a key phosphorylation residue to mediate β-catenin degradation [57, 58]; S191/S605 phosphorylation regulates nuclear localization of β-catenin during canonical Wnt signaling activation [59]; Y654 phosphorylation breaks β-catenin/E-cadherin complex and increases Wnt signaling activity [60, 61]. In this study, a decrease in β-catenin serine/threonine phosphorylation and an increase in tyrosine phosphorylation were detected within 5 h of AGEs stimulation. It is well documented that β-catenin serine/threonine phosphorylation is closely related to its degradation [57, 58], which is consistent with our results of increased β-catenin protein level and unchanged β-catenin mRNA level after AGEs exposure. Our further experiments revealed that Y142 is a key site for β-catenin tyrosine phosphorylation after AGEs treatment and that it mediates AGE-induced angiogenesis. We have also demonstrated that β-catenin Y142 phosphorylation also takes part in the more extensive and established model of VEGF-induced angiogenesis. Therefore, its effect on angiogenesis has a certain general significance. The Y142 site is highly conserved in β-catenin and is involved in many pathophysiological processes. For example, Bai et al. discovered that Fyn activates β-catenin Y142 phosphorylation to mediate the development of osteoarthritis [62]. Our group has also previously reported that FAK phosphorylates β-catenin Y142, resulting in the disassociation of adhesion junctions and the translocation of β-catenin to initiate ADAM10 expression, which finally results in increased endothelial cell permeability [63]. Herein, we discovered a new function of β-catenin Y142 phosphorylation in modulating angiogenesis.

In this study, we have also explored the kinases that regulate β-catenin Y142 tyrosine phosphorylation under AGEs stimulation. As mentioned above, our group has previously reported that AGEs phosphorylate β-catenin Y142 through FAK kinase [63], whose identification idea and process were carried out through well-reported literature [64] followed by our validation experiments. In this study, we identified kinase using another method instead of looking in the literature. Specifically, we found kinase by intersecting two datasets obtained from the upregulated kinases in transcriptomic results and the kinases predicted in silico, respectively, and finally identified that VEGFR1 isoform5 is the kinase that mediates β-catenin Y142 phosphorylation, but the exact mechanisms involved need to be further explored. It is reported that a novel intracellular isoform of VEGFR1 (i(21)VEGFR1, VEGFR1 isoform7) activates Src and enhances cell invasion of MDA-MB-231 breast cancer cells [21]. Meanwhile, Chen et al. demonstrated that FAK directly induces β-catenin Y142 phosphorylation and promotes VEGF-mediated vascular hyperpermeability [64]. In combination of our previous findings that FAK can be activated by the AGE-RAGE-Src pathway [65], we propose that there may be a VEGFR1-SRC-FAK axis mediating β-catenin Y142 phosphorylation. Also, how AGEs induce changes in VEGFR1 expression as well as its enzymatic activity needs to be further investigated, and this is where our study plans to focus.

Based on the molecular pathway we found, we performed molecular docking-based virtual screening to identify small-molecule compounds that could block VEGFR1 isoform5–β-catenin complex interaction and further decrease Y142 phosphorylation level. One such hit in this screen is Bioymifi, which we have also shown to be a potential drug candidate to alleviate AGE-induced angiogenesis. Bioymifi, a potent TRAIL receptor DR5 activator, binds to the extracellular domain of DR5 and induces DR5 clustering and aggregation, resulting in apoptosis in human cancer cells [35]. Here, we found the other function of Bioymifi as an angiogenesis inhibitor.

## Conclusion

Taken together, these findings suggest that targeting β-catenin is a promising therapeutic strategy for angiogenesis in diabetic microvascular complications.

### Supplementary Information

**Supplementary Material 1.**

**Supplementary Material 2.**

## Data Availability

The RNA-Seq data have been deposited into the Sequence Read Archive (SRA): PRJNA976602. All data associated with this study are presented in the paper, the Supplementary Figures, the Supplementary Tables or the Supplementary Methods. Raw data not included therein can be obtained from the corresponding authors upon reasonable request.

## Abbreviations

AGEs: Advanced glycation end products;
VEGFR1: Vascular endothelial growth factor receptor 1
WT: Wild-type
UEA-I: Ulex europaeus agglutinin I
DEGs: Differentially expressed genes
GO: Gene Ontology
KEGG: Kyoto Encyclopedia of Genes and Genomes
NC: Negative control
ChIP: Chromatin immunoprecipitation
Ad: Adenovirus
IP: Immunoprecipitation
FZDs: Frizzled receptors
LRPs: LDL Receptor Related Proteins
KDR: Kinase Insert Domain Receptor
HDAC9: Histone Deacetylase 9
FLT1: Fms-Like Tyrosine Kinase 1
TCF: T Cell Factor
LEF1: Lymphoid Enhancer Binding Factor 1
PLK2: Polo Like Kinase 2
PTGS2: Prostaglandin-Endoperoxide Synthase 2
JAK1: Janus Kinase 1
SMAD1: SMAD Family Member 1
NRP1: Neuropilin 1
CCND1: Cyclin D1
