# Supplementary material for: Endothelial β-catenin upregulation and Y142 phosphorylation drive diabetic angiogenesis via upregulating KDR/HDAC9
Source: Cell Commun Signal. 2024 Mar 15;22:182. doi: 10.1186/s12964-024-01566-1 (PMC10941375; doi:10.1186/s12964-024-01566-1)
Supplement: Supplementary file 1 — Supplementary Material 1. [file 12964_2024_1566_MOESM1_ESM.pdf]

Supplementary Information

Endothelial β-catenin Upregulation and Y142 Phosphorylation Drive Diabetic Angiogenesis via Upregulating *KDR*/*HDAC9*

Zhenfeng Chen, Bingqi Lin, Xiaodan Yao, Jie Weng, Jinlian Liu, Qi He, Ke Song, Chuyu Zhou, Zirui Zuo, Xiaoxia Huang, Zhuanhua Liu, Qiaobing Huang, Qiulin Xu, Xiaohua Guo

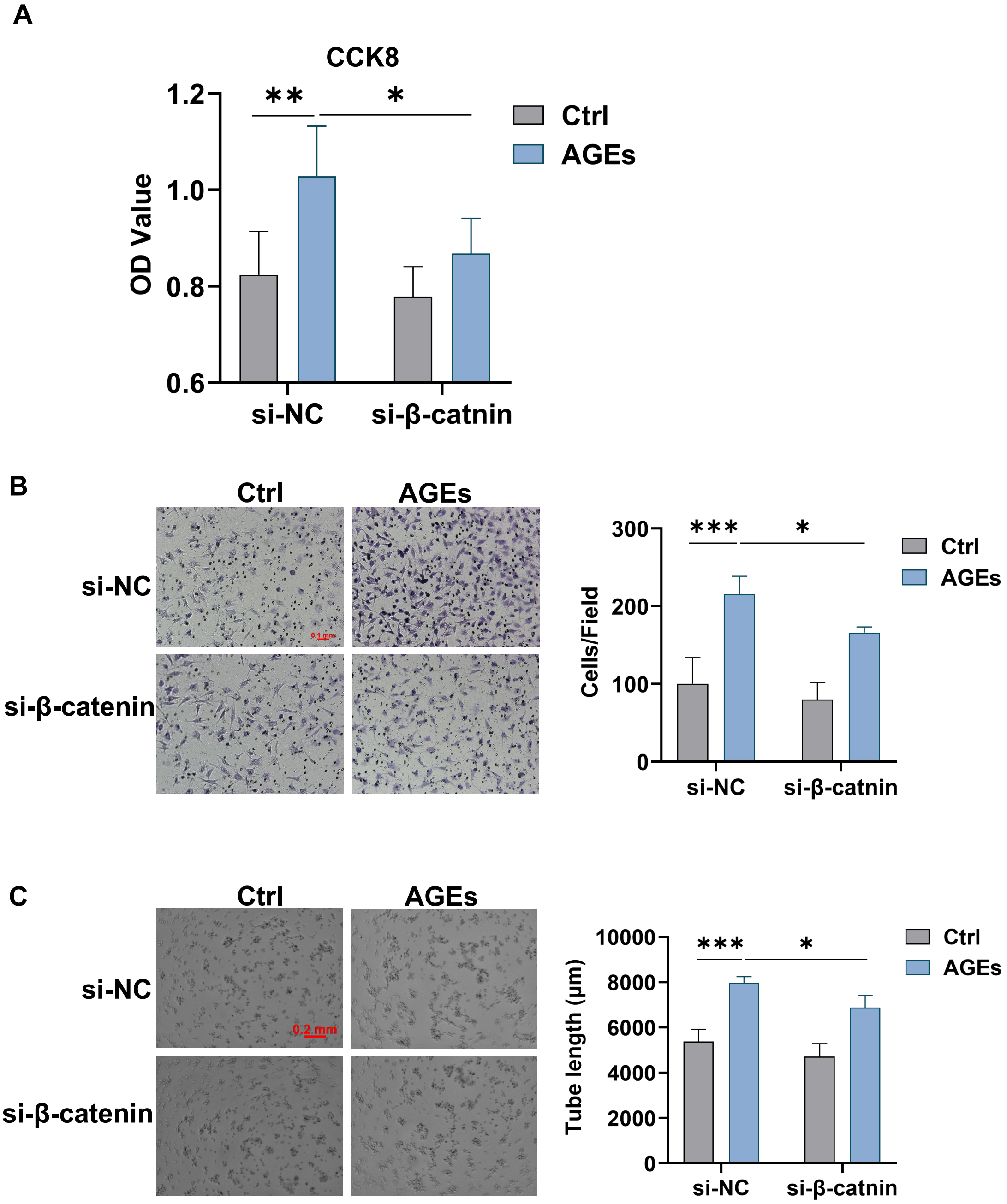

Fig. S1 Effects of β-catenin on AGE-induced angiogenesis. After transfection with negative control (NC) siRNA or with specific siRNA targeting β-catenin for 48 h, HUVECs were stimulated with AGEs (100 μg/ml) for 24 h followed by the CCK8 assay **(A)**, Transwell assay **(B)**, and tube formation assay **(C)** to evaluate the OD value, migrated cell number and tube length, respectively. *n* = 4 to 5, scale bar indicates 100 or 200 μm. Data are shown as *Mean* ± *SD*. **P*<0.05, ***P*<0.01, ****P*<0.001.

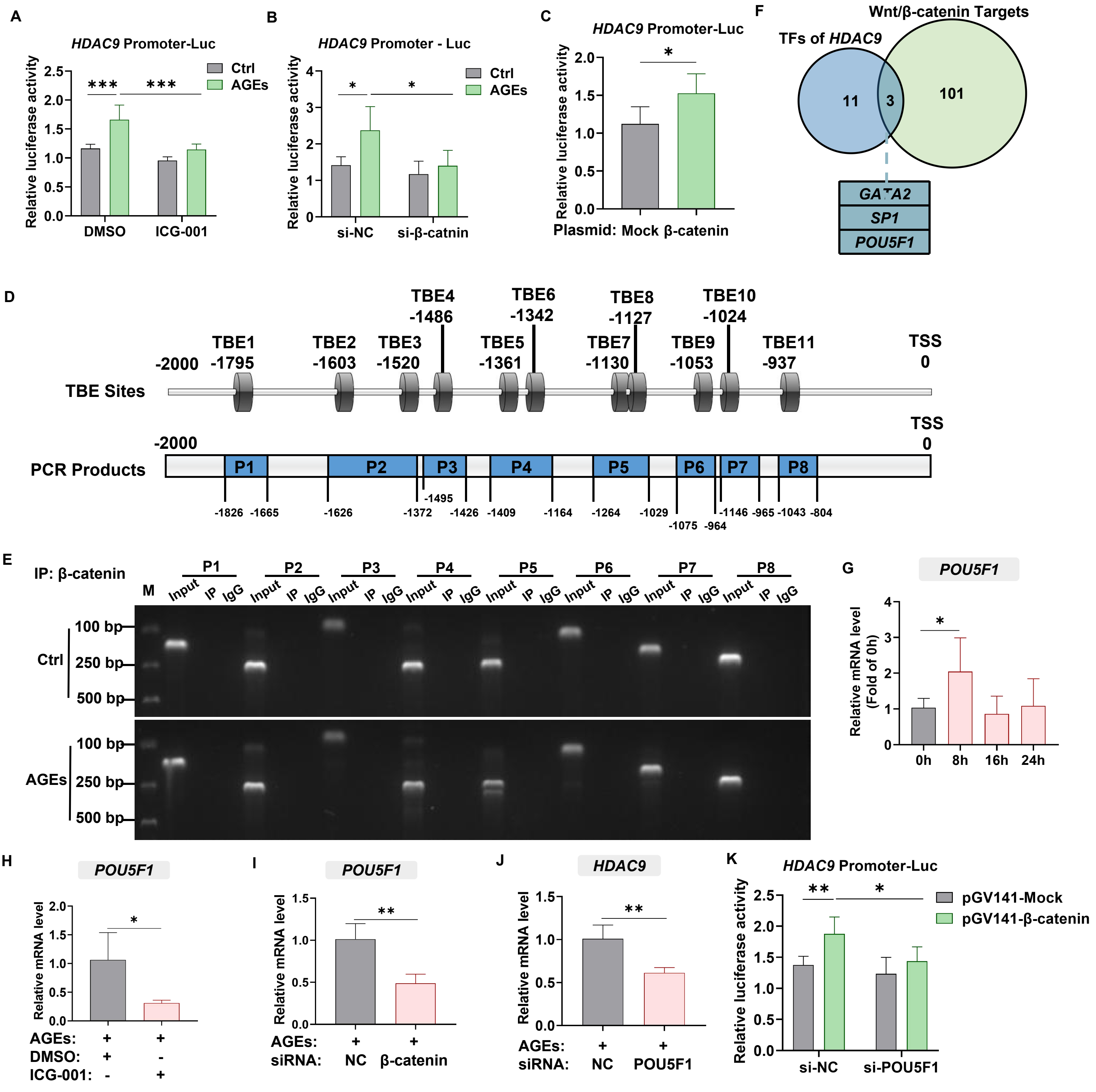

Fig. S2 β-catenin enhances the expression of *HDAC9* by upregulating POU5F1 under AGEs treatment. (A-B) 293T cells were transfected with pGL3 plasmid containing *HDAC9* promoter DNA sequence followed by AGEs stimulation in the presence or absence of 20 μM ICG-001 for 24 h **(A)** or β-catenin siRNA **(B)**. The Dual-Luciferase Reporter Assay was applied to detect *HDAC9* promoter activity. n = 5. **(C)** 293T cells were co-transfected with *HDAC9* promoter luciferase construct and β-catenin-overexpression plasmid. After 48 h, the Dual-Luciferase Reporter Assay was performed to detect promoter activity. n = 5. **(D)** Schematic diagram of the predicted binding region of β-catenin to *HDAC9* promoter DNA sequence and the PCR amplification product location of 8 primers for the predicted binding element. **(E)** The binding of β-catenin to the *HDAC9* promoter in response to AGEs treatment was determined by ChIP assay and quantified by PCR. Experiments were repeated three times with similar results and one representative result is shown. **(F)** Venn diagram of overlapping genes between transcriptional factors of *HDAC9* and Wnt/β-catenin targets. **(G)** HUVECs were treated with AGEs for different times and POU5F1 mRNA levels were detected by qPCR. n = 6. **(H-I)** HUVECs were treated in the presence of ICG-001(20 μM) **(H)** or β-catenin siRNA **(I)**, and POU5F1 mRNA levels were detected by qPCR. n = 4. **(J)** siRNA targeting POU5F1 was transfected into HUVECs followed by AGEs treatment and *HDAC9* mRNA levels were detected by qPCR, respectively. n = 4. **(K)** 293T cells were co-transfected with *HDAC9* promoter luciferase construct, β-catenin-overexpression plasmid and POU5F1 siRNA. After 48 h, the Dual-Luciferase Reporter Assay was used to detect *HDAC9* promoter activity. n = 6. Data are shown as Mean ± SD. *P<0.05, **P<0.01, ***P<0.001.

>sp|P35222| Catenin beta-1 OS=Homo sapiens GN=CTNNB1

```
1   MATQADLMELDMAMEPDRKAAVSHWQQQSYLDSGIHSGATTTAPSLSGKGNPEEEDVDTS 60
61  QVLYEWEQGFSQSFTQEQVADIDGQYAMTRAQRVRAAMFPETLDEGMQIPSTQFDAAHPT 120
121 NVQRLAEPSQMLKHAVVNLINYQDDAELATRAIPELTKLLNDEDQVVVNKAAVMVHQLSK 180
181 KEASRHAIMRSPQMVSAIVRTMQNTNDVETARCTAGTLHNLSHHREGLLAIFKSGGIPAL 240
241 VKMLGSPVDSVLFYAITTLHNLLLHQEGAKMAVRLAGGLQKMVALLNKTNVKFLAITTDC 300
301 LQILAYGNQESKLIILASGGPQALVNIMRTYTYEKLLWTTSRVLKVLSVCSSNKPAIVEA 360
361 GGMQALGLHLTDPSQRLVQNCLWTLRNLSDAATKQEGMEGLLGTLVQLLGSDDINVVTCA 420
421 AGILSNLTCNNYKNKMMVCQVGGIEALVRTVLRAGDREDITEPAICALRHLTSRHQEAEM 480
481 AQNAVRLHYGLPVVVKLLHPPSHWPLIKATVGLIRNLALCPANHAPLREQGAIPRLVQLL 540
541 VRAHQDTQRRTSMGGTQQQFVEGVRMEEIVEGCTGALHILARDVHNRIVIRGLNTIPLFV 600
601 QLLYSPIENIQRVAAGVLCELAQDKEAAEAIEAEGATAPLTELLHSRNEGVATYAAAVLF 660
661 RMSEDKPQDYKKRLSVELTSSLFRTEPMAWNETADLGLDIGAQGEPLGYRQDDPSYRSFH 720
721 SGGYGQDALGMDPMMEHEMGGHHPGADYPVDGLPDLGHAQDLMDGLPPGDSNQLAWFDTDL 781
```

17 Tyrosine (Y) sites						
Y30	Y64	Y86	Y142			
Y254	Y306	Y331	Y333	Y432	Y489	
Y604	Y654	Y670	Y709	Y716	Y724	Y748

Fig. S3 The amino acid sequence of β-catenin from the Uniprot database. Letter with yellow shading indicates tyrosine (Y), which is counted as 17.

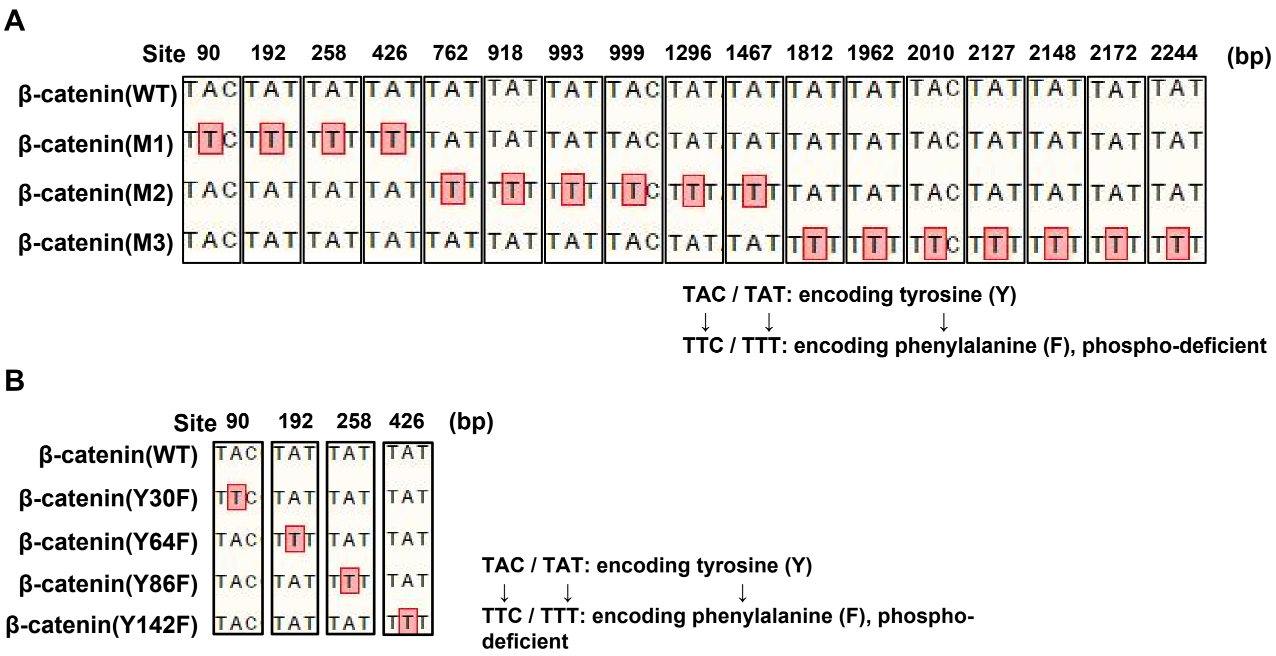

Fig. S4 The sequence result of the construct shows that the sites were mutated as expected. Alignment is performed with SnapGene software (GSL Biotech; available at snapgene.com).

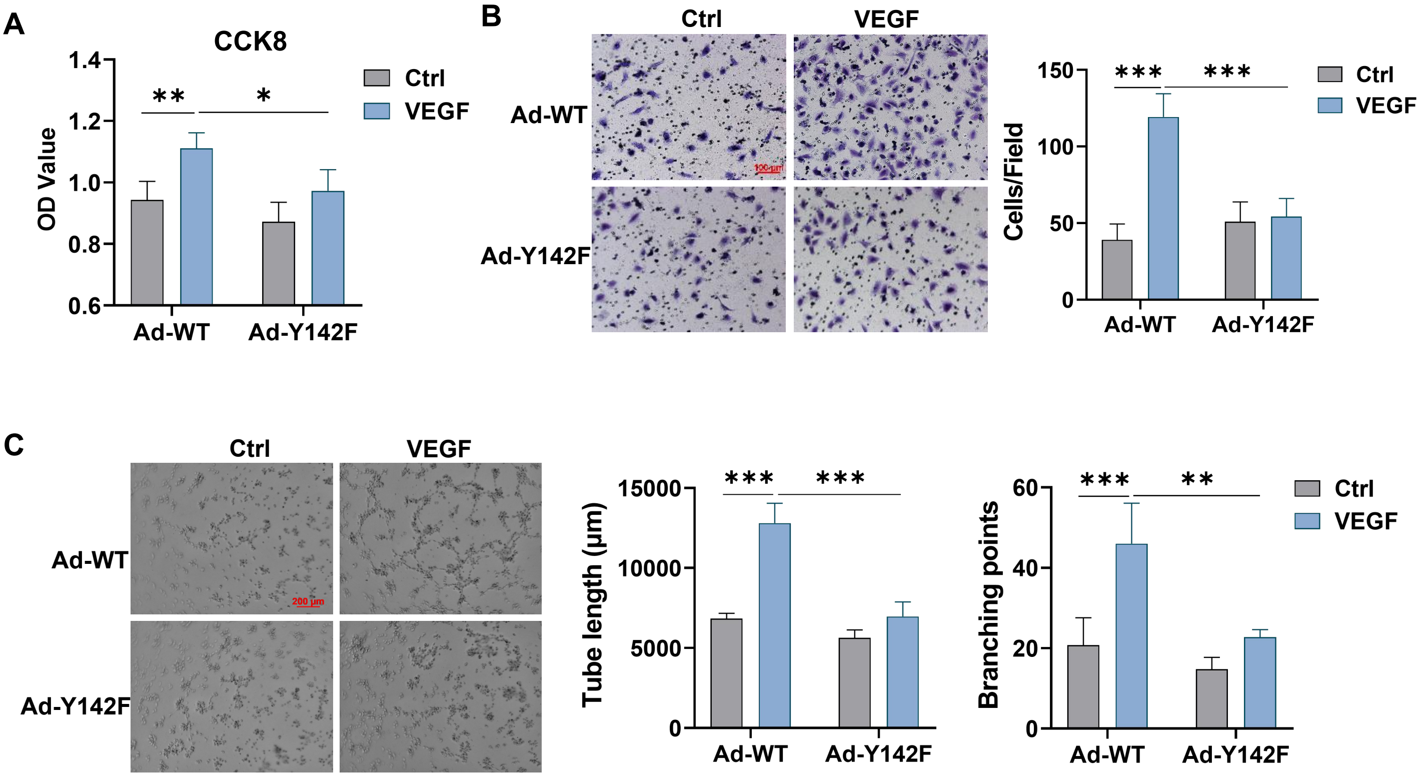

Fig. S5 β-catenin Y142 phosphorylation is involved in VEGF-induced angiogenesis. (A-C) After transfected with β-catenin WT or Y142F overexpression adenovirus for 48 h, HUVECs were treated with VEGF165 (25 ng/ml) for 24 h followed by the CCK8 assay **(A)**, Transwell assay **(B)**, and tube formation assay **(C)** to evaluate the OD value, migrated cell number and tube length, branching points, respectively. $n = 4$ to 5, scale bar indicates 100 or 200 μm. Data are shown as *Mean* ± *SD*. * P<0.05, **P<0.01, **P<0.001

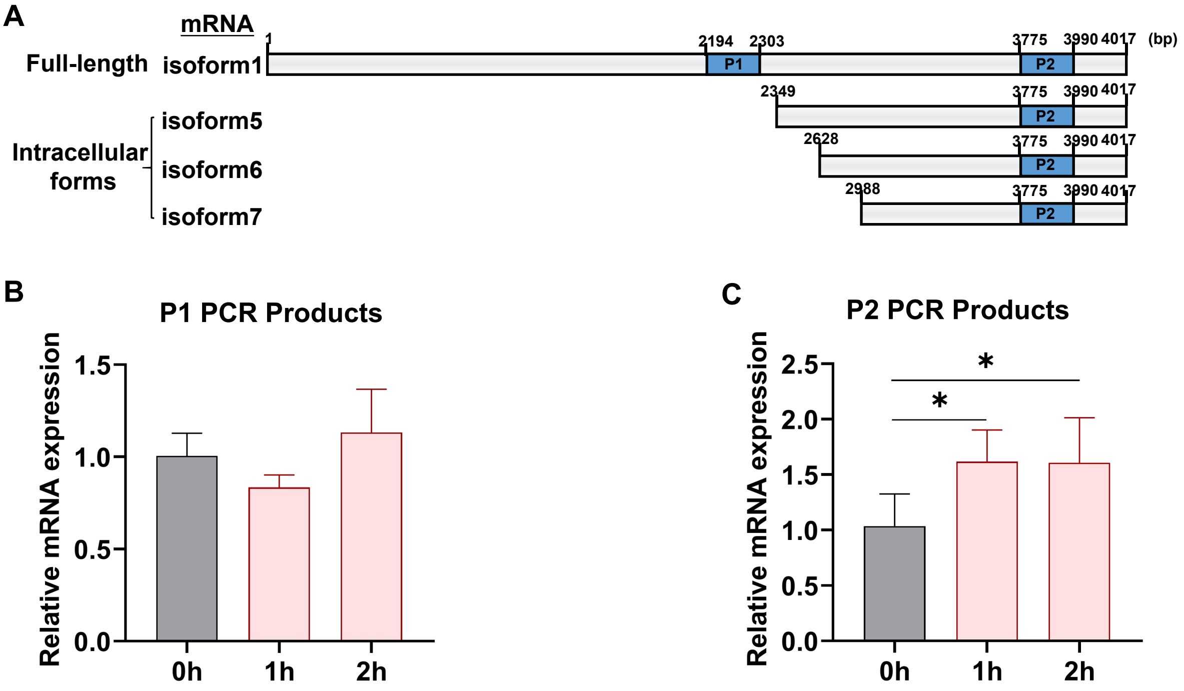

Fig. S6 The two types of VEGFR1 isoforms mRNA expression levels induced by AGEs in HUVECs. **(A)** Schematic diagram showing the qPCR amplification product location of 2 primer pairs for the VEGFR1 mRNA. P1 or P2 with the blue background indicates the qPCR amplification product of the corresponded primer. **(B)** HUVECs were stimulated by AGEs (100 μg/mL) for 1 h and then the full-length VEGFR1 mRNA level was detected by P1 PCR products using qPCR. $n = 5$. **(C)** HUVECs were stimulated by AGEs (100 μg/mL) for 1 h. Then the intracellular isoforms and full-length mRNA level of VEGFR1 were detected by P2 PCR products using qPCR. $n = 5$. Data are shown as *Mean* ± *SD*. * $P<0.05$.

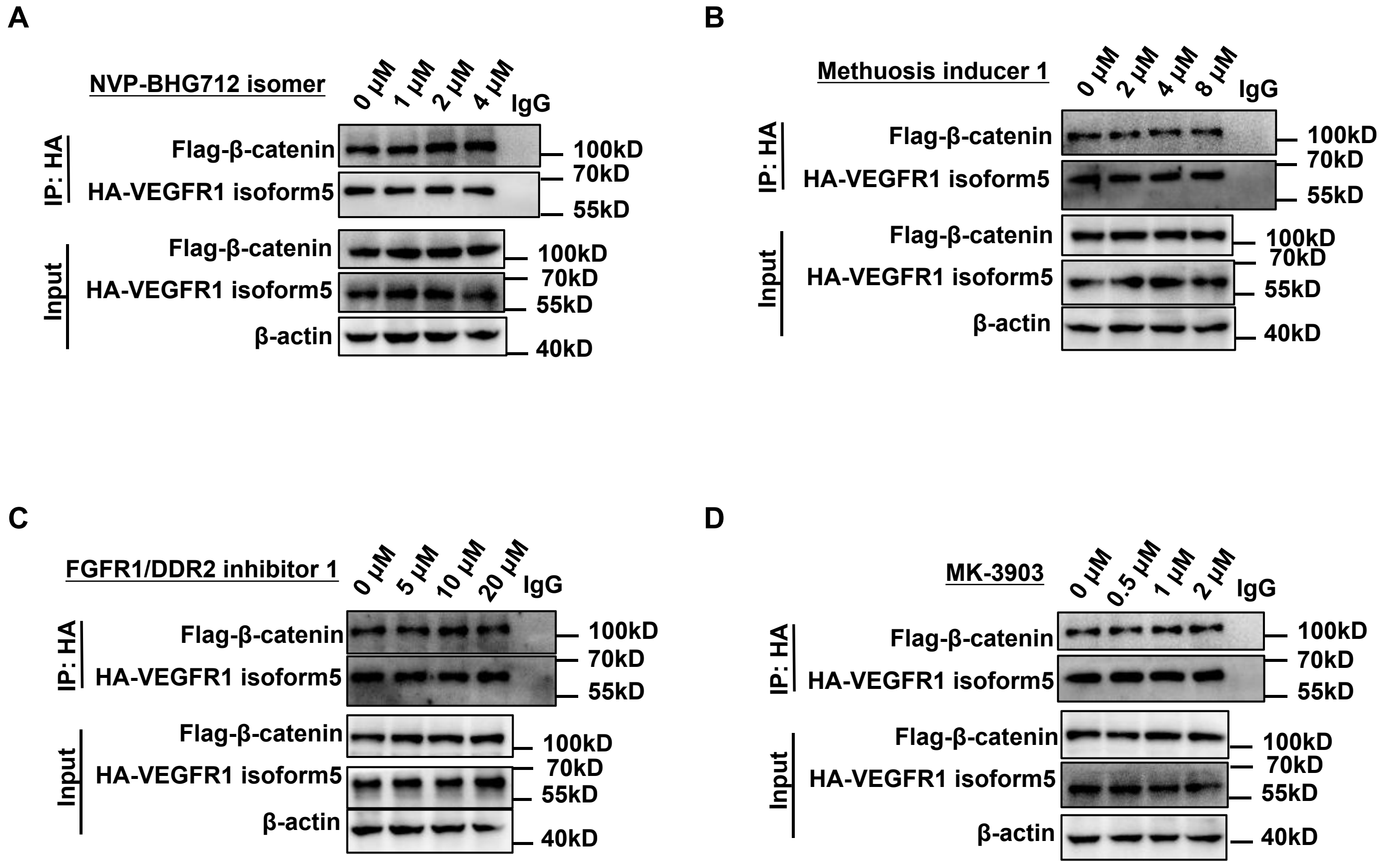

Fig. S7 Inhibitory Effects on VEGFR1 isoform5–β-catenin interaction among the top five selected compounds. 293T cells were co-transfected with Flag-tagged β-catenin-overexpression plasmid and HA-tagged VEGFR1 isoform5-overexpression plasmid followed by NVP-BHG712 isomer **(A)**, Methuosis inducer 1 **(B)**, FGFR1/DDR2 inhibitor 1 **(C)**, or MK-3903 **(D)** treatment of different doses as indicated with the presence of AGEs (100 μg/mL) and then Co-IP assay was performed with HA tag antibody to analyze the interaction between β-catenin and VEGFR1 isoform5. Experiments were repeated three times with similar results and one representative experiment is shown.

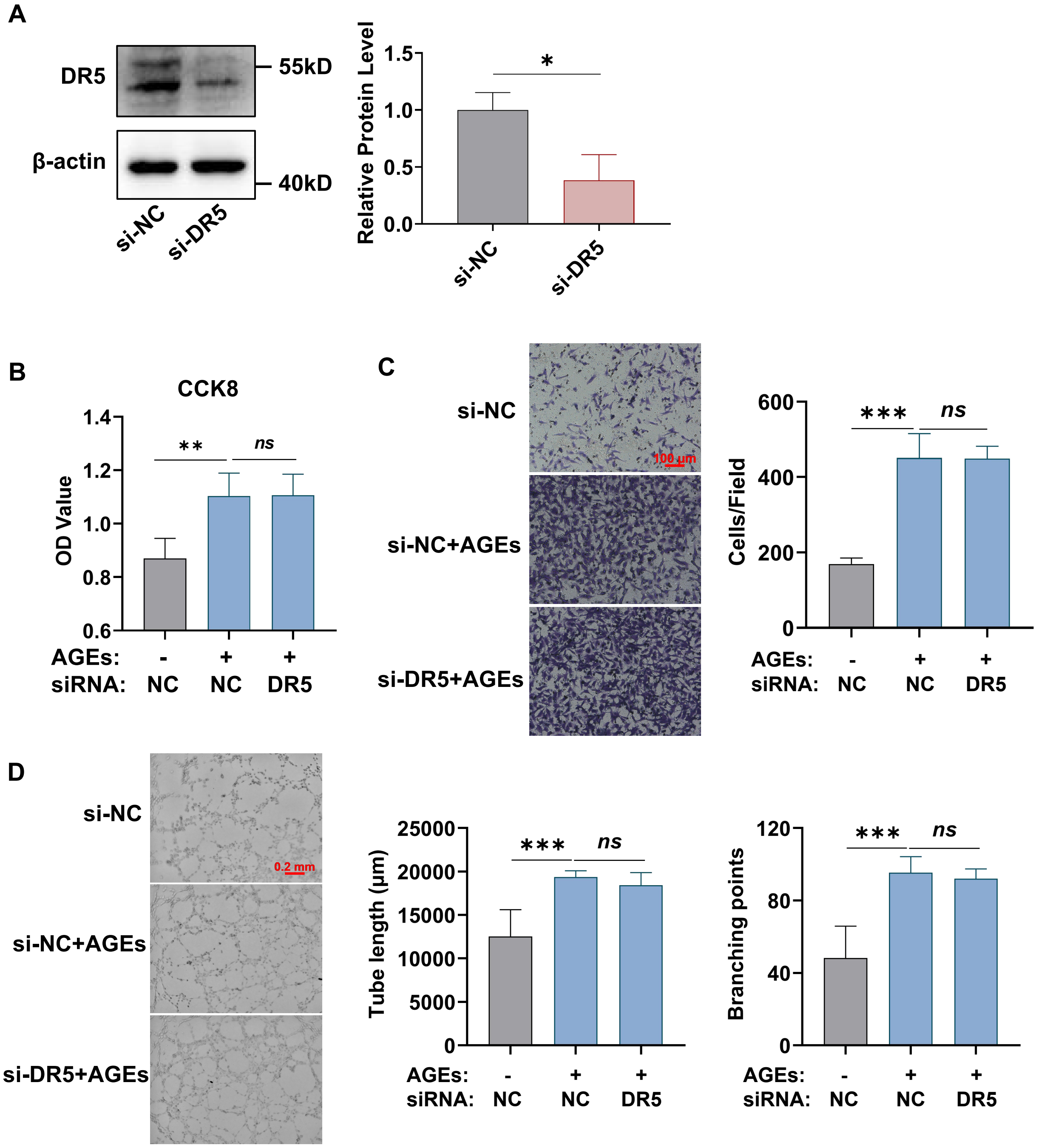

Fig. S8 Effects of DR5 on AGE-induced angiogenesis. After transfection with negative control (NC) siRNA or with specific siRNA targeting DR5 for 48 h, HUVECs were stimulated with AGEs (100 μg/ml) for 24 h followed by the CCK8 assay **(A)**, Transwell assay **(B)**, and tube formation assay **(C)** to evaluate the OD value, migrated cell number and tube length, respectively. $n = 3$ to 5, scale bar indicates 100 or 200 μm. Data are shown as *Mean* ± *SD*. *P<0.05, **P<0.01, ***P<0.001.

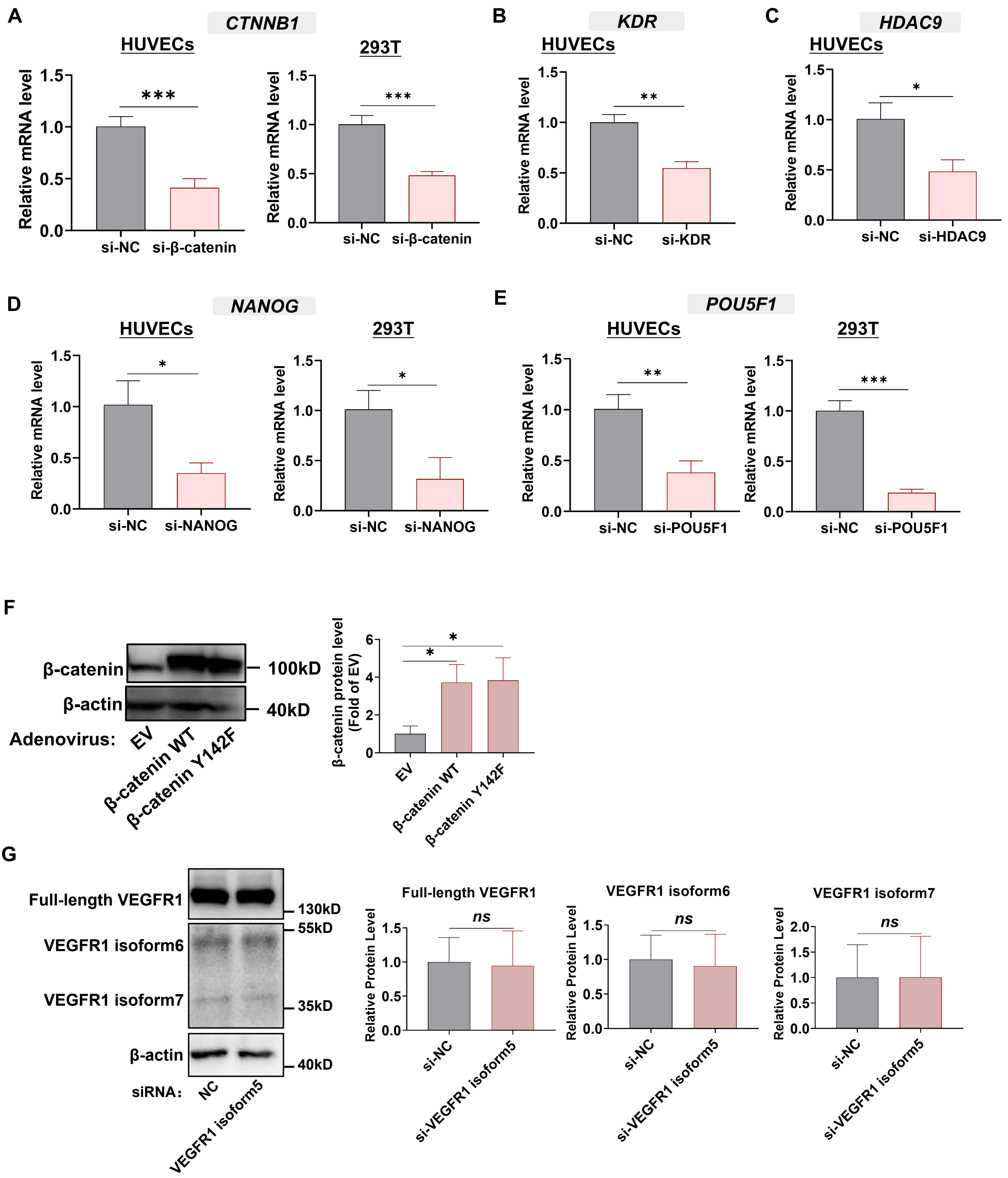

Fig. S9 Knockdown or overexpression efficiency validation. (A-E) Specific siRNA targeting the gene was transfected into cells and the knockdown efficiency was verified by qPCR. **(F)** Adenovirus overexpression efficiency of empty vector (EV), Ad-WT and Ad-Y142F in HUVECs was measured by western blot. **(G)** The full-length VEGFR1, VEGFR1 isoform6 and VEGFR1 isoform7 expression level in HUVECs transfected with siRNA targeting VEGFR1 isoform5. *n* = 3 to 6. Data are shown as *Mean* ± *SD*. **P*<0.05, ***P*<0.01, ***P*<0.001

Supplementary Tables

Table S1. The information on key materials used in this study

Reagent	Source	Identifier
**Antibodies**		
Anti-β-catenin	Proteintech	Cat# 66379-1-Ig<br>RRID:AB_2857358
Anti-β-catenin(ChIP Grade)	Cell Signaling	Cat# 8480<br>RRID:AB_11127855
Anti-Pan Phospho-Serine/Threonine	Abclonal	Cat# AP1067<br>RRID:AB_2863939
Anti-Pan Phospho-Tyrosine	Abclonal	Cat# AP0905<br>RRID:AB_2770784
Anti-β-catenin Y142 phophos	Affinity	Cat# AF8101<br>RRID:AB_2840164
Anti-Flag	Sigma	Cat# F1804<br>RRID:AB_262044
Anti-HA	Proteintech	Cat# 51064-2-AP<br>RRID:AB_11042321
Anti-HA-HRP Conjugated	Abclonal	Cat# AE025<br>RRID:AB_2769866
Anti-VEGFR1	Immunoway	Cat# YT1728
UEA-I	Vector Laboratories	Cat# FL-1061<br>RRID:AB_2336767
Isolectin B4	Sigma	Cat# L2895
**Chemicals and Recombinant Protein**		
ICG-001	Selleck	Cat# S2662
NVP-BHG712 isomer	TargetMol	Cat# T19487
Methuosis inducer 1	TargetMol	Cat# T7262
FGFR1/DDR2 inhibitor 1	TargetMol	Cat# T11279
Bioymifi	MedChemExpress	Cat# HY18377
MK-3903	TargetMol	Cat# T5187
VEGF165	Genscript	Cat# Z02689
**Recombinant DNA**		
pGV141-Flag-β-catenin WT	GENECHEM	N/A
pGV141-Flag-β-catenin multiple tyrosine substitution mutant (M1)	This paper	N/A
pGV141-Flag-β-catenin multiple tyrosine substitution mutant (M2)	This paper	N/A
pGV141-Flag-β-catenin multiple tyrosine substitution mutant (M3)	This paper	N/A
pGV141-Flag-β-catenin Y30F	This paper	N/A
pGV141-Flag-β-catenin Y64F	This paper	N/A
pGV141-Flag-β-catenin Y86F	This paper	N/A
pGV141-Flag-β-catenin Y142F	This paper	N/A
pcDNA3.1-HA-VEGFR1 isoform5	This paper	N/A

pcDNA3.1-HA-VEGFR1 isoform6	This paper	N/A
pcDNA3.1-HA-VEGFR1 isoform7	This paper	N/A
pGL4.49-TCF-LEF RE/luc2P Vector	Promega	Cat# E4611
pGL3-Basic Vector	Promega	Cat# E1751
pGL3-*KDR* promoter/luc Vector	This paper	N/A
pGL3-*HDAC9* promoter/luc Vector	This paper	N/A
pGL4.74-hRluc/TK Vector	Promega	Cat# E6921

Table S2. Predicted binding region of β-catenin/TCF to *KDR* promoter DNA sequence

No.	Predicted sequence	Start	End	Strand	Score
TBE1	AATCTTTAAAGAAA	-1833	-1820	-	6.13002
TBE2	AAAGATGGAAGGAT	-1743	-1730	+	7.35997
TBE3	GAGCTTTAAAGGTT	-1630	-1617	+	4.37088
TBE4	CAAATTTAAAGGGA	-1450	-1437	+	4.40725
TBE5	AGGGAAAAAAGCCA	-1441	-1428	+	5.03423
TBE6	GGAAAAAAAAGGAA	-1346	-1333	+	4.25918
TBE7	AAACATAAAAATGC	-1334	-1321	+	6.72006
TBE8	CAAGTTGACAGGTG	-1220	-1207	-	4.618
TBE9	GGAGGTAAAAGACA	-1035	-1022	+	4.6043
TBE10	TGACATCAAATGTC	-984	-971	-	6.40035

TBE: β-catenin/TCF Binding Elements

Table S3. ChIP-PCR primer sequences for amplifying the β-catenin/TCF binding region to *KDR* promoter

No.	PCR Products	Forward primer (5'-3')	Reverse primer (5'-3')	Length (bp)
TBE1	P1	CCCTCTCCAAACCAGGTTCC	TGCGAACAAAGCTCCATCCT	216
TBE2	P2	AAGATTCTCTCCCCTCCCCAAA	TGCGAACAAAGCTCCATCCTT	111
TBE3	P3	GATGGAGCTTTGTTCGCAGC	CACTGGAGATTCGTTCTACATCA	138
TBE4<br>TBE5	P4	GATGTAGAACGAATCTCCAGTGT	GTCACCCTTCTCTTCCCACA	238
TBE6<br>TBE7	P5	ACCAAGCAATTGTGGGAAGAGAA	TTGGCAGAGGCATTTTTATGTTTC	97
TBE8	P6	GGAAACATAAAAATGCCTCTGCCA	GTATTCACGCTTTTACTTTTCCAAG	151
TBE9	P7	ATCTTGGTGAAGAATGGTCCTT	ACCACATTCGTTCCTTCTGGT	165
TBE10	P8	TGGTGAAGAATGGTCCTTTAGGTT	AGGGGAACTCCAATTCCTTCC	184

Table S4. Transcriptional factors that regulates *KDR* expression from the public database

No.	Gene name	Reference
1	*PRH*	PMID: 28604763
2	*HEY1*	PMID: 12453432
3	*GATA2*	PMID: 26877867
4	*EPAS1*	PMID: 12464608
5	*ETS1*	PMID: 12464608
6	*NFKB1*	PMID: 10864920
7	*SP1*	
8	*SP3*	PMID: 16678129
9	*SP4*	
10	*NANOG*	PMID: 21119109
11	*E2F1*	PMID: 20516113

Table S5. β-catenin target genes from public database

No.	Gene name	Reference	No.	Gene name	Reference
1	*MYC*	https://web.stanford.edu/~rnusse/pathways/targets.html	53	*MMP9*	https://web.stanford.edu/~rnusse/pathways/targets.html
2	*CCND1*		54	*SIAMOIS*	
3	*TCF7*		55	*BMP4*	
4	*LEF1*		56	*EN2*	
5	*PPARD*		57	*GJA1*	
6	*JUN*		58	*GJB6*	
7	*FOSL1*		59	*RXRG*	
8	*MMP7*		60	*STRA6*	
9	*AXIN2*		61	*RHOU*	
10	*NRCAM*		62	*TWIST1*	
11	*ITF2*		63	*MMP3*	
12	*GAST*		64	*CCN4*	
13	*CD44*		65	*TBXT*	
14	*EPHB2*		66	*GCG*	
15	*BMP4*		67	*BGLAP*	
16	*CLDN1*		68	*CDX1*	
17	*BIRC5*		69	*PTGS2*	
18	*VEGFA*		70	*IRX3*	
19	*FGF18*		71	*SIX3*	
20	*ATOH1*		72	*NEUROG1*	
21	*MET*		73	*SFTPC*	
22	*EDN1*		74	*NEUROD1*	
23	*MYCBP2*		75	*NKX2-2*	
24	*L1CAM*		76	*GBX2*	
25	*ID2*		77	*CCN5*	
26	*JAG1*		78	*IGF2*	
27	*TIAM1*		79	*EMP2*	
28	*NOS2*		80	*IGF1*	
29	*DKK1*		81	*VEGFC*	
30	*FGF9*		82	*ABCB1*	
31	*FGF20*		83	*IL6*	
32	*LGR5*		84	*CDX4*	
33	*SOX9*		85	*SFRP2*	
34	*SOX17*		86	*PITX2*	
35	*RUNX2*		87	*AGRE1*	
36	*GREM2*		88	*EDA*	
37	*SALL4*		89	*CDH1*	
38	*CCN1*		90	*CDKN2A*	
39	*SOX2*		91	*CTLA4*	

40	*PTTG*	92	*FGF4*	
41	*DLL1*	93	*CXCL8*	
42	*FOXN1*	94	*RET*	
43	*MMP26*	95	*VCAN*	
44	*NANOG*	96	*TNFRSF19*	
45	*POU5F1*	97	*UBXN1*	
46	*SNAI1*	98	*EN1*	
47	*FN1*	99	*IKBKG*	
48	*FZD7*	100	*FOXC2*	PMID: 27313318
49	*FSTL*	101	*PROX1*	PMID: 27313318
50	*WNT3A*	102	*GATA3*	PMID: 19648923
51	*ISL1*	103	*GATA2*	PMID: 30332639
52	*MMP2*	104	*SP1*	PMID: 24217507

Table S6. Predicted binding region of β-catenin/TCF to *HDAC9* promoter DNA sequence

No.	Predicted sequence	Start	End	Strand	Score
TBE1	CATTTTGAAAGGTA	-1795	-1782	+	6.44438
TBE2	ATACTCCAAAGGCT	-1603	-1590	-	6.00666
TBE3	TTAGAGCAAAGTGA	-1520	-1507	+	4.60614
TBE4	GAGGATAAAAGTGT	-1486	-1473	-	7.51837
TBE5	CCATTTCAAACAAA	-1361	-1348	-	4.77267
TBE6	GGGCAACAAAGTGA	-1342	-1329	-	7.48865
TBE7	GATCAAGAAAGACC	-1130	-1117	-	6.13665
TBE8	AAAGATCAAGAAAG	-1127	-1114	-	5.98148
TBE9	AAATATGAAAGCAG	-1053	-1040	+	10.4131
TBE10	GACCATCAAACGAA	-1024	-1011	-	7.65267
TBE11	ACACTTAAAAAAAT	-937	-924	+	6.55782

TBE: β-catenin/TCF Binding Elements

Table S7. ChIP-PCR primer sequences for amplifying the β-catenin/TCF binding region to *HDAC9* promoter

No.	PCR Products	Forward primer (5'-3')	Reverse primer (5'-3')	Length (bp)
TBE1	P1	AAAGGCTGGACACTTGACCATT	ACGAATCGTAGTAATGGGTTCTCA	162
TBE2<br>TBE3	P2	TAAGCAGAAGCTCTAGCACCAAC	CCTCAGGAATAAAGCCAGACTAC	255
TBE4	P3	AGCCATCATACACTTTTATCCTCCT	AGGAGTGGTATGGTAGCTGT	70
TBE5<br>TBE6	P4	GCTGTTATGAAATTGGTAGTCTGGC	TCGGATCCATGGGAGTAGTGT	246
TBE7<br>TBE8	P5	TATAGGCTTGAGCTACCGGG	AGCTGGTGTCTCTGCTTTCA	236
TBE9	P6	AGCTGACGTAGAGGAAAACCA	AACAGTTGATCACTCAAAGAGGC	112
TBE10	P7	TGAGGGTACTGGGTTAGGTCT	ACAGTTGATCACTCAAAGAGGC	182
TBE11	P8	GCAGAGACACCAGCTTTAATTCG	GGTGATCTCCTTCAGTGGTTAGT	240

Table S8. Transcriptional factors that regulate *HDAC9* expression from the public database

No.	Gene_name	Reference
1	*GATA3*	PMID: 23524580
2	*MEF2A*	PMID: 17101791
3	*MEF2C*	
4	*MEF2D*	
5	*EGR1*	PMID: 26791815
6	*NFKB1*	
7	*GATA2*	
8	*PAX5*	
9	*SP1*	
10	*YY1*	
11	*POU5F1*	
12	*ZNF263*	
13	*MAX*	
14	*BCL11A*	

Table S9. The gene names of the predicted kinases *in silico*, the AGE-upregulated kinases, and their intersection kinases

Item	Count	Genes
Predicted Kinases *in silico* & AGE-Upregulated Kinases	1	*FLT1*
Predicted Kinases *in silico*	30	*TNK2 MATK EPHA4 FGFR2 FGFR3 PTK2 FER CSF1R NTRK1 NTRK2 MAPK1 CMGC TKL EGFR ERBB2 EPHA2 EPHB2 INSR TYK2 PDGFRA PTK6 FGR BLK HCK BMX TEK FLT1 BAZ1B CAMKL CAMK WEE1B*
AGE-Upregulated Kinases	106	*FAM20C LYN SPHK1 NUAK1 SGK1 MAP3K5 STK38L TYRO3 CSNK1A1 PIK3R3 JAK1 DDR1 FLT1 DGKZ DYRK3 MADD PRKAG2 CDK12 ALPK3 IPPK WEE1 PRKD3 MAP3K7 DYRK1A GRK5 SGK3 CDKN1A TLK1 CHKA HIPK2 MAP3K1 DGKZP1 CMPK2 RIOK2 PXK MARK1 PIK3AP1 TLK2 MAP2K4 PRKAR2A TBK1 MAP4K5 CERK PRKACB IP6K2 PRKAR1A NEK7 MAP3K8 ROR1 PDK1 MAP2K3 PFKFB2 CMPK1 PIP5K1A MAPK1IP1L ETAA1 CKS2 HIPK3 FASTKD5 PRKD1 TAOK3 PRKACA PIK3C2A PFKP TP53RK STK17B TAB2 MAGI3 CLK2 FASTKD2 PANK1 CSNK1G3 STK38 PIK3CA RIOK1 ICK MAPK12 WNK1 PDPK1 IBTK CDK2AP2P2 MOB1A MAPK9 CDK1 PANK3 STRAP LATS1 RPS6KA5 BRSK2 BRAF PRKCH STK39 CHUK CDK5 MAP3K2 MNAT1 PRKAA1 AKIP1 ETNK1 AK3P5 MAPKAPK5 SIK2 AURKA MAPK10 MARK4 PIK3C3 MAP3K20*

Table S10. Top 30 small-molecule compounds selected from MedChemExpress (MCE) library by virtual screening

Rank	Name	Binding Affinity	CAS	Molecule Information
1	NVP-BHG712 isomer	-11	2245892-85-5	https://pubchem.ncbi.nlm.nih.gov/compound/117602383
2	Methuosis inducer 1	-10.9	2240205-30-3	https://pubchem.ncbi.nlm.nih.gov/compound/138319688
3	FGFR1/DDR2 inhibitor 1	-10.8	2308497-58-5	https://pubchem.ncbi.nlm.nih.gov/compound/138454761
4	Bioymifi	-10.7	1420071-30-2	https://pubchem.ncbi.nlm.nih.gov/compound/70678419
5	MK-3903	-10.7	1219737-12-8	https://pubchem.ncbi.nlm.nih.gov/compound/45256689
6	Tegobuvir	-10.6	1000787-75-6	https://pubchem.ncbi.nlm.nih.gov/compound/23649154
7	Rucaparib	-10.6	283173-50-2	https://pubchem.ncbi.nlm.nih.gov/compound/9931954
8	Rucaparib phosphate	-10.6	459868-92-9	https://pubchem.ncbi.nlm.nih.gov/compound/9931953
9	PF-5190457	-10.6	1334782-79-4	https://pubchem.ncbi.nlm.nih.gov/compound/58438464
10	Rebastinib	-10.6	1020172-07-9	https://pubchem.ncbi.nlm.nih.gov/compound/25066467
11	TPO agonist 1	-10.6	1033040-23-1	https://pubchem.ncbi.nlm.nih.gov/compound/135451068
12	ML385	-10.6	846557-71-9	https://pubchem.ncbi.nlm.nih.gov/compound/1383822
13	PCO371	-10.6	1613373-33-3	https://pubchem.ncbi.nlm.nih.gov/compound/76283707
14	Lifirafenib	-10.6	1446090-79-4	https://pubchem.ncbi.nlm.nih.gov/compound/89670174
15	MLKL-IN-2	-10.6	899759-16-1	https://pubchem.ncbi.nlm.nih.gov/compound/7593182
16	TNIK-IN-3	-10.6	2754265-25-1	https://pubchem.ncbi.nlm.nih.gov/compound/162641720
17	GS143	-10.5	916232-21-8	https://pubchem.ncbi.nlm.nih.gov/compound/121513876
18	TTP-8307	-10.5	950225-08-8	https://pubchem.ncbi.nlm.nih.gov/substance/478130590
19	EGFR-IN-8	-10.5	2407957-87-1	https://pubchem.ncbi.nlm.nih.gov/compound/139035057
20	SR-3306	-10.4	1128096-91-2	https://pubchem.ncbi.nlm.nih.gov/compound/57519510
21	GDC-0834 (S-enantiomer)	-10.4	1133432-50-4	https://pubchem.ncbi.nlm.nih.gov/compound/25234917
22	WWL70	-10.4	947669-91-2	https://pubchem.ncbi.nlm.nih.gov/compound/17759121
23	MY-5445	-10.4	78351-75-4	https://pubchem.ncbi.nlm.nih.gov/compound/1348
24	CCR6 inhibitor 1	-10.4	2437547-04-9	https://pubchem.ncbi.nlm.nih.gov/substance/443799049
25	ZXH-1-161	-10.4	2407654-51-5	https://pubchem.ncbi.nlm.nih.gov/compound/153385170
26	Bathophenanthroline	-10.4	1662-01-7	https://pubchem.ncbi.nlm.nih.gov/compound/72812

27	Chelidonine	-10.4	476-32-4	https://pubchem.ncbi.nlm.nih.gov/compound/197810
28	PH-797804	-10.3	586379-66-0	https://pubchem.ncbi.nlm.nih.gov/compound/22049997
29	PF-04457845	-10.3	1020315-31-4	https://pubchem.ncbi.nlm.nih.gov/compound/24771824
30	EPZ005687	-10.3	1396772-26-1	https://pubchem.ncbi.nlm.nih.gov/compound/60160561

Table S11. Sequence of siRNA that targets the specific gene

Names	Sense（5'-3'）	Antisense（5'-3'）
si-NC	UUCUCCGAACGUGUCACGUTT	ACGUGACACGUUCGGAGAATT
si-β-catenin	GCAGUUGUAAACUUGAUUATT	UAAUCAAGUUUACAACUGCTT
si-VEGFR1 isoform5	CCAGCGAGUACAAAGCUCUTT	AGAGCUUUGUACUCGCUGGTT
si-NANOG	GCAACCAGACCUGGAACAATT	UUGUUCCAGGUCUGGUUGCTT
si-POU5F1	UCCCAUGCAUUCAAACUGATT	UCAGUUUGAAUGCAUGGGATT
si-KDR	GGGCUUUACUAUUCCCAGCTT	GCUGGGAAUAGUAAAGCCCTT
si-HDAC9	GGACAGACCUCAGGAUGAUTT	AUCAUCCUGAGGUCUGUCCTT
si-DR5	CAGCCGUAGUCUUGAUUGUTT	ACAAUCAAGACUACGGCUGTT

Table S12. Sequences of primers for quantitative real-time PCR

Gene name	Forward primer (5'-3')	Reverse primer (5'-3')
CTNNB1	CACAAGCAGAGTGCTGAAGGTG	GATTCCTGAGAGTCCAAAGACAG
FZD1	GCTTTGTGTCGCTCTTCCGCAT	TACAGCACGCTGAAGACGCCAA
FZD3	GGCTCTCATAGTTGGCATTCCC	TGGAGTACCTGTCGGCTCTCAT
FZD7	GTCTTCAGCGTGCTCTACACAG	ACGGCATAGCTCTTGCACGTCT
FZD8	GCTCTACAACCGCGTCAAGACA	AAGGTGGACACGAAGCAGAGCA
LRP5	CCACAAGATCCTGGTGTCAGAG	TGTCCAGGTTGGCACGCTTGAT
LRP6	CAGTTGGAGTGGTGCTGAAAGG	CCATCCAAAGCAGCCCGTTCAA
DVL1	GCATAACCGACTCCACCATGTC	GATGGAGCCAATGTAGATGCCG
GSK3B	CCGACTAACACCACTGGAAGCT	AGGATGGTAGCCAGAGGTGGAT
CSNK1A1	GAAGATGTCCACGCCTGTTGAAG	GCGGAATAGCTGCCTCAGATAC
AXIN1	GTATGTGCAGGAGGTTATGCGG	CACCTTCCTCTGCGATCTTGTC
APC	AGGCTGCATGAGAGCACTTGTG	CACACTTCCAACTTCTCGCAACG
TCF7	CTGACCTCTCTGGCTTCTACTC	CAGAACCTAGCATCAAGGATGGG
TCF7L2	GAATCGTCCCAGAGTGATGTCG	TGCACTCAGCTACGACCTTTGC
LEF1	CTACCCATCCTCACTGTCAGTC	GGATGTTCCTGTTTGACCTGAGG
SOX17	ACGCTTTCATGGTGTGGGCTAAG	GTCAGCGCCTTCCACGACTTG
PLK2	CAACAATGGTGCTCACATGAGCC	GGAGCATCTGTTGCTGGGAAAAC
PTGS2	CGGTGAAACTCTGGCTAGACAG	GCAAACCGTAGATGCTCAGGGA
JAK1	GAGACAGGTCTCCCACAAACAC	GTGGTAAGGACATCGCTTTTCCG
SMAD1	TTGGCACAGTCTGTGAACCATGG	GTAACATCCTGGCGGTGGTATTC
HDAC9	TCTCGTCTCCAGGACTCACTCT	GCACTGGTGTTTCAGCATCAAGG
KDR	CCAGCAAAAGCAGGGAGTCTGT	TGTCTGTGTCATCGGAGTGATATCC
NRP1	AACAACGGCTCGGACTGGAAGA	GGTAGATCCTGATGAATCGCGTG
GATA2	CAGCAAGGCTCGTTCCTGTTCA	ATGAGTGGTCGGTTCTGCCCAT
SP1	ACGCTTCACACGTTCGGATGAG	TGACAGGTGGTCACTCCTCATG
NANOG	CTCCAACATCCTGAACCTCAGC	CGTCACACCATTGCTATTCTTCG
POU5F1	GGGGGTTCTATTTGGTGGGTT	TCCTCTTCATGGGTGAGGGTA
FLT1(P1)	AAAGCCACCAACCAGAAGGG	GCCACACAGGTGCATGTTAG
FLT1(P2)	ACCTGGACTGACAGCAAACC	CACCGAGTTGTAGTCTGGGG
ACTB	CTTCGCGGGCGACGAT	CCACATAGGAATCCTTCTGACC

Supplementary Methods

Construction and transfection of expression plasmids

The eukaryotic expression plasmid for overexpressing Flag-β-catenin WT was obtained from GeneChem (Shanghai, China) and the site-directed Flag-β-catenin mutants were constructed using Mut Express®MultiS or the Mut Express®II Fast Mutagenesis Kit V2 (Vazyme, China, Cat# C215; C214) according to the manufacturer's protocol. Eukaryotic expression plasmids for HA-VEGFR1 were constructed based on a routine molecular cloning method provided by Vazyme (Nanjing, China), including cDNA Synthesis (Cat# R312), DNA amplification (Cat# P505) and DNA recombination (Cat# C112). All constructed plasmids were sequenced, and the correct clones were selected for the next experiments. The following transfection of plasmids was performed using Lipofectamine® 2000 Reagent (Invitrogen) in accordance with the manufacturer's instruction. Due to the low efficiency and high toxicity of plasmid DNA transfection in HUVECs, the experiment with plasmid transfection was performed in 293T cells. Briefly, 293T cells were cultured to 70%-90% confluence and then added with DNA-lipid complex in Opti-MEM for 48 hours, then subjected to the subsequent treatments and detections.

Transfection of siRNA and transduction of adenovirus

Transfection of siRNA was conducted following the instruction provided by GenePharma (Shanghai, China). In Brief, HUVECs were seeded in 6-well plates with 40%–50% confluence, and transfected with specific siRNA or negative control (NC) siRNA (25 nmol/L) in serum-free, antibiotic-free endothelial cell medium containing 5 uL siRNA-Mate (GenePharma, Cat# G04002). The sequences of siRNA are shown in Table S11.

Adenovirus transduction was performed under the protocol offered by GeneChem (Shanghai, China). In brief, HUVECs were cultured to 40%–50% confluence and transduced with ad-β-catenin WT, ad-β-catenin Y142F at multiplicities of infection (MOI) of about 50. After 48 h, cells were performed with the subsequent experiments.

Protein preparation and western blot analysis

Total proteins were extracted by using lysis buffer supplemented with phosphatase and protease inhibitor cocktail. Nuclear proteins were prepared with the Nuclear Protein Extraction

Kit (Beyotime) under the manufacturer's instruction. The protein concentration was quantified by the bicinchoninic acid (BCA) kit (GBCBIO, China). Protein extracts were subjected to SDS-PAGE and transferred to polyvinylidene difluoride (PVDF, Merck Millipore, USA) membranes, which were then blocked with 5% bovine serum albumin or 5% skimmed milk for 1 h at room temperature and incubated with specific primary antibodies overnight at 4°C, followed by being incubated with HRP-conjugated secondary antibodies for 1 h at room temperature. The band was visualized by chemiluminescence and quantified by ImageJ.

Co-Immunoprecipitation

HUVECs or 293T cells were collected and lysed using Cell Lysis Buffer for Immunoprecipitation (IP) (Beyotime, China, Cat# P0013). The lysate was obtained and then incubated with β-catenin antibody or HA tag antibody with a volume ratio of 1:100 overnight at 4 °C and then added with 20 μL Protein A+G Magnetic Beads (Beyotime, China) at 4 °C for 2 h. Beads were then washed with PBS three times and resuspended in 1×SDS sample buffer, followed by western blotting with a specific antibody against the β-catenin or HA tag.

Quantitative real-time PCR (qPCR)

Total RNA was prepared from HUVECs with TRIzol reagent (GBCBIO, China) under the manufacturer's protocol. Complementary DNA synthesis was performed using HiScript® III RT SuperMix (Vazyme, China). Real-time PCR was conducted in a 7500 Real-Time PCR Platform (Applied Biosystems, USA) with specific primers. The primers used for qPCR are listed in Table S12. Data were normalized to the expression of a control gene (*ACTB*) and the $2^{-\Delta\Delta Ct}$ method was applied to quantify mRNA expression.
