# Supplementary material for: Endothelial β-catenin upregulation and Y142 phosphorylation drive diabetic angiogenesis via upregulating KDR/HDAC9
Source: Cell Commun Signal. 2024 Mar 15;22:182. doi: 10.1186/s12964-024-01566-1 (PMC10941375; doi:10.1186/s12964-024-01566-1)

Full uncropped gel image for Fig.2D

β-catenin

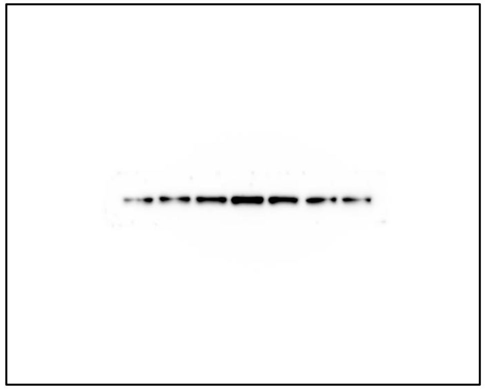

β-actin

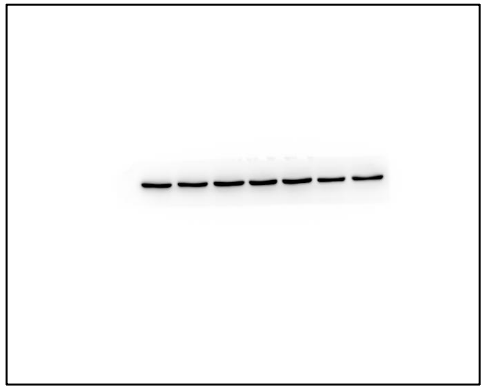

Full uncropped gel image for Fig.2H

β-catenin

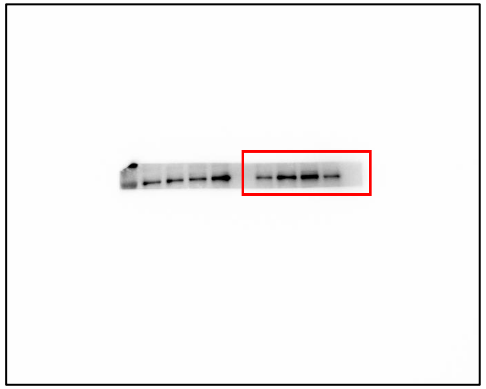

Lamin B1

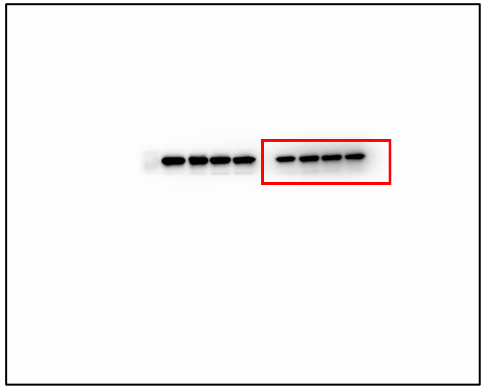

Full uncropped gel image for Fig.4E

Ctrl

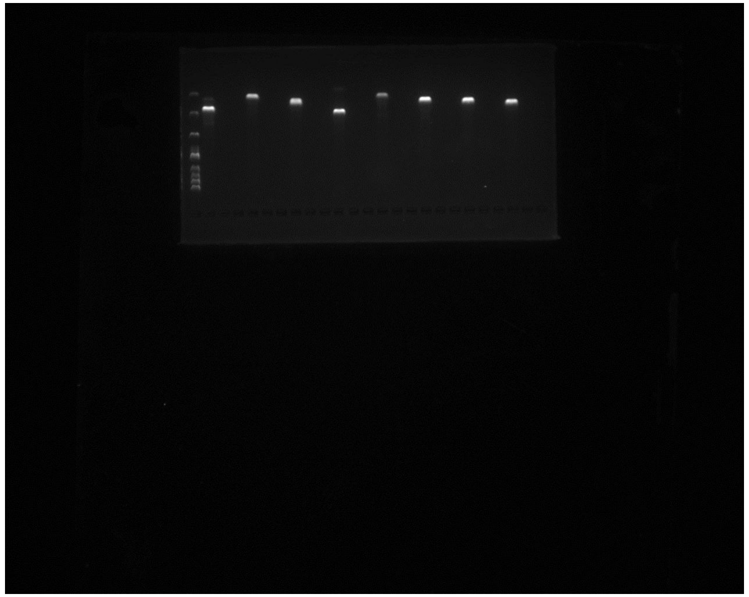

AGEs

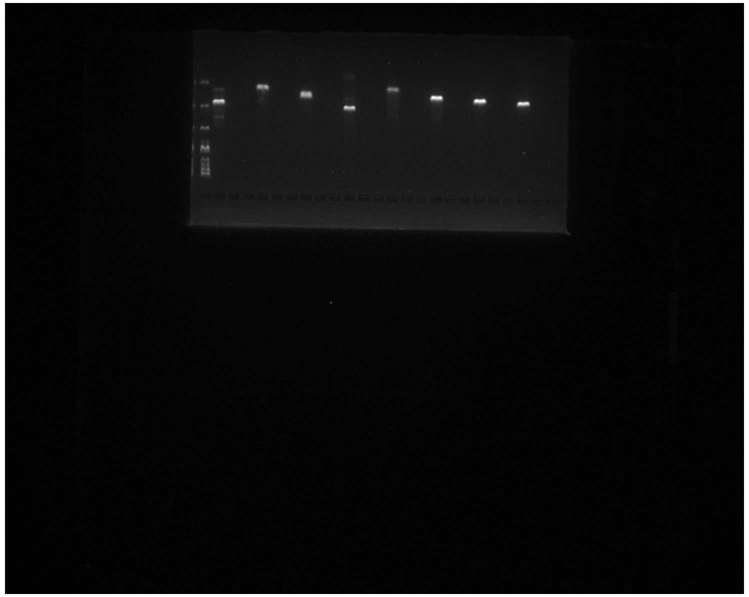

Full uncropped gel image for Fig.5A

IP: β-catenin

Phospho-Tyr

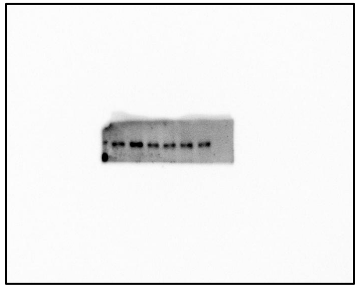

Phospho-Ser/Thr

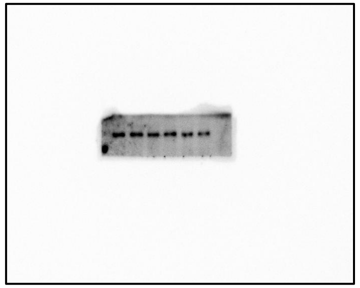

β-catenin

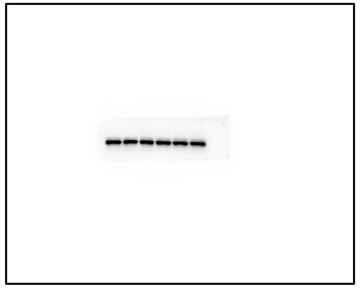

Input

Phospho-Tyr

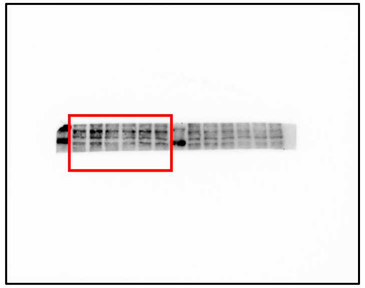

Phospho-Ser/Thr

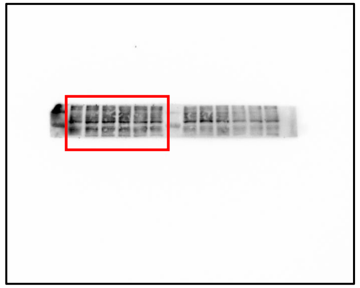

β-catenin

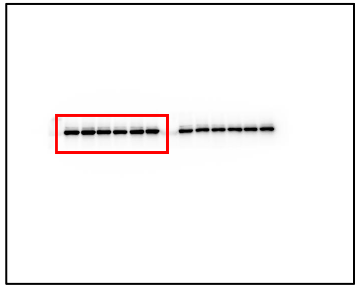

β-actin

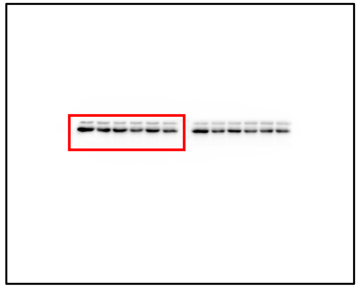

Full uncropped gel image for Fig.5C

IP: Flag

Phospho-Tyr

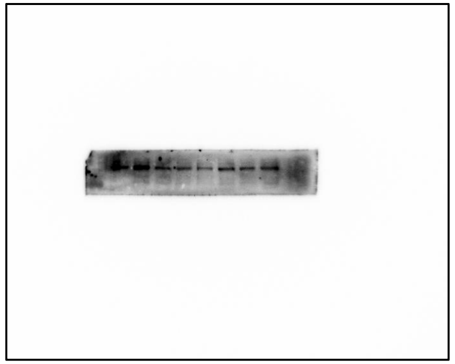

Flag-β-catenin

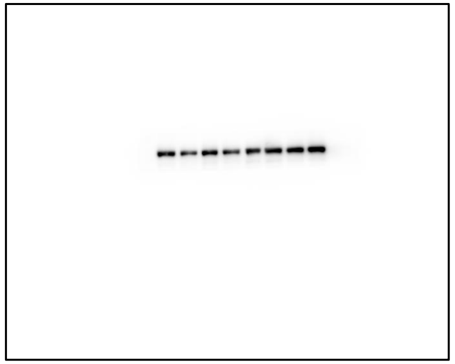

Input

Phospho-Tyr

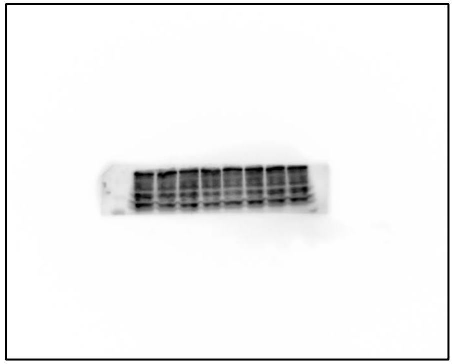

Flag-β-catenin

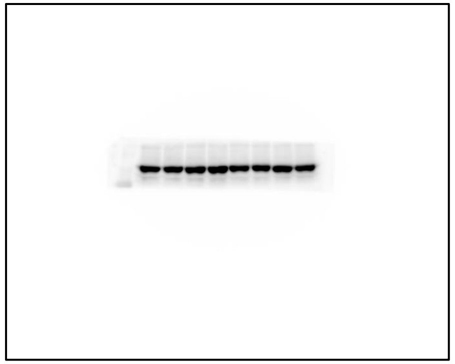

β-actin

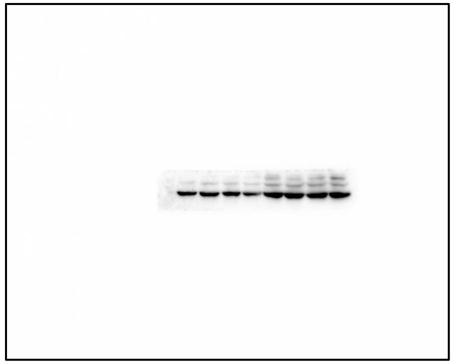

Full uncropped gel image for Fig.5E

IP: Flag

Input

Phospho-Tyr

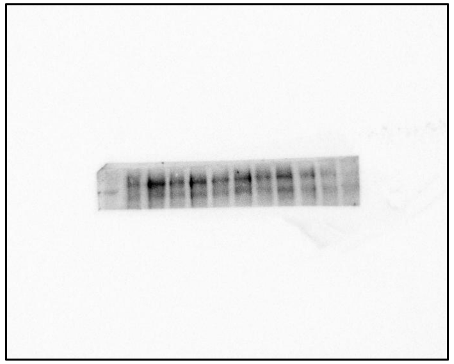

Flag-β-catenin

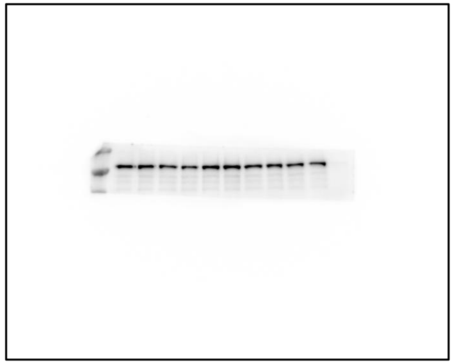

Phospho-Tyr

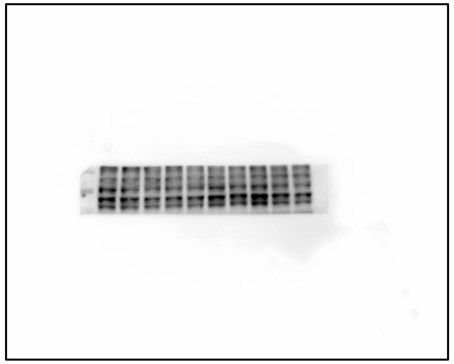

Flag-β-catenin

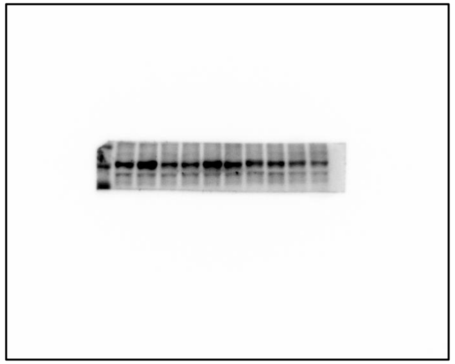

β-actin

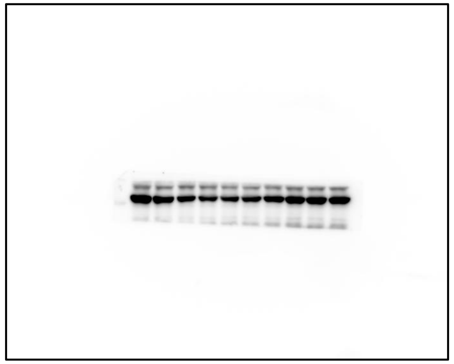

Full uncropped gel image for Fig.5G

p-β-catenin Y142

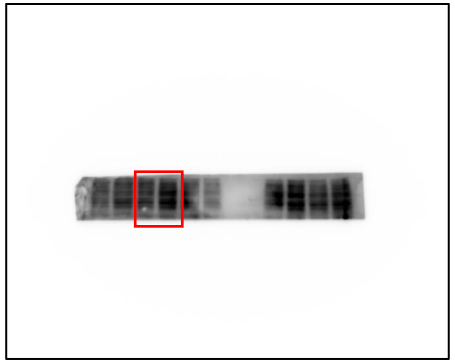

β-catenin

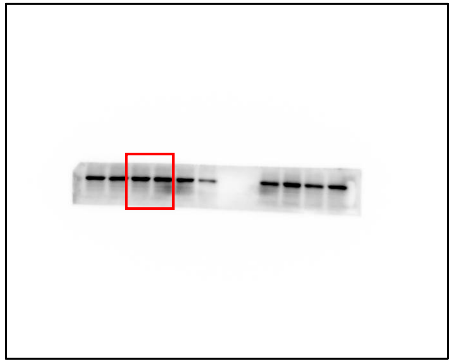

β-actin

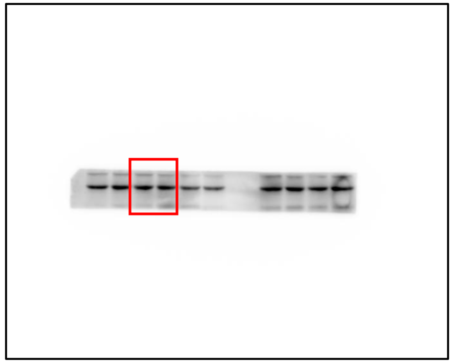

Full uncropped gel image for Fig.6A

NL

Flag-β-catenin

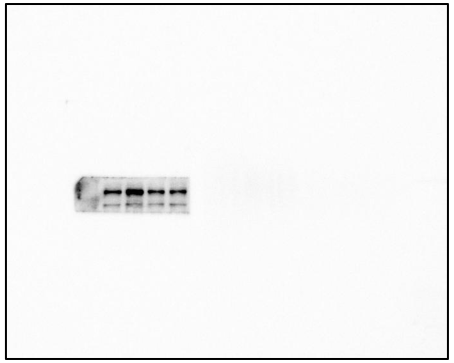

Lamin B1

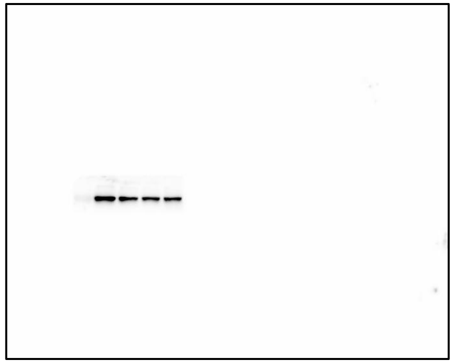

WCL

Flag-β-catenin

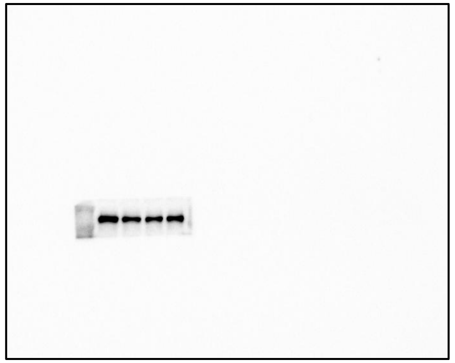

β-actin

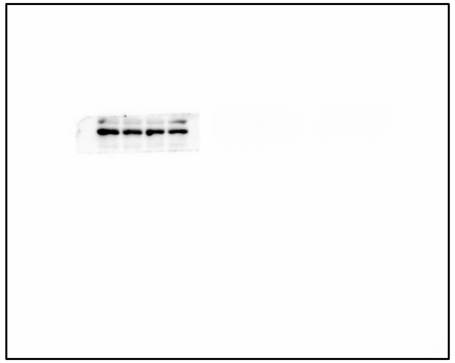

Full uncropped gel image for Fig.7C

Full-length VEGFR1

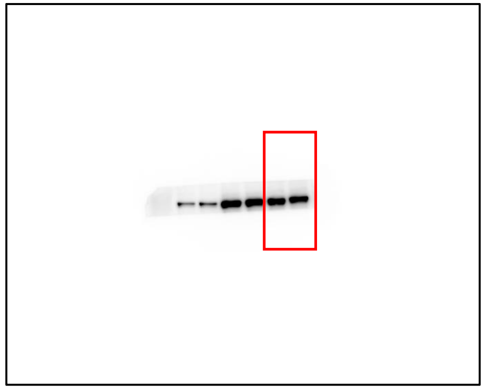

β-actin

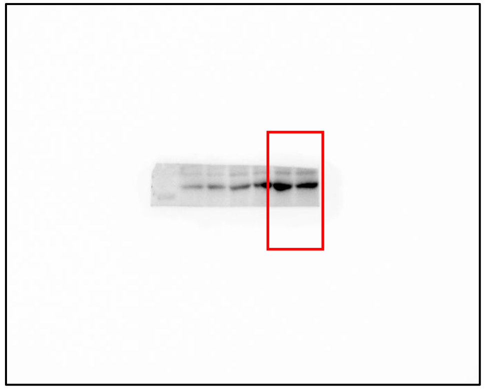

Full uncropped gel image for Fig.7D

Full-length VEGFR1

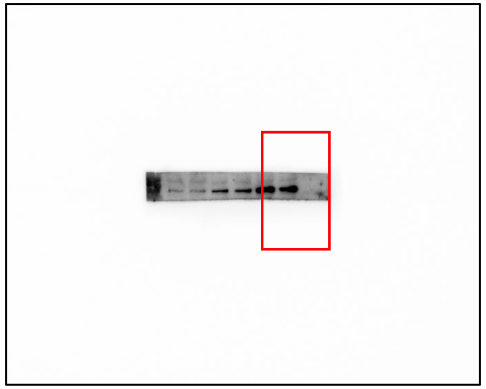

β-catenin

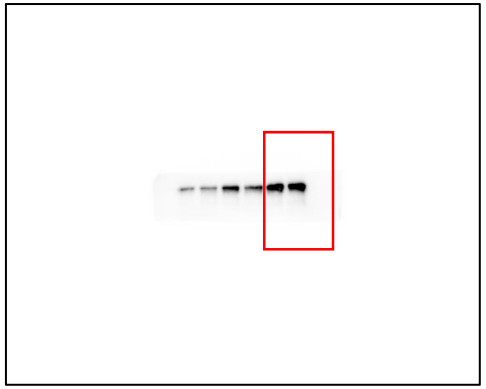

Full uncropped gel image for Fig.7E

VEGFR1 isoform5
VEGFR1 isoform6
VEGFR1 isoform7

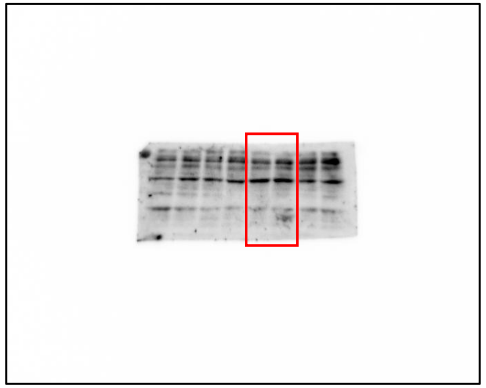

β-actin

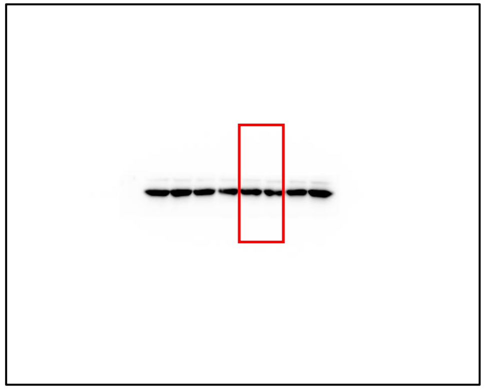

Full uncropped gel image for Fig.7F

VEGFR1 isoform5
VEGFR1 isoform6
Heavy Chain
VEGFR1 isoform7

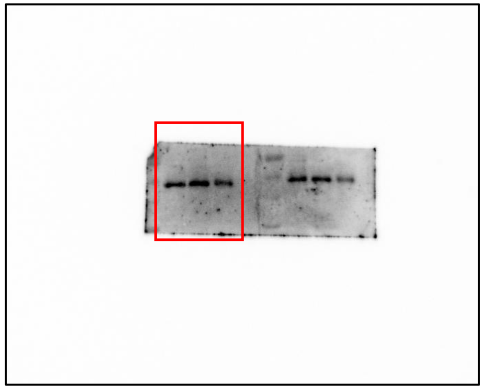

β-catenin

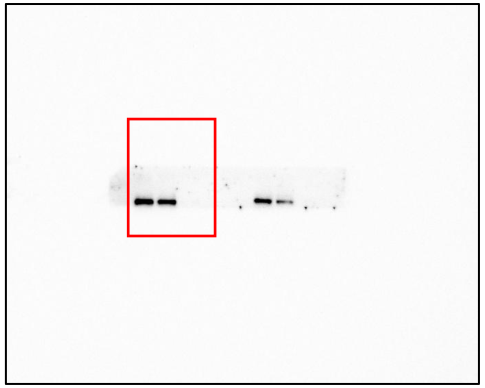

Full uncropped gel image for Fig.7G

IP: HA

β-catenin

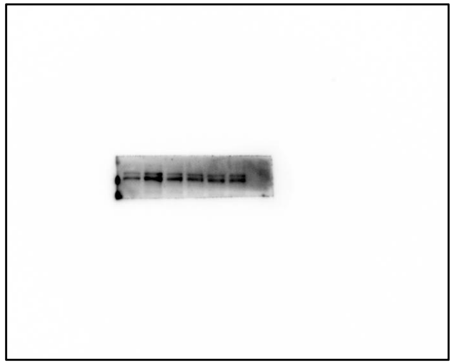

HA-VEGFR1 isoform5
HA-VEGFR1 isoform6
HA-VEGFR1 isoform7

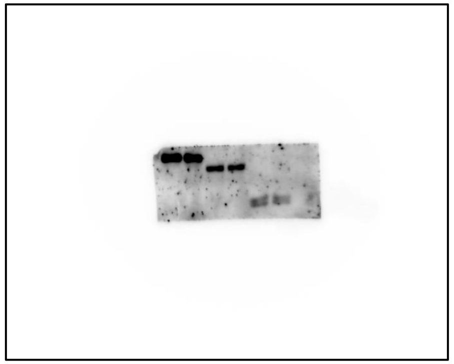

Input

β-catenin

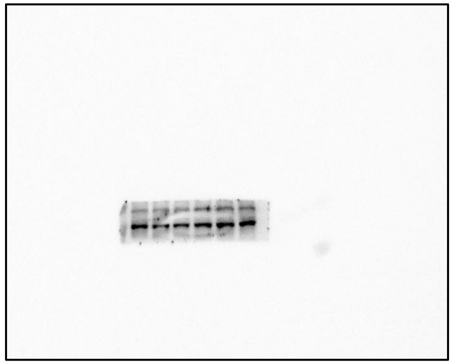

HA-VEGFR1 isoform5
HA-VEGFR1 isoform6
HA-VEGFR1 isoform7

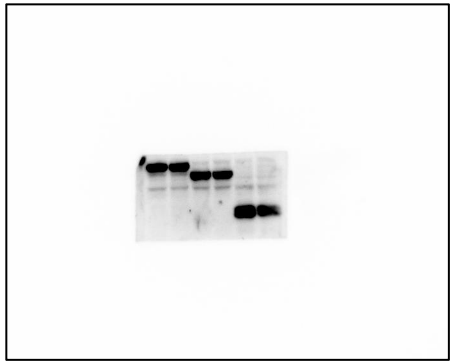

β-actin

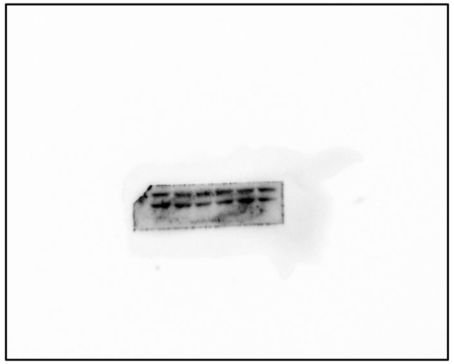

Full uncropped gel image for Fig.7H

IP: HA

β-catenin

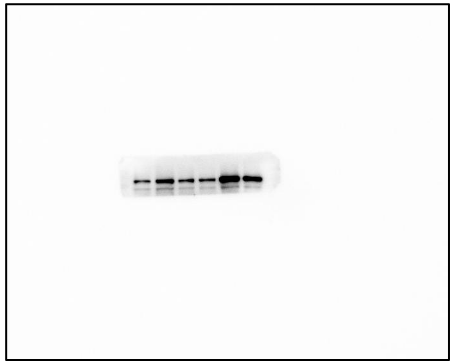

HA-VEGFR1 isoform5
HA-VEGFR1 isoform6
HA-VEGFR1 isoform7

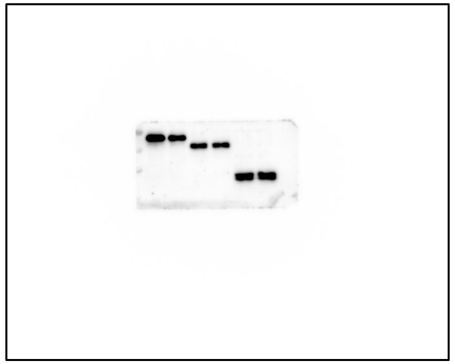

Input

β-catenin

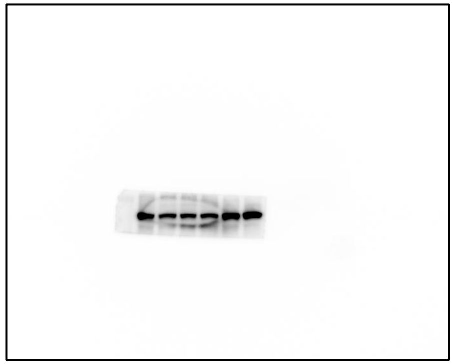

HA-VEGFR1 isoform5
HA-VEGFR1 isoform6
HA-VEGFR1 isoform7

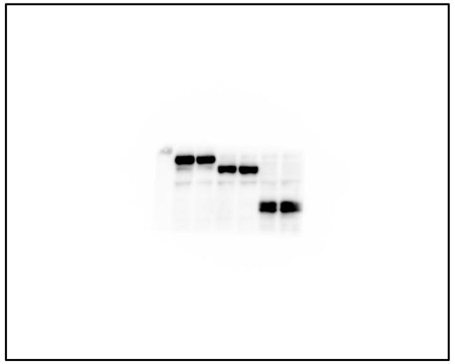

β-actin

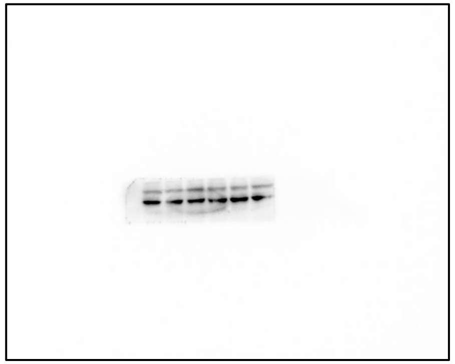

Full uncropped gel image for Fig.7I

p-β-catenin Y142

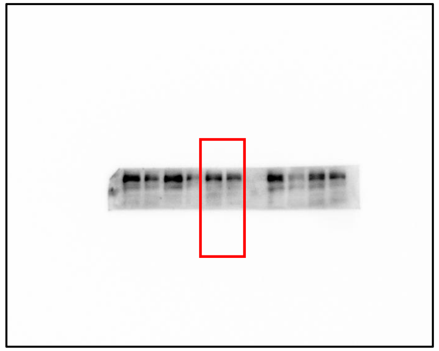

β -catenin

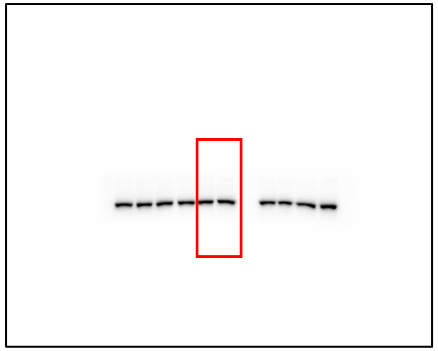

VEGFR1 isoform5

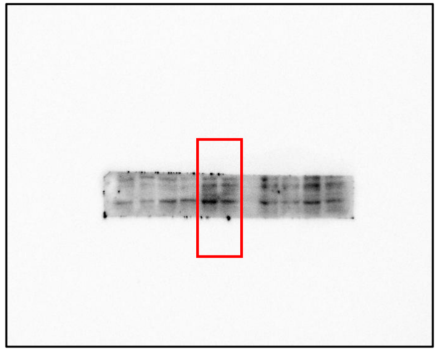

β-actin

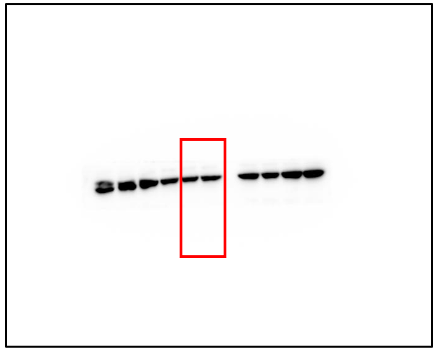

Full uncropped gel image for Fig.7J

p-β-catenin Y142

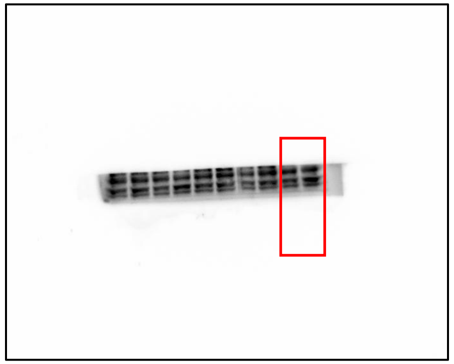

β -catenin

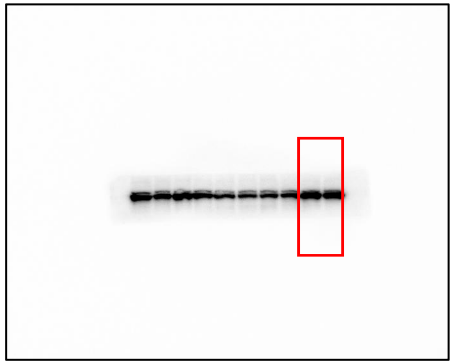

HA-VEGFR1 isoform5

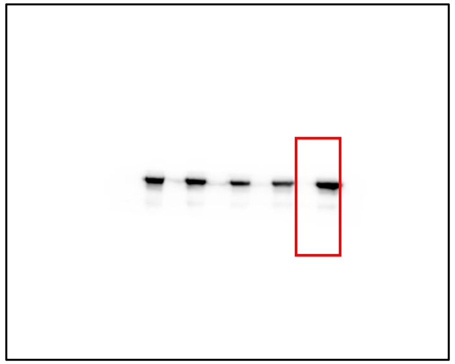

β-actin

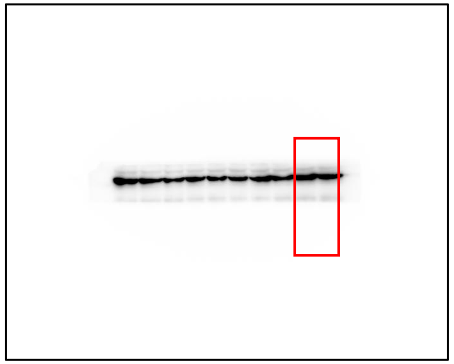

Full uncropped gel image for Fig.8C

IP: HA

Input

Flag-β-catenin

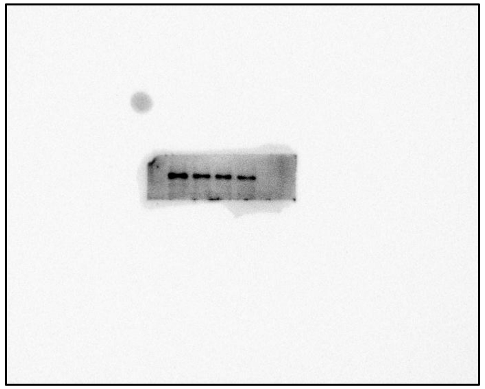

HA-VEGFR1 isoform5

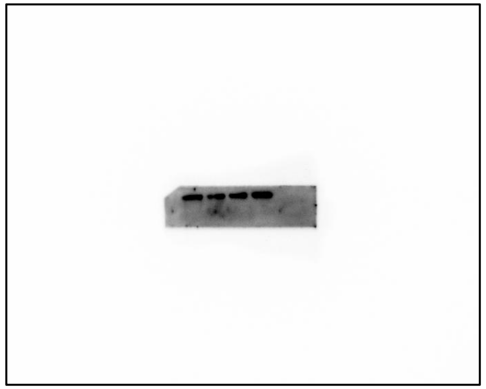

Flag-β-catenin

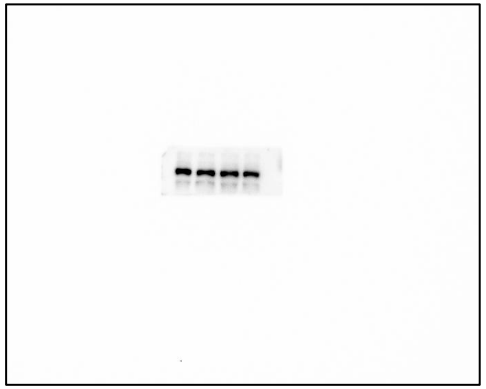

HA-VEGFR1 isoform5

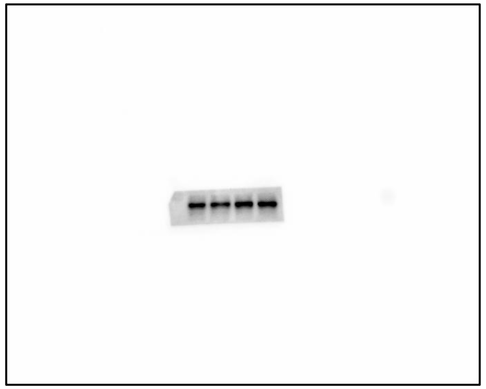

β-actin

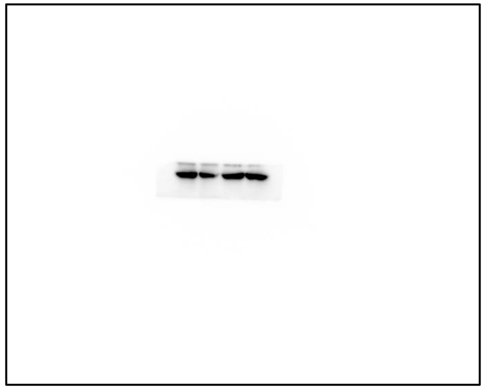

Full uncropped gel image for Fig.8G

p-β-catenin Y142

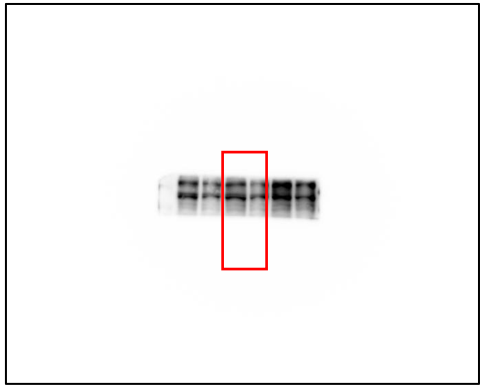

β -catenin

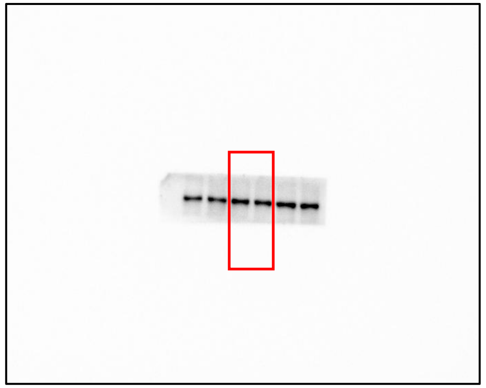

β -actin

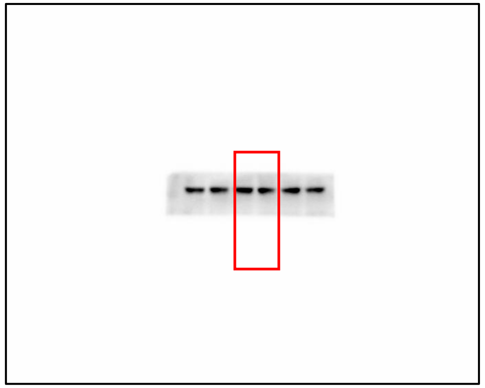

Full uncropped gel image for Fig. S2E

Ctrl

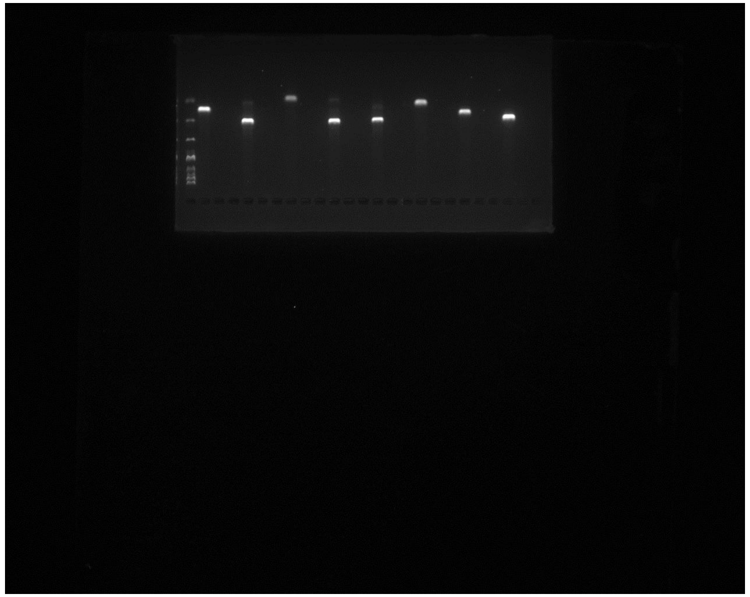

AGEs

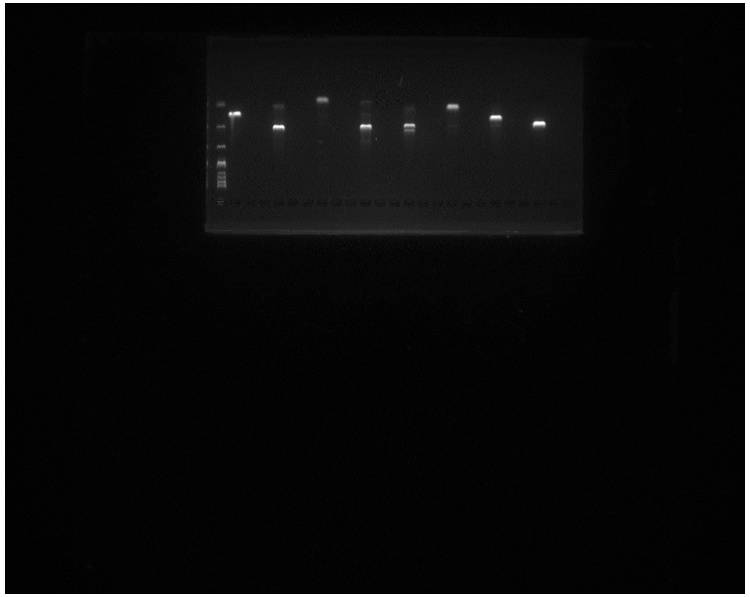

Full uncropped gel image for Fig. S7A

IP: HA

Flag-β-catenin

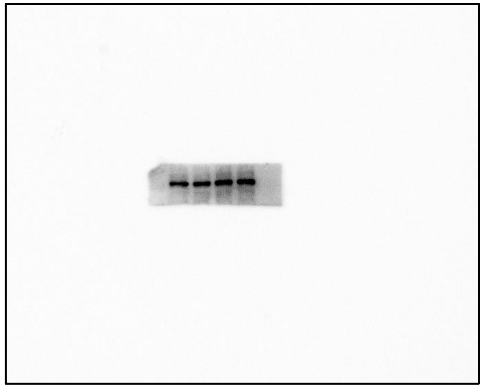

HA-VEGFR1 isoform5

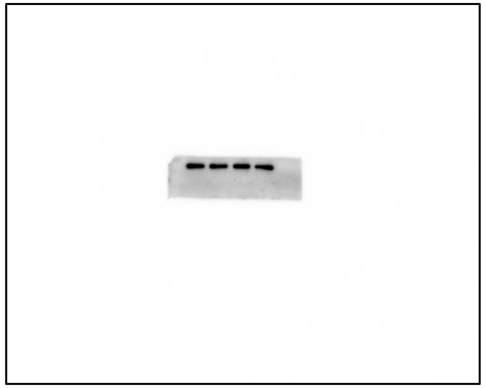

Input

Flag-β-catenin

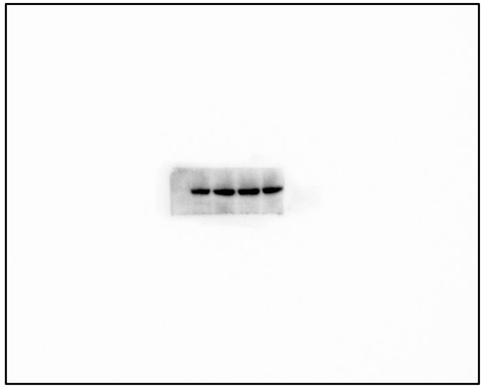

HA-VEGFR1 isoform5

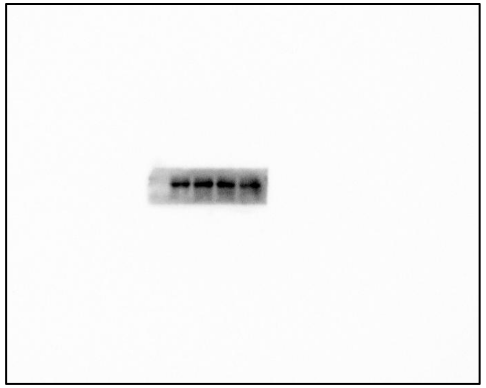

β-actin

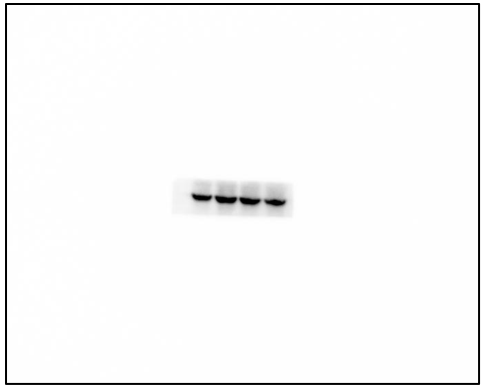

Full uncropped gel image for Fig. S7B

IP: HA

Flag-β-catenin

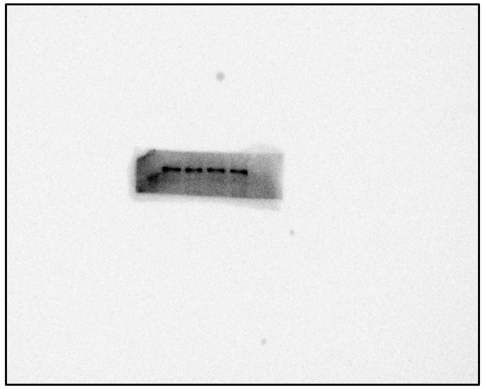

HA-VEGFR1 isoform5

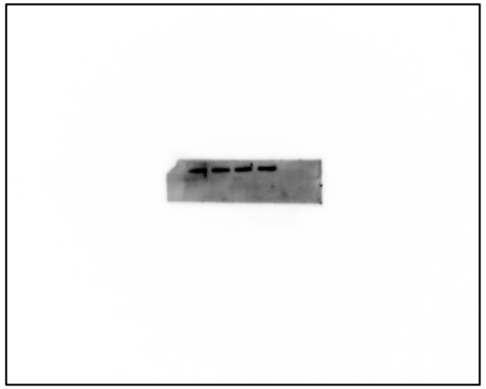

Input

Flag-β-catenin

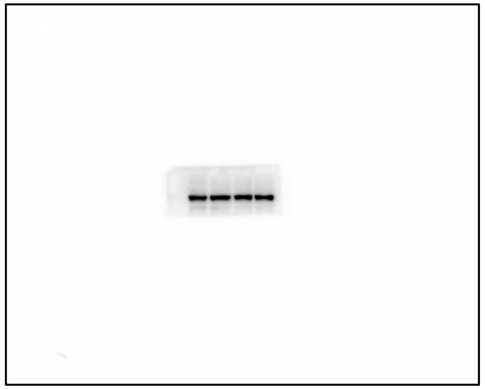

HA-VEGFR1 isoform5

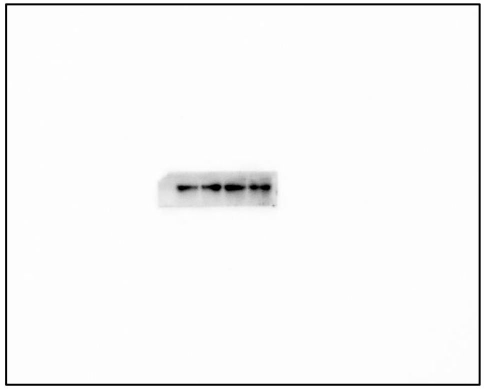

β-actin

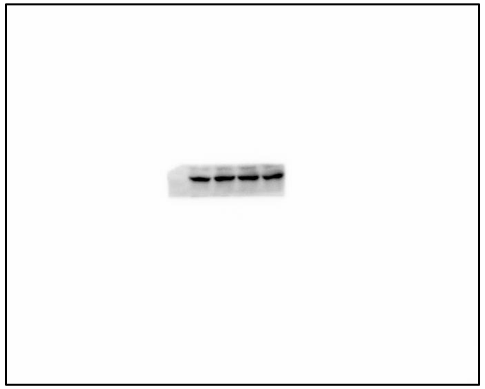

Full uncropped gel image for Fig. S7C

IP: HA

Flag-β-catenin

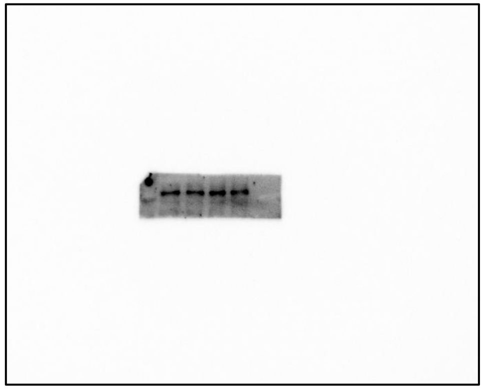

HA-VEGFR1 isoform5

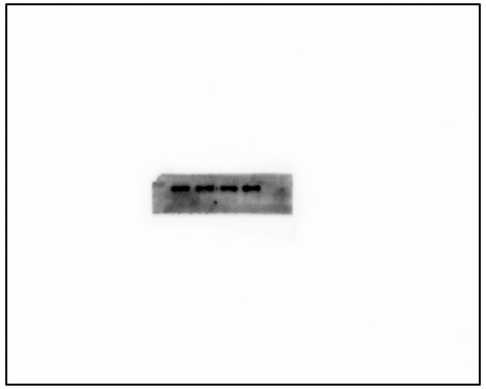

Input

Flag-β-catenin

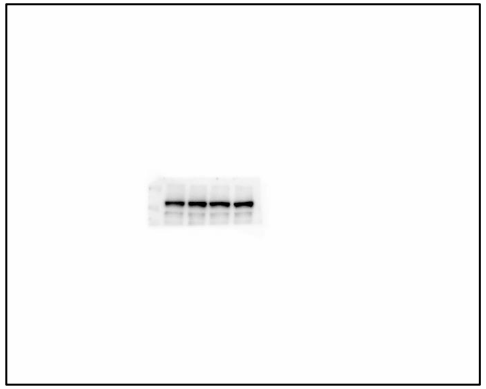

HA-VEGFR1 isoform5

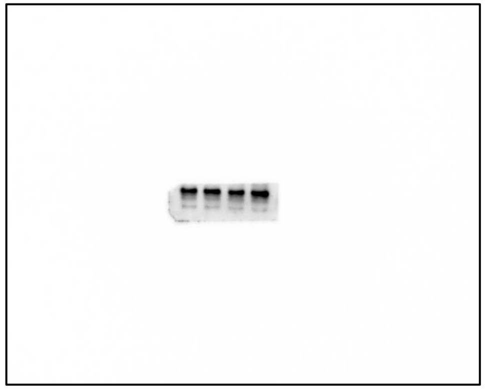

β-actin

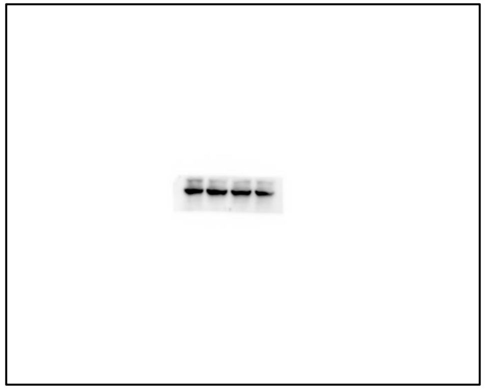

Full uncropped gel image for Fig. S7D

IP: HA

Flag-β-catenin

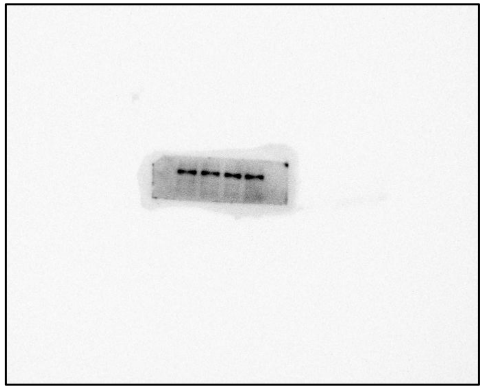

HA-VEGFR1 isoform5

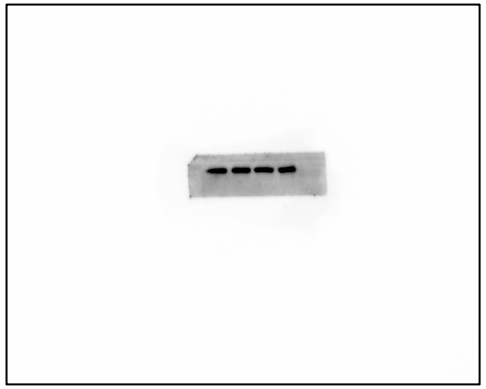

Input

Flag-β-catenin

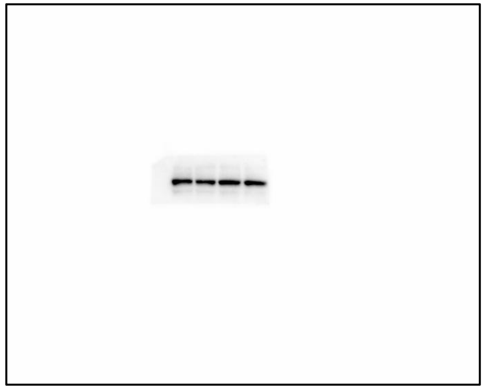

HA-VEGFR1 isoform5

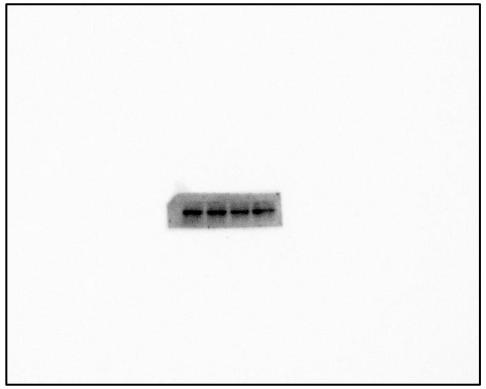

β-actin

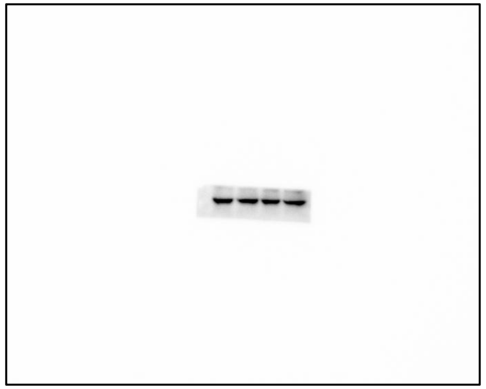

Full uncropped gel image for Fig. S8A

DR5

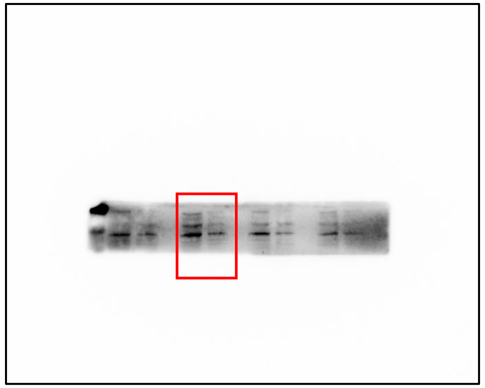

β-actin

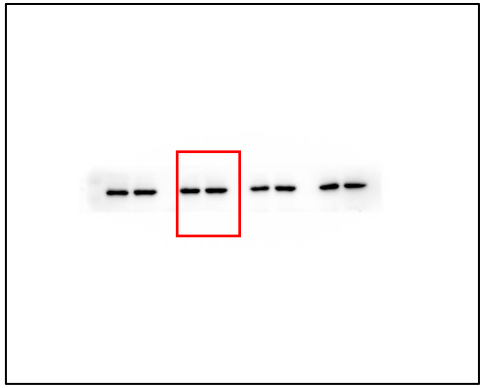

Full uncropped gel image for Fig. S9F

β-catenin

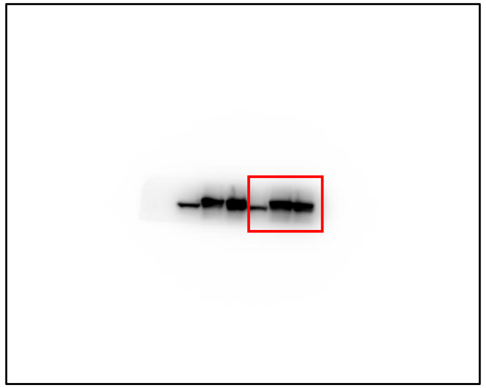

β-actin

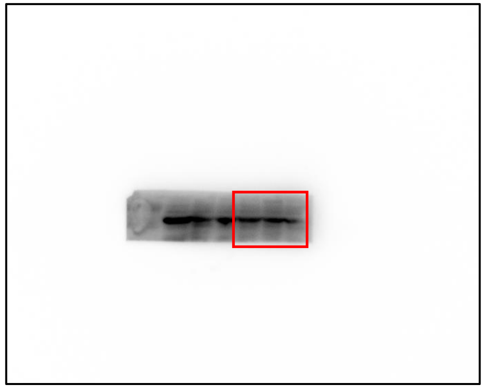

Full uncropped gel image for Fig. S9G

VEGFR1 isoform1

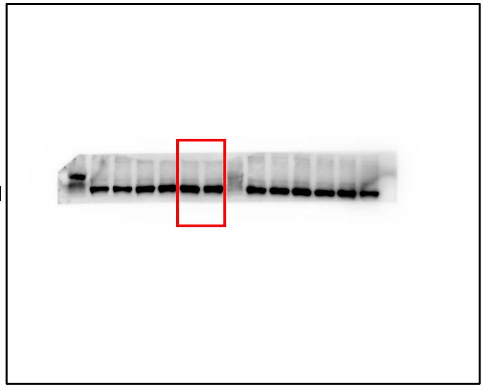

VEGFR1 isoform6

VEGFR1 isoform7

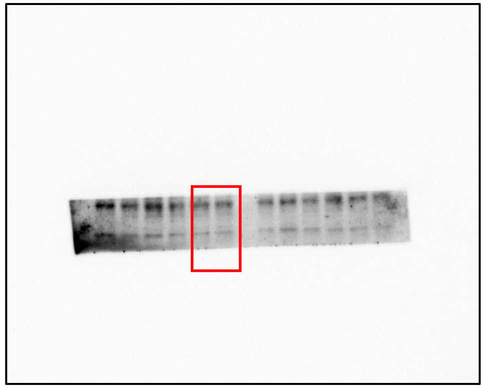

β-actin

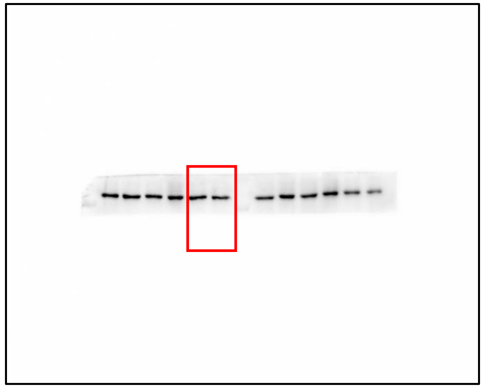

Supplement: Supplementary file 2 — Supplementary Material 2. [file 12964_2024_1566_MOESM2_ESM.pdf]
